# Piceatannol Promotes Burn Wound Healing by Coordinately Modulating Inflammation–Oxidative Stress Crosstalk, Angiogenesis, and Fibrotic Remodeling

**DOI:** 10.3390/biom16070926

**Published:** 2026-06-23

**Authors:** Jingbo Wang, Boyu Liao, Yijing Ma, Yihan Yang, Yiyang Cao, Xin Huang, Tianxin Wen, Hai-Shu Lin

**Affiliations:** College of Pharmacy, Shenzhen Technology University, Shenzhen 518118, China

**Keywords:** piceatannol, resveratrol, burn wound healing, inflammation–oxidative stress crosstalk, fibroblast-to-myofibroblast transition, STAT3–VEGF pathway

## Abstract

Burn wound healing is a complex and dynamic process involving coordinated regulation of inflammation, oxidative stress, angiogenesis, and tissue remodeling. *Polygonum cuspidatum*, a traditional Chinese medicinal herb widely used for trauma- and inflammation-related disorders, represents an important source of bioactive compounds for tissue repair. Piceatannol (PIC), a naturally occurring stilbene constituent of *P. cuspidatum*, possesses potent anti-inflammatory and antioxidant activities; however, its therapeutic potential in burn wound healing remains insufficiently understood. In the present study, the therapeutic effects and underlying mechanisms of topical PIC were investigated using a murine deep second-degree burn model combined with multiple skin-related cellular models, including keratinocytes, fibroblasts, endothelial cells, and macrophages. PIC markedly accelerated wound closure and improved histological architecture, as evidenced by reduced inflammatory infiltration, enhanced collagen organization, and increased neovascularization. Mechanistically, PIC suppressed NF-κB activation and modulated KEAP1/NRF2-associated redox signaling, thereby alleviating inflammation–oxidative stress crosstalk during wound healing. In keratinocyte–fibroblast co-culture systems, PIC inhibited fibroblast-to-myofibroblast transition, reduced α-smooth muscle actin (α-SMA) expression, and attenuated excessive collagen deposition, suggesting anti-fibrotic activity. In addition, PIC promoted endothelial tube formation through activation of the STAT3–VEGF signaling axis. Collectively, these findings demonstrate that PIC facilitates burn wound repair through coordinated anti-inflammatory, antioxidative, pro-angiogenic, and anti-fibrotic effects. This study provides pharmacological support for the therapeutic potential of *P. cuspidatum*-derived compounds in burn management and highlights PIC as a promising candidate for topical treatment of burn injuries.

## 1. Introduction

Burn injuries remain a major clinical challenge worldwide, often resulting in prolonged healing, infection, and excessive scar formation that severely impair functional and aesthetic outcomes [1,2]. Effective burn wound repair requires the coordinated regulation of inflammation, oxidative stress, angiogenesis, and extracellular matrix remodeling, and disruption of this tightly controlled process frequently leads to delayed re-epithelialization, impaired neovascularization, and pathological fibrosis [2,3].

Excessive and sustained inflammatory responses play a central role in burn-induced tissue damage and delayed wound healing [2,3]. Activation of pro-inflammatory signaling pathways drives the excessive production of inflammatory mediators, which in turn amplifies oxidative stress and establishes a self-perpetuating inflammatory–oxidative cycle [2,3,4]. Accumulation of oxidative stress disrupts redox homeostasis and impairs endogenous antioxidant defense systems, thereby exacerbating tissue injury and delaying effective wound repair [2,3,4]. In parallel, persistent inflammation contributes to aberrant fibroproliferative remodeling and excessive extracellular matrix deposition, thereby causing predisposition to pathological scar formation following burn injury [2,5]. Accordingly, therapeutic agents capable of simultaneously attenuating inflammation and oxidative injury while supporting vascular regeneration and balanced matrix remodeling are of considerable interest for burn wound management.

Angiogenesis represents another indispensable component of successful burn wound healing [2]. Insufficient or dysregulated vascular regeneration compromises oxygen and nutrient delivery to regenerating tissues, thereby delaying repair and impairing tissue quality [2,3]. Among the signaling pathways governing angiogenesis, the signal transducer and activator of transcription 3 (STAT3)–vascular endothelial growth factor (VEGF) axis is a critical regulator of endothelial cell proliferation, migration, and tube formation [6,7]. Therapeutic strategies that restore angiogenesis while restraining inflammation-driven fibrosis are therefore highly desirable but remain limited [5].

**Figure 1:**
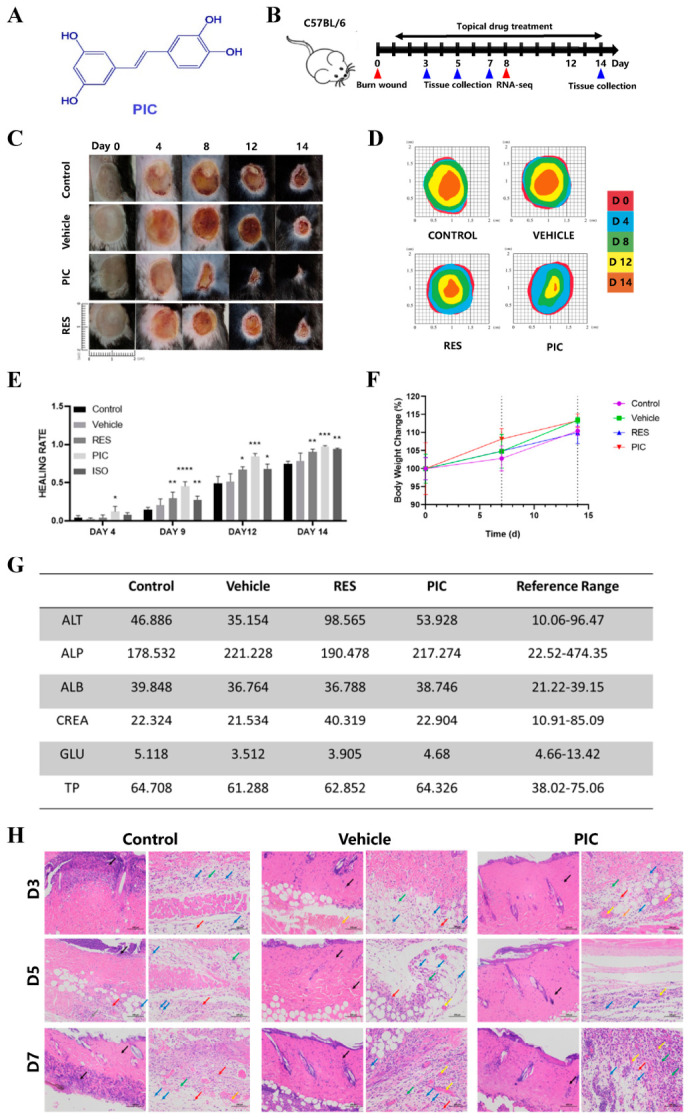
Piceatannol promotes burn wound healing and tissue repair in mice. (**A**) Chemical structure of PIC. (**B**) Schematic diagram of the experimental timeline, including burn induction, treatment regimen, and sample collection points. (**C**) Representative macroscopic photographs of dorsal burn wounds in mice from the Vehicle (white petrolatum), Positive Control (RES), and PIC-treated groups at the indicated days post-injury. (**D**) Color-coded overlay maps of wound contours reconstructed from representative macroscopic wound photographs. For each group, wound margins at the indicated time points were manually delineated from calibrated images using image-analysis software under the same scale. The extracted wound outlines were then filled with distinct colors corresponding to each day and overlaid sequentially from day 0 to day 14. This visualization illustrates the progressive reduction in wound area during healing and facilitates direct comparison of wound-closure dynamics among groups. (**E**) Quantitative analysis of wound closure rate over time. (**F**) Body weight change curves of mice in different treatment groups throughout the experimental period. (**G**) Blood biochemical indices of mice in different groups. (**H**) Representative hematoxylin and eosin (H&E)-stained sections of burn wound tissues. Histopathological features are indicated as follows: black arrows, detached sheets of necrotic cells; yellow arrows, necrotic cellular debris in the dermis; gray arrows, focal necrosis of subcutaneous fibrous tissue; red arrows, fibroconnective tissue proliferation in the subcutaneous layer; blue arrows, infiltrating macrophages; green arrows, neovascularization; orange arrow: loose tissue architecture with mild hemorrhage. Scale bar = 200 μm. * *p* < 0.05, ** *p* < 0.01, *** *p* < 0.001, **** *p* < 0.0001 vs. Control group.

Within this context, phytochemicals with pleiotropic but well-defined biological activities offer a rational therapeutic option for complex wound-healing disorders [8,9]. Natural polyphenols have attracted considerable attention as multi-target therapeutics due to their broad bioactivities and generally favorable safety profiles [10,11]. Piceatannol (*trans*-3,5,3′,4′-tetrahydroxystilbene, PIC, Figure 1A), a dietary stilbene found in grapes, passion fruit, peanuts, and other plant sources, is also a hydroxylated metabolite of resveratrol (*trans*-3,5,4′-trihydroxystilbene, RES) and exhibits stronger biological activity than its parent compound [12,13,14]. Previous studies have demonstrated its anti-inflammatory and antioxidant activities, together with metabolic, vascular-protective, and cytoprotective effects in different experimental settings, as well as favorable tolerability profiles observed in human studies using PIC or PIC-rich botanical extracts [15,16]. These properties suggest that PIC may be particularly suitable for intervening in the multifactorial pathological milieu of burn wounds. However, despite these promising features, the therapeutic effects of PIC on burn wound healing and its integrated mechanisms involving inflammation, oxidative stress, angiogenesis, and fibrotic remodeling have not been systematically elucidated.

In the present study, we investigated the wound-healing potential of PIC using a murine deep second-degree burn model in combination with mechanistic in vitro assays. By employing human keratinocytes, fibroblasts, endothelial cells, and keratinocyte–fibroblast co-culture systems, we aimed to elucidate how PIC coordinately modulates inflammation–oxidative stress crosstalk, angiogenic signaling, and fibrotic responses. Our findings identify PIC as a multifunctional bioactive stilbene that accelerates burn wound repair through integrated regulation of inflammatory, oxidative, angiogenic, and profibrotic processes, thereby supporting its potential as a candidate agent for burn wound management.

## 2. Materials and Methods

### 2.1. Chemicals and Reagents

Piceatannol (*trans*-3,5,3′,4′-tetrahydroxystilbene, PIC, Figure 1A) and resveratrol (*trans*-3,5,4′-trihydroxystilbene, RES) were purchased from Macklin Reagents (Shanghai, China) and Tokyo Chemical Industry (Tokyo, Japan), respectively. Antibodies against COX-2 (12282T, 1:2000), iNOS (13120S, 1:1000), p65 (8242T, 1:1000), and phosphorylated p65 (P-p65; 3033T, 1:1000) were obtained from Cell Signaling Technology (Danvers, MA, USA). Antibodies against caspase-1 (81482-1-RR, 1:2000), Keap1 (10503-2-AP, 1:1000), VEGF (19003-1-AP, 1:1000), and GAPDH (81640-5-RR, 1:2000) were purchased from Proteintech (Wuhan, China). Antibodies against collagen I and collagen III (LCS-Ab-17959, 1:1000) as well as α-SMA (LCS-mAb-03220) were obtained from LunChangShuo Biotech (Xiamen, China). Antibodies against STAT3 (M900014, 1:1000) and phosphorylated STAT3 (P-STAT3; R013862, 1:1000) were purchased from Epizyme Biotech (Shanghai, China). The lipopolysaccharide (LPS; *Escherichia coli* 0111:B4) was obtained from Merck MilliporeSigma (St. Louis, MO, USA). All other reagents and chemicals used in this study were of analytical grade or higher.

### 2.2. Preparation of Topical Burn Ointment

The topical ointment formulations were prepared as follows: pharmaceutical-grade white petrolatum was selected as the base vehicle because of its high purity, physicochemical stability, and dermal safety. Briefly, white petrolatum was molten in a clean glass beaker using a water bath maintained at 60 °C under gentle stirring. PIC or RES powder was then gradually added to the molten petrolatum and mixed thoroughly until a homogeneous ointment was obtained at a nominal concentration equivalent to 30 μM (7.32 or 6.84 μg/mL). After cooling to room temperature, the formulations were transferred into sterile vials and stored at 2–8 °C, protected from light, until use.

### 2.3. Animals

All animal experiments were conducted in accordance with the ARRIVE guidelines [17]. The study design and animal handling protocol reviewed and approved by the Ethics Committee of Shenzhen Technology University (approval No. SZTUDWLL2025025; 5 March 2025).

All experiments were performed in the specific pathogen-free (SPF) animal facilities of Shenzhen Glorybay Biotech Co., Ltd. (Shenzhen, China) under controlled environmental conditions (22 ± 2 °C, 50 ± 10% relative humidity) with a 12 h light/dark cycle and free access to standard chow and water. SPF male C57BL/6 mice (6–8 weeks old, 18–22 g) were obtained through Shenzhen Glorybay Biotech Co., Ltd. and acclimatized for 1 week prior to experimentation. An overview of the experimental design is provided in Figure 1B. The schematic diagram illustrates the experimental timeline, including burn induction, treatment regimen, and sample collection points. The thermal burn model was adapted from previously reported murine thermal injury models, with minor modifications [18,19]. On day 0, mice were anesthetized with isoflurane and the dorsal area was shaved. A standardized deep second-degree thermal burn wound (~2 cm in diameter) was created on the dorsum using a preheated Yan-5Q thermal burn device (Shanghai Yuyan Scientific Instrument Co., Ltd., Shanghai, China) set at 100 °C with a constant contact time of 5 s to generate a uniform burn area. After burn induction, mice were randomly assigned to four groups (n = 11 per group): (i) untreated control, (ii) vehicle (white petrolatum), (iii) PIC (PIC in white petrolatum), and (iv) RES (RES in white petrolatum; positive control). From day 1 onward, the assigned ointment formulation (vehicle, PIC, or RES) was topically applied once daily to fully cover the wound bed under brief isoflurane anesthesia, whereas mice in the untreated control group received no topical treatment. Wound healing was monitored by daily digital photography, and wound area was quantified using NIH ImageJ 1.54f software. Body weight was recorded on days 0, 7, and 14. For histopathological evaluation, one mouse per group was randomly selected on days 3, 5, and 7, deeply anesthetized with isoflurane, and euthanized by cervical dislocation, followed by wound tissue collection. On day 8, mice were randomly allocated for downstream analyses: one mouse was used for a 5-ethynyl-2′-deoxyuridine (EdU) incorporation assay, one for Masson’s trichrome-based collagen analysis, and three for transcriptomic analysis. These animals were euthanized in the same manner, and wound samples were harvested accordingly. On day 14, all remaining mice were euthanized, and wound tissues and blood were collected for endpoint assessments.

### 2.4. Histological Staining and EdU-Based Proliferation Analysis

Fixed wound tissues were embedded in paraffin, sectioned, and stained with hematoxylin and eosin (H&E) or Masson’s trichrome following standard protocols. For assessment of cell proliferation, mice received an intraperitoneal injection of 5-ethynyl-2′-deoxyuridine (EdU; 20 mg/kg) 4 h before tissue collection. EdU incorporation was detected on tissue sections using a Click-iT EdU imaging kit according to the manufacturer’s instructions, followed by counterstaining with Hoechst 33342.

### 2.5. Transcriptomic Analysis

Total RNA was extracted from frozen skin tissues collected on day 8 post-burn (n = 3 per group) using TRIzol reagent according to the manufacturer’s instructions. RNA integrity and purity were assessed prior to library preparation. Sequencing libraries were constructed using standard protocols and subjected to high-throughput sequencing on the Illumina platform. Raw sequencing reads were processed for quality control, and clean reads were aligned to the reference genome. Gene expression levels were quantified based on mapped reads. Differentially expressed genes (DEGs) were identified using the criteria of |log_2_ fold change| ≥ 1 and *p* < 0.05. Functional enrichment analyses of DEGs were subsequently performed using Gene Ontology (GO) and Kyoto Encyclopedia of Genes and Genomes (KEGG) pathway databases [20,21].

### 2.6. Cell Culture

The human keratinocyte (HaCaT), human skin fibroblast (HSF), human umbilical vein endothelial cell (HUVEC), and murine macrophage (RAW 264.7) cell lines were obtained from Cyagen Biosciences (Guangzhou, China). Cells were cultured in RPMI 1640 medium supplemented with 10% heat-inactivated fetal bovine serum, 110 mg·L^−1^ sodium pyruvate, 100 U·mL^−1^ penicillin, and 100 μg·mL^−1^ streptomycin. All cultures were maintained at 37 °C in a humidified atmosphere containing 5% CO_2_ and 95% air. Prior to experimental treatments, cells were allowed to acclimate for 24 h under standard culture conditions. Specific details regarding drug treatments and experimental procedures are described in the corresponding figure legends.

### 2.7. Cell Viability Assay

Cell viability was assessed using the Cell Counting Kit-8 (CCK-8) assay. Briefly, cells were seeded into 96-well plates and treated with PIC at the indicated concentrations. After the designated incubation period, 10 μL of CCK-8 reagent (Beyotime, Shanghai, China; Cat. No. C0039) was added to each well, and the plates were further incubated at 37 °C for 2 h. Absorbance was measured at 450 nm using a microplate reader. Cell viability was calculated using the following formula: (OD_treatment/OD_control) × 100%.

### 2.8. Scratch Wound Healing Assay

Cells were seeded into 6-well plates and cultured until a confluent monolayer was established. A uniform linear scratch was generated across the cell monolayer using a sterile pipette tip to simulate a wound. To minimize the influence of cell proliferation, the culture medium was replaced with serum-free medium, and cells were treated with the indicated concentrations of test compounds. Cell migration into the scratched area was monitored and imaged at 0 and 24 h post-scratch using a microscope equipped with a 10× objective. The wound area was quantified using ImageJ software, first importing the captured images of the scratch at 0 h and the experimental time point and calibrating the scale bar for physical unit conversion. Then trace the cell-free wound area using the software’s drawing tools to measure the area values of the two time points separately, and the migration rate was calculated accordingly: $Wound\;Closure\;(\%)=\left[\frac{A_{t=0h}-A_{t=△h}}{A_{t=0h}}\right]$, where $A_{t=0h}$ is the initial wound area and $A_{t=△h}$ is the wound area at the measured time point [20]. Cell seeding density for the in vitro scratch wound assay should be empirically determined according to the specific cell type and the size of the culture vessel or well, with the critical requirement of forming a confluent and uniform cell monolayer prior to the creation of the scratch [20,21].

For the HaCaT–HSF co-culture scratch assay, HaCaT keratinocytes and HSFs were seeded at a 1:1 ratio to establish a simplified epithelial–mesenchymal interaction model. This ratio was selected to allow both keratinocyte- and fibroblast-derived responses to contribute to scratch closure and fibrosis-related marker expression, while avoiding dominance of either cell type in the co-culture system. The model was used to assess inflammation-driven epithelial–mesenchymal crosstalk and scar-associated fibroblast activation rather than keratinocyte migration alone.

### 2.9. Tube Formation Assay

Matrigel (Beyotime, Shanghai, China, Cat. No. C3072) was diluted with Endothelial Cell Medium (ScienCell, Carlsbad, CA, USA, Cat. No. 1001) at a ratio of 2:1 and thoroughly mixed. Subsequently, 50 µL of the diluted Matrigel was evenly dispensed into each well of a 96-well plate and allowed to solidify at 37 °C. HUVECs were serum-starved for 24 h, harvested, and resuspended at a density of 1 × 10^5^ cells/mL. A 50 µL aliquot of the cell suspension was added to each Matrigel-coated well and incubated for 6 h. For treatment groups, cells were resuspended in complete medium containing the test compound at the designated working concentration before seeding onto the Matrigel-coated wells following the same procedure. Once optimal capillary-like network formation was observed, the culture medium was carefully removed. The cells were stained with Calcein AM live-cell dye and visualized using a fluorescence microscope [22].

### 2.10. Intracellular ROS Assay

Intracellular reactive oxygen species (ROS) levels were detected using a Reactive Oxygen Species Assay Kit (Beyotime, Shanghai, China, Cat. No. S0033M). HaCaT cells were adjusted to a density of 1 × 10^5^ cells/mL and seeded into chamber slides at 400 μL per well. After 24 h of culture, cells in the treatment groups were incubated with PIC at the indicated concentrations for 24 h. Subsequently, LPS was added at a final concentration of 10 μg/mL, and the cells were further stimulated for 12 h. After treatment, the ROS-sensitive fluorescent probe was loaded into the cells and incubated for 20 min at 37 °C. The probe solution was then removed, and the cells were gently washed three times with PBS. The slides were immediately examined under a confocal microscope (STELLARIS LAS X, Leica Microsystems, Mannheim, Germany). Images were acquired using the FITC channel (excitation 488 nm, emission 525 nm) to visualize ROS-associated fluorescence, and changes in intracellular ROS levels were quantitatively analyzed.

### 2.11. Western Blotting

Total proteins were extracted from cultured cells using RIPA lysis buffer (Beyotime, Shanghai, China, Cat. No. P0013B) supplemented with protease and phosphatase inhibitor cocktails (Beyotime, Shanghai, China, Cat. No. P1005; Biosharp, Anhui, China, Cat. No. BL615A). Protein samples were separated by 10% SDS-PAGE and subsequently transferred onto PVDF membranes. The membranes were blocked with 5% non-fat milk for 1 h at room temperature and then incubated overnight at 4 °C with specific primary antibodies. After washing, the membranes were incubated with horseradish peroxidase-conjugated secondary IgG antibodies (Multi Sciences, Hangzhou, China). Protein bands were visualized using enhanced chemiluminescence [23] reagents (SparkJade, Jinan, China, Cat. No. ED0016) [24,25,26].

### 2.12. Statistical Analysis

Statistical evaluation was carried out using GraphPad Prism 8 software (GraphPad Software, San Diego, CA, USA). Data normality was assessed with the Shapiro–Wilk test. Normally distributed data were presented as mean ± standard deviation (SD), unless specified otherwise. Comparisons between two groups were performed using an unpaired two-tailed Student’s *t*-test [13]. Differences with *p*-values below 0.05 were considered statistically significant.

## 3. Results

### 3.1. PIC Accelerates Burn Wound Healing

To evaluate the therapeutic efficacy of PIC (Figure 1A) in acute cutaneous burn repair, we established a standardized contact heat-induced burn model in C57BL/6 mice and topically administered vehicle (white petrolatum), PIC, or RES as a positive control (Figure 1B). Representative gross photographs collected from day 0 to day 14 clearly illustrate the dynamic progression of wound healing (Figure 1C). Quantitative planimetric analysis revealed that PIC significantly promoted wound contraction beginning on day 4, compared with the other groups, ultimately leading to near-complete closure by day 14 (Figure 1D,E). Throughout the experimental period, PIC was well tolerated, as indicated by stable body weight trajectories and normal serum biochemical parameters, collectively supporting an excellent in vivo safety profile, consistent with previous reports in other disease models (Figure 1F,G) [27]. Importantly, histological assessment using H&E staining further demonstrated that PIC-treated wounds exhibited markedly improved tissue remodeling by day 7, characterized by restoration of normal epidermal and dermal architecture, increased fibrous connective tissue formation, and enhanced neovascularization within the wound bed (Figure 1H).

### 3.2. PIC Enhances Wound Cell Proliferation and ECM Remodeling

**Figure 2:**
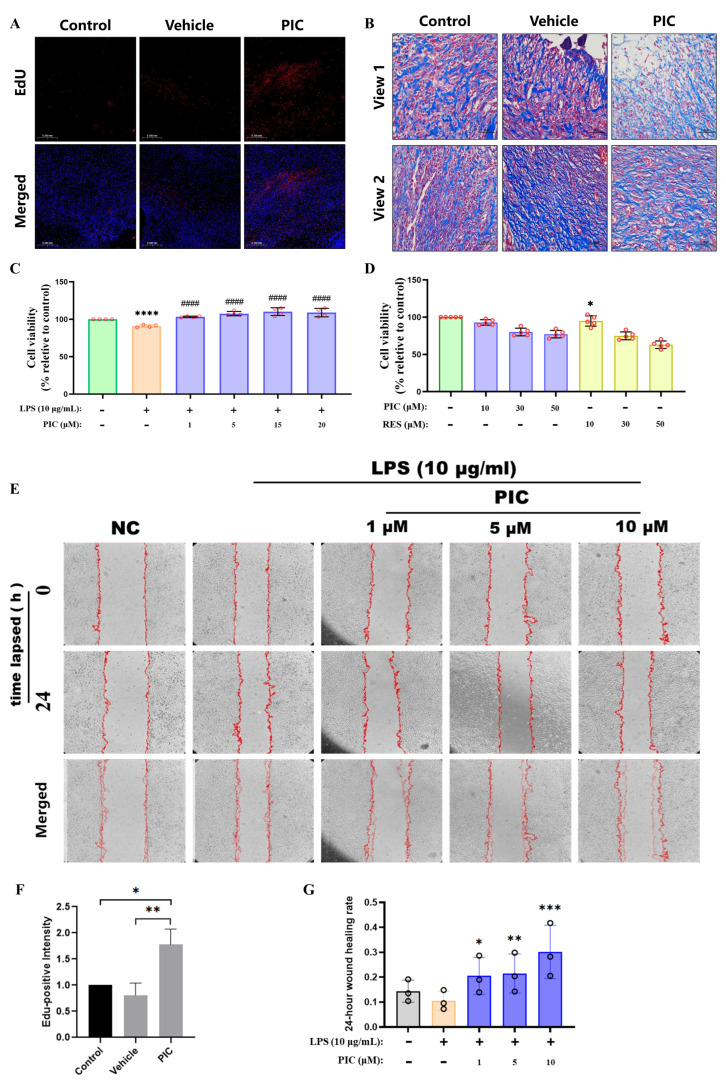
Piceatannol enhances cell proliferation and fibrogenesis during cutaneous burn wound repair. (**A**) EdU staining (red) of murine wound tissue on day 8 demonstrating proliferating cells; nuclei were counterstained with DAPI (blue). Scale bar, 50 μm. (**B**) Masson’s trichrome staining of murine wound tissue on day 8 illustrating collagen deposition (blue); the PIC-treated group exhibited denser and more orderly arranged collagen fibers. Scale bar, 100 μm. (**C**) Effects of PIC on the viability of HaCaT keratinocytes. Cells were seeded and allowed to adhere for 24 h, followed by LPS stimulation for 6 h and treatment with PIC or resveratrol at the indicated concentrations for another 18 h. CCK-8 reagent was then added, and absorbance was measured after 2 h of incubation. (**D**) Effects of PIC and resveratrol on the viability of human skin fibroblasts (HSFs). Cells were seeded and allowed to adhere for 24 h, followed by treatment with PIC or resveratrol at the indicated concentrations for another 24 h before CCK-8 assay. (**E**) Representative images of the scratch wound-healing (cell migration) assay. (**F**) Quantitative analysis of EdU-positive cells in (**A**). (**G**) Quantitative analysis of scratch closure in (**E**). Data are presented as mean ± SD. * *p* < 0.05, ** *p* < 0.01, *** *p* < 0.001, **** *p* < 0.0001 vs. control; #### *p* < 0.001 vs. LPS group.

Wound healing is not a single event but a highly orchestrated and dynamic biological process, consisting of overlapping phases that collectively restore the skin barrier and tissue integrity. It involves precisely coordinated interactions among multiple cell types, signaling molecules, and the extracellular matrix (ECM) [28]. To further assess the proliferative activity of cells within the wound bed, we performed a 5-ethynyl-2′-deoxyuridine (EdU) incorporation assay. The results demonstrated markedly increased EdU-positive cells in PIC-treated wounds, providing direct evidence of enhanced DNA synthesis and a greater number of actively proliferating cells (Figure 2A,F).

In parallel with cellular proliferation, effective wound repair requires extensive ECM remodeling. The synthesis, deposition, and maturation of collagen fibers are central to this reconstructive phase, as their structural organization critically determines both the tensile strength of regenerated tissue and the risk of abnormal scar formation [29]. As shown in Figure 2B, Masson’s trichrome staining confirmed substantial collagen deposition in the dermis across all experimental groups. Notably, the PIC-treated group exhibited a significantly higher density of collagen fibers with superior architectural organization, characterized by uniformly thin, well-aligned, and interwoven collagen bundles. In contrast, although the Vaseline group also displayed collagen formation, the fibers were less organized and showed areas of irregular thickening and discontinuity.

### 3.3. PIC Preserves HaCaT Cell Viability and Promotes Migration Under Inflammatory Conditions

Keratinocytes play a central role in the early response to skin injury [30]. Following tissue damage, immune cell recruitment initiates the inflammatory phase, which in turn creates a microenvironment that regulates subsequent healing events [31]. The proliferative phase is essential for tissue regeneration, during which keratinocytes at the wound margin proliferate from the basal layer, differentiate, and migrate across the provisional matrix to re-establish the epidermal barrier and achieve wound closure [32]. Based on our in vivo observations that PIC markedly enhanced cell proliferation at the wound edge, we next investigated its direct biological effects in vitro.

We therefore examined the influence of PIC on HaCaT keratinocyte viability and migration under inflammatory conditions. As shown in Figure 2C, the CCK-8 assay demonstrated that 1 μM PIC significantly protected HaCaT cells from LPS-induced injury, as reflected by increased relative cell viability. Higher concentrations of PIC further improved cell viability in a concentration-dependent manner.

Fibroblasts represent another key cell population involved in tissue development, homeostasis, and repair processes. Following injury, they become major contributors to ECM synthesis and tissue regeneration [33]. As presented in Figure 2D, PIC treatment did not directly stimulate the proliferation of human skin fibroblasts (HSFs). Although no severe cytotoxicity was observed within the tested concentration range, cell viability tended to decrease at higher concentrations of PIC and RES. Therefore, the subsequent mechanistic experiments were performed using concentrations that maintained acceptable cell viability, allowing the observed effects on fibrotic markers to be interpreted independently of overt cytotoxicity.

To further evaluate the effect of PIC on keratinocyte motility, a scratch wound-healing assay was performed. After generating uniform linear scratches in confluent HaCaT monolayers, cells were exposed to 10 μg/mL LPS in the presence or absence of different concentrations of PIC. Quantitative analysis demonstrated that PIC treatment markedly promoted cell migration and accelerated scratch closure, whereas LPS exposure alone significantly impaired migratory capacity (Figure 2E,G). These findings collectively indicate that PIC effectively counteracts inflammation-induced dysfunction of keratinocytes and facilitates re-epithelialization.

### 3.4. Transcriptomic Analysis Reveals the Molecular Basis by Which PIC Promotes Burn Wound Healing

**Figure 3:**
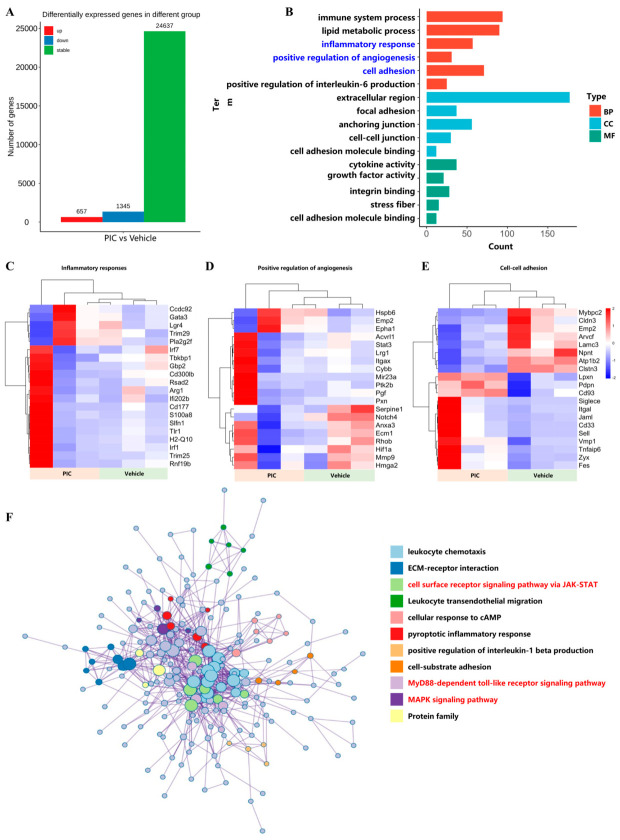
Transcriptomic profiling reveals the molecular effects of piceatannol on burn wound healing in mice. (**A**) Bar plot summarizing differentially expressed genes (DEGs) in PIC-treated wounds compared with the vehicle group (n = 3; |fold change| ≥ 2; *p* < 0.05). (**B**) Gene Ontology (GO) enrichment analysis of DEGs in the PIC group versus the vehicle group. (**C**–**E**) Heatmaps showing representative DEGs associated with inflammatory response, angiogenesis, and cell adhesion pathways. Each column represents an individual biological replicate from the PIC or vehicle group, and colors indicate normalized relative expression levels. (**F**) Gene interaction network illustrating putative regulatory relationships among DEGs and enriched functional modules.

To explore the mechanisms underlying PIC-mediated burn wound repair, we collected wound-edge skin tissues from mice on day 8 after treatment for transcriptomic profiling (Figure 1B). Compared with the vehicle group, the PIC-treated group exhibited 657 upregulated genes and 1345 downregulated genes (Figure 3A). GO enrichment analysis of these DEGs indicated that PIC treatment primarily influenced biological processes related to immune system processes, lipid metabolic processes, inflammatory response, positive regulation of angiogenesis, cell adhesion, and focal adhesion (Figure 3B).

After tissue injury, damage-associated molecular patterns (DAMPs) released from damaged cells and disrupted extracellular matrix components, together with pathogen-associated molecular patterns (PAMPs) when microbial contamination is present, are recognized by pattern-recognition receptors and trigger innate immune activation [34,35]. These upstream danger signals can activate Toll-like receptor-, NF-κB-, and interferon-regulatory factor (IRF)-associated transcriptional programs, thereby inducing the expression of multiple inflammation-related genes. In this context, the altered expression of TLR1, IRF1, and TRIM25 observed in burn wounds likely reflects activation of injury-induced innate immune and interferon-associated inflammatory responses, whereas the upregulation of TRIM29 may represent a negative regulatory response that limits excessive inflammatory signaling. Consistent with this interpretation, our transcriptomic data suggest that PIC mitigates excessive inflammation in burn wounds, as reflected by the downregulation of pro-inflammatory mediators such as TLR1, IRF1, and TRIM25, together with the upregulation of the negative regulatory molecule TRIM29 (Figure 3C). In parallel, PIC enhanced the expression of angiogenesis-associated genes and modulated adhesion-related programs, both of which are tightly linked to tissue remodeling and scar-relevant matrix organization during later stages of wound healing (Figure 3D,E).

Moreover, gene interaction network analysis indicated that these pathways are functionally intertwined rather than acting in isolation, forming a highly connected regulatory network centered on key hub nodes (Figure 3F). Notably, NF-κB emerged as a potential signaling hub, integrating upstream cues such as AKT signaling to drive inflammatory responses, while also coordinating cell growth-associated transcriptional programs via SP1 (Figure 3F). SP1 is predicted to regulate staphylococcal nuclease domain-containing protein 1 (SND1; also known as Tudor-SN), a multifunctional regulator implicated in diverse biological processes ranging from gene expression control to cell growth regulation [36].

### 3.5. PIC Suppresses ROS-Associated Inflammatory Responses

**Figure 4:**
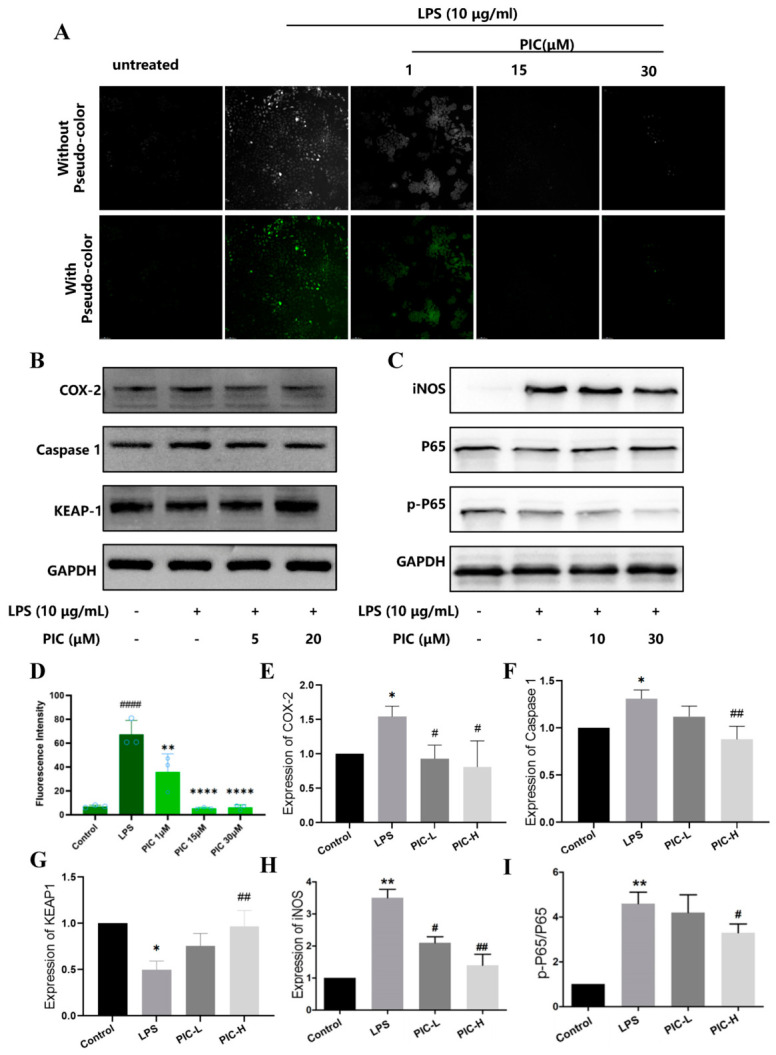
Piceatannol attenuates inflammation and oxidative stress during wound healing. (**A**) Representative fluorescence images showing intracellular ROS generation in HaCaT cells. (**B**) Immunoblot analysis of COX-2, caspase-1, and KEAP1 protein levels in HaCaT cells treated with PIC at the indicated concentrations. (**C**) Immunoblot analysis of iNOS, NF-κB p65, and phospho-p65 (p-p65) in PIC-treated RAW 264.7 macrophages. (**D**) Quantification of fluorescence intensity in (**A**). (**E**–**G**) Densitometric quantification of the immunoblot bands in (**B**), analyzed using ImageJ. (**H**,**I**) Densitometric quantification of the immunoblot bands in (**C**), analyzed using ImageJ. Data are presented as mean ± SD. * *p* < 0.05, ** *p* < 0.01, **** *p* < 0.0001 vs. control; # *p* < 0.05, ## *p* < 0.01, #### *p* < 0.0001 vs. LPS group. The original Western Blot Image is shown in the Supplementary Materials.

ROS are key redox signaling mediators involved in normal metabolism as well as human pathophysiology [37]. In burn injury, excessive ROS accumulation can amplify inflammation and promote cell death and fibrotic remodeling, thereby impairing wound repair and tissue regeneration [38]. In line with our transcriptomic data, we next examined whether PIC could alleviate oxidative stress under inflammatory conditions. HaCaT cells were pretreated with PIC for 24 h and subsequently challenged with LPS. Fluorescence-based ROS detection revealed that PIC significantly reduced intracellular ROS generation: 1 μM PIC decreased ROS levels, whereas 15 μM PIC nearly restored ROS to baseline, indicating a strong inhibitory effect on ROS production (Figure 4A,D).

Mechanistically, LPS activates HaCaT cells largely through TLR4/NF-κB signaling and can prime inflammasome activation, including assembly of the ROS-sensitive NLRP3 inflammasome, thereby promoting IL-1β maturation via caspase-1 activation [39]. Consistently, LPS stimulation markedly increased COX-2 and caspase-1 expression and decreased Kelch-like ECH-associated protein 1 (KEAP1) levels, suggesting concurrent activation of inflammatory signaling and disruption of oxidative stress regulation, which may contribute to a self-reinforcing loop between inflammation and oxidative stress [40]. Notably, PIC (20 μM) significantly reduced COX-2 and caspase-1 abundance in HaCaT cells and restored KEAP1 expression, supporting a dual inhibitory effect on inflammatory activation and oxidative stress (Figure 4B,E–G).

To further validate the anti-inflammatory activity of PIC, we established a parallel LPS-induced inflammatory model in murine macrophage RAW264.7 cells. PIC treatment (30 μM) markedly decreased iNOS expression and NF-κB activation, as indicated by reduced p-p65 levels, further confirming its potent anti-inflammatory efficacy (Figure 4C,H,I).

### 3.6. PIC Suppresses Scar-Related Responses During Burn Wound Healing

**Figure 5:**
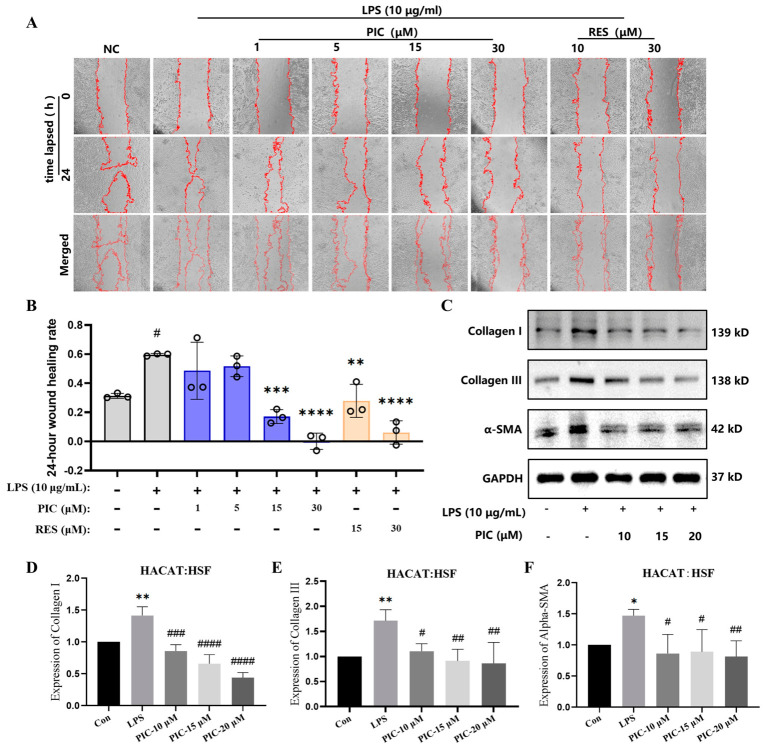
Piceatannol suppresses scar-related collagen production by inhibiting collagen I/III synthesis. (**A**) Representative images of wound closure in a 1:1 HaCaT–HSF co-culture treated with PIC. (**B**) Quantification of the migration (wound closure) rate in (**A**). (**C**) Immunoblot analysis of collagen I, collagen III, and α-SMA in co-cultured cells treated with PIC at the indicated concentrations. (**D**–**F**) Densitometric quantification of the immunoblot bands in (**C**), performed using ImageJ. Data are presented as mean ± SD. * *p* < 0.05, ** *p* < 0.01, *** *p* < 0.001, **** *p* < 0.0001 vs. control; # *p* < 0.05, ## *p* < 0.01, ### *p* < 0.001, #### *p* < 0.0001 vs. LPS group.

Post-burn wound repair frequently results in hypertrophic scar (HS) formation, which can substantially impair patients’ quality of life [41]. HS is generally regarded as a fibroproliferative disorder characterized by excessive and disorganized extracellular matrix (ECM) deposition at the wound site [42]. Building on our in vivo observations, we next explored whether PIC could mitigate scar-associated cellular responses in vitro. To this end, we established a 1:1 HaCaT–HSF co-culture model and assessed inflammation-driven changes in wound closure using a scratch assay. This co-culture model was chosen because burn wound remodeling involves reciprocal interactions between keratinocytes and fibroblasts, particularly during inflammation-driven matrix remodeling and scar formation. The 1:1 ratio was used to maintain comparable contributions from both cell types and to provide a simplified but balanced model of epithelial–mesenchymal crosstalk. The results showed that LPS stimulation markedly accelerated scratch closure, indicating an inflammation-induced pro-migratory/repair phenotype, whereas PIC treatment significantly blunted this effect and restored wound-closure dynamics toward basal levels (Figure 5A,B).

Although the mechanisms underlying HS remain incompletely understood, accumulating evidence indicates that persistent inflammation is a critical driver of fibrotic remodeling, promoting collagen synthesis and correlating with eventual scar severity [43]. Inflammatory signaling also cooperates with profibrotic pathways (notably TGF-β signaling) to promote fibroblast activation and differentiation into α-SMA-positive myofibroblasts, which produce large amounts of matrix proteins and contribute to collagen-rich scar formation [44]. Therefore, we further evaluated the effects of PIC on fibrotic markers in the co-culture system. As shown in Figure 5C–F, LPS robustly induced fibrosis-associated proteins, whereas PIC reversed these changes in a dose-dependent manner, significantly reducing the expression of collagen I, collagen III, and α-SMA, consistent with an inhibitory effect on scar-related collagen production.

### 3.7. PIC Enhances Angiogenesis Through Activation of the STAT3–VEGF Axis

**Figure 6:**
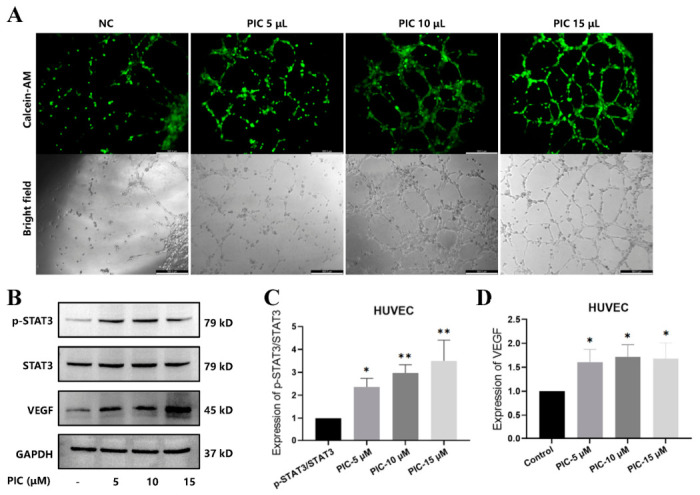
Piceatannol promotes angiogenic activity and modulates STAT3/VEGF signaling in vitro. (**A**) Representative images of capillary-like tube formation by HUVECs seeded on Matrigel and treated with PIC (0, 5, 10, and 15 μM). Scale bar, 200 μm. (**B**) Immunoblot analysis of phospho-STAT3 (p-STAT3), total STAT3, and VEGF in HUVECs treated with PIC at the indicated concentrations; GAPDH served as the loading control. (**C**,**D**) Densitometric quantification of the immunoblot bands in (**B**), performed using ImageJ. Data are presented as mean ± SD. * *p* < 0.05, ** *p* < 0.01 vs. control.

Angiogenesis is a critical component of wound repair, as it supports granulation tissue formation and provides oxygen and nutrients required for tissue regeneration [45]. To assess the pro-angiogenic potential of PIC, we evaluated its effects on capillary-like tube formation by HUVECs on Matrigel. As shown in Figure 6A, PIC promoted tube network formation in a concentration-dependent manner, with higher doses producing more extensive and better-connected tubular structures, indicating robust pro-angiogenic activity.

To investigate the underlying mechanism, we examined the STAT3/VEGF pathway in HUVECs. Immunoblotting revealed that PIC increased STAT3 phosphorylation and upregulated VEGF protein expression in a dose-dependent manner (Figure 6B–D), suggesting activation of the STAT3–VEGF signaling axis. Activated phospho-STAT3 is known to translocate into the nucleus and enhance VEGF transcription, thereby elevating VEGF expression and promoting angiogenic behaviors such as endothelial proliferation, migration, and tube formation [46]. Collectively, these data indicate that PIC enhances endothelial angiogenic capacity, at least in part, through STAT3-dependent upregulation of VEGF.

## 4. Discussion

The present study provides experimental evidence that PIC, a bioactive stilbene constituent of *P. cuspidatum* (Huzhang), promotes burn wound repair. *P. cuspidatum* is a traditional Chinese medicinal plant historically used for trauma-, blood stasis-, and inflammation-related disorders, supporting the pharmacological relevance of its active constituents in tissue injury and repair. In a murine deep second-degree burn model, topical PIC accelerated wound closure, improved tissue architecture, enhanced re-epithelialization and neovascularization, and promoted more organized collagen remodeling. These in vivo effects were further supported by mechanistic findings in keratinocytes, macrophages, endothelial cells, fibroblasts, and keratinocyte–fibroblast co-cultures, suggesting that PIC acts on multiple interconnected processes required for effective burn wound healing. It should be noted that although PIC was originally identified as a naturally occurring stilbene present in *P. cuspidatum* and several other plant species, it can now be readily synthesized with high purity and consistent quality. The availability of synthetic PIC further enhances its translational and pharmaceutical potential by facilitating scalable production and quality control.

The present findings are also consistent with a growing body of evidence indicating that natural phenolic compounds can facilitate wound repair through coordinated regulation of multiple biological processes. For example, resveratrol [47,48], curcumin [49,50], quercetin [51,52], and epigallocatechin gallate [53,54] have been reported to promote wound healing by attenuating excessive inflammatory responses, reducing oxidative stress, modulating fibroblast activation and extracellular matrix remodeling, and supporting angiogenesis. These overlapping mechanisms are highly relevant to burn wound repair, in which inflammation, redox imbalance, vascular regeneration, and collagen remodeling are closely interconnected. Therefore, the multi-process regulatory profile observed for PIC is not unique to this compound alone, but is consistent with the broader pharmacological behavior of phenolic compounds in complex tissue-injury settings.

A major finding of this study is that PIC attenuated inflammation–oxidative stress crosstalk, a central pathological feature of burn injury. Burn wounds are characterized by excessive inflammatory activation and ROS accumulation, which can impair keratinocyte migration, endothelial function, and matrix remodeling. Transcriptomic analysis showed that PIC modulated immune- and inflammation-related gene programs while also affecting angiogenesis, cell adhesion, and extracellular matrix-associated pathways. In LPS-treated keratinocytes, PIC reduced ROS accumulation, decreased COX-2 and caspase-1 expression, and restored KEAP1 protein levels. In macrophages, PIC suppressed iNOS expression and NF-κB/p65 activation. These results suggest that PIC limits the amplification of inflammatory and oxidative injury, thereby creating a microenvironment more favorable for tissue repair. The transcriptomic changes in TLR1, IRF1, TRIM25, and TRIM29 should therefore be interpreted in this injury-triggered inflammatory context. Burn-induced tissue damage can activate innate immune signaling through DAMP/PAMP–pattern-recognition receptor pathways, leading to NF-κB- and IRF-associated transcriptional activation. Thus, the downregulation of TLR1, IRF1, and TRIM25 by PIC likely reflects attenuation of damage-induced innate immune activation, whereas the upregulation of TRIM29 may indicate enhancement of negative regulatory or anti-inflammatory feedback. These findings support the view that PIC modulates an injury-induced inflammatory gene program, rather than directly targeting each individual gene. The modulation of KEAP1/NRF2-associated redox homeostasis may contribute to the therapeutic action of PIC. In the present study, PIC restored KEAP1 protein levels under inflammatory stress, whereas total NRF2 abundance was not markedly increased. This observation suggests that PIC may regulate KEAP1/NRF2-related redox signaling through changes in pathway tone, protein stability, intracellular localization, or downstream transcriptional activity rather than simple upregulation of total NRF2 expression. Therefore, this pathway should be interpreted cautiously. Future studies should examine NRF2 nuclear translocation, antioxidant response genes such as HO-1, NQO1, and GCLC, and KEAP1 turnover dynamics to clarify the causal role of this signaling module in PIC-mediated burn repair.

PIC also supported epithelial restitution under inflammatory stress. Re-epithelialization is essential for restoring the skin barrier and limiting secondary inflammatory stimulation. In HaCaT keratinocytes, PIC improved cell viability under LPS challenge and promoted scratch closure, indicating that it can counteract inflammation-associated impairment of keratinocyte function. This effect is consistent with the improved epidermal restoration observed in vivo and suggests that early suppression of inflammatory and oxidative stress may facilitate the transition from the inflammatory phase to the proliferative phase of wound healing.

Another important aspect of PIC action is its regulation of fibrotic remodeling. Hypertrophic scarring after burns is closely associated with persistent inflammation, fibroblast activation, myofibroblast differentiation, and excessive or disorganized collagen deposition. At first glance, the inhibitory effects of PIC on LPS-driven scratch closure and collagen I, collagen III, and α-SMA expression in the HaCaT–HSF co-culture system may appear inconsistent with both the accelerated wound closure observed in vivo and the enhanced scratch closure observed in LPS-stimulated HaCaT monocultures. However, these findings reflect different biological contexts.

In HaCaT monoculture, scratch closure primarily reflects keratinocyte migration and epithelial restitution. Therefore, the ability of PIC to accelerate scratch closure under LPS stimulation suggests protection against inflammation-induced impairment of keratinocyte motility. In contrast, the HaCaT–HSF co-culture model captures epithelial–mesenchymal interactions and inflammation-driven fibroblast activation, processes that are closely linked to fibrotic remodeling and scar formation. In this setting, LPS-induced rapid closure may represent an exaggerated repair/fibrotic phenotype rather than beneficial re-epithelialization alone. Consequently, PIC may promote keratinocyte-mediated epithelial repair while simultaneously restraining excessive fibroblast activation and myofibroblast-associated matrix production.

Similarly, the apparent discrepancy between the in vivo and co-culture findings can be explained by the different biological endpoints measured. In the burn model, wound closure represents an integrated outcome of inflammation resolution, re-epithelialization, angiogenesis, granulation tissue formation, and extracellular matrix remodeling. By contrast, the LPS-stimulated co-culture model primarily reflects inflammation-driven scar-associated responses. Therefore, PIC may accelerate gross wound closure by improving the quality and coordination of repair in vivo while suppressing excessive inflammation-driven fibrotic activation in vitro. These findings suggest that PIC promotes balanced collagen remodeling rather than simply maximizing collagen production, which may contribute to high-quality repair and potentially reduce pathological scarring. However, the current co-culture model cannot fully reproduce the mechanical tension, immune complexity, and chronic remodeling environment involved in hypertrophic scar formation in vivo. Longer-term scar-related studies are therefore warranted to determine whether PIC can reduce pathological scarring after burn injury.

Angiogenesis is another key process improved by PIC. Adequate vascular regeneration is required for oxygen and nutrient delivery, granulation tissue formation, and high-quality repair. Transcriptomic data suggested enrichment of angiogenesis-associated programs in PIC-treated wounds, and functional assays showed that PIC enhanced HUVEC tube formation. Mechanistically, PIC increased STAT3 phosphorylation and VEGF expression, supporting activation of the STAT3–VEGF axis. These results indicate that PIC may directly promote endothelial angiogenic capacity. Nevertheless, increased angiogenic signaling or vessel-like structure formation does not necessarily prove functional vascular regeneration in vivo. Future studies should assess vascular maturity and perfusion using approaches such as CD31/α-SMA co-staining, perfusion assays, and hypoxia-related markers.

**Figure 7:**
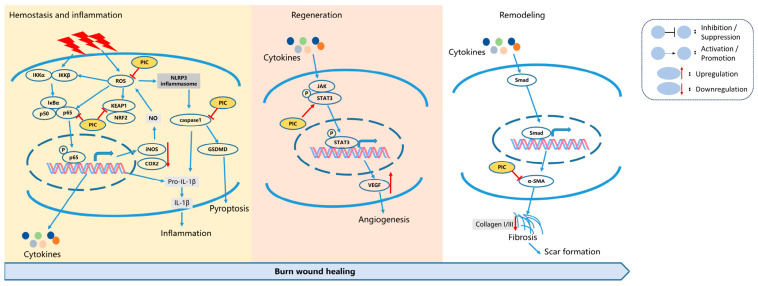
Schematic summary of the proposed mechanisms underlying PIC-mediated burn wound repair.

Taken together, these findings support a working model in which PIC promotes burn wound healing by coordinately regulating inflammation–oxidative stress crosstalk, angiogenesis, epithelial repair, and fibrotic remodeling (Figure 7). The multiple biological effects observed in this study should not be interpreted as several unrelated actions occurring independently. Rather, they may arise from the coordinated regulation of interconnected stress-response and repair pathways. In burn wounds, inflammation, oxidative stress, angiogenesis, epithelial restitution, and fibrotic remodeling are tightly coupled processes. Excessive ROS can amplify NF-κB activation and inflammasome-related signaling, whereas persistent inflammation can further promote fibroblast activation, myofibroblast differentiation, and excessive collagen deposition. Conversely, resolution of inflammation and normalization of redox homeostasis may create a more favorable microenvironment for keratinocyte migration, angiogenesis, and balanced extracellular matrix remodeling. In this context, PIC appears to act as a pleiotropic but network-modulating phenolic compound, with suppression of ROS-associated inflammatory signaling, particularly involving NF-κB and KEAP1/NRF2-associated redox regulation, serving as an important upstream event. The observed effects on epithelial restitution, STAT3–VEGF-associated angiogenic activity, and fibroblast-to-myofibroblast transition may therefore represent downstream or parallel consequences within the wound-healing network rather than fully independent mechanisms. This interpretation is consistent with previous studies on other phenolic compounds, including resveratrol, curcumin, quercetin, and epigallocatechin gallate, which have been reported to regulate wound repair through overlapping mechanisms involving anti-inflammatory, antioxidant, pro-angiogenic, and matrix-remodeling effects. Thus, the pleiotropic activity of PIC is not unexpected, but reflects a broader pharmacological feature of natural phenolics in complex tissue-repair settings.

Several limitations should be acknowledged. First, although PIC consistently regulated NF-κB, KEAP1/NRF2-associated redox responses, and STAT3–VEGF signaling, causal pathway validation using pharmacological inhibitors, gene silencing, or genetic models remains necessary. Second, functional vascular regeneration was not directly assessed in vivo. Third, the current study mainly focused on early-to-intermediate wound repair, whereas long-term scar outcomes and biomechanical properties require further evaluation. Finally, optimized topical formulations and local skin exposure studies are needed to clarify whether the effective concentrations observed experimentally can be translated into clinically relevant burn wound therapy.

Overall, this study demonstrates that PIC, a bioactive stilbene constituent of *P. cuspidatum*, promotes burn wound repair through coordinated regulation of inflammation–oxidative stress crosstalk, epithelial restitution, angiogenesis, and fibrotic remodeling. These findings support the therapeutic potential of *P. cuspidatum*-derived natural products in skin repair and identify PIC as a promising candidate for further development as a topical treatment for burn wounds.

## 5. Conclusions

PIC, a bioactive stilbene constituent of *P. cuspidatum* (Huzhang), promoted burn wound repair as a multifunctional topical agent by modulating inflammation–oxidative stress crosstalk, epithelial restitution, angiogenesis, and fibrotic remodeling. By targeting multiple interconnected processes rather than a single pathway, PIC may better match the biological complexity of burn wound healing. These findings provide pharmacological support for the therapeutic potential of *P. cuspidatum*-derived natural products in skin repair and identify PIC as a promising candidate for topical burn wound management. Further studies involving causal pathway validation, functional vascular assessment, long-term scar evaluation, local skin exposure analysis, and optimized topical formulations are warranted to clarify its translational potential.

## 6. Patents

A patent application related to this work has been filed in China (Application No. 202510049811.9).

## Data Availability

The data presented in this study are available on request from the corresponding author.

## Supplementary Materials

The following supporting information can be downloaded at: https://www.mdpi.com/article/10.3390/biom16070926/s1, Original Western Blots.

## Abbreviations

The following abbreviations are used in this manuscript:

CCK-8	cell counting kit-8
DEGs	differentially expressed genes
ECM	extracellular matrix
EdU	5-ethynyl-2′-deoxyuridine
GO	Gene Ontology
H&E	hematoxylin and eosin
HS	hypertrophic scar
HSF	human skin fibroblast
HUVEC	human umbilical vein endothelial cell
KEAP1	Kelch-like ECH-associated protein 1
KEGG	Kyoto Encyclopedia of Genes and Genomes
LPS	lipopolysaccharide
NF-κB	nuclear factor kappa B
PIC	piceatannol
RES	resveratrol
ROS	reactive oxygen species
STAT3	signal transducer and activator of transcription 3
VEGF	vascular endothelial growth factor
