# Supplementary figures and images for: Piceatannol Promotes Burn Wound Healing by Coordinately Modulating Inflammation–Oxidative Stress Crosstalk, Angiogenesis, and Fibrotic Remodeling (part 2 of 2)
Source: Biomolecules. 2026 Jun 23;16(7):926. doi: 10.3390/biom16070926 (PMC13406660; doi:10.3390/biom16070926)

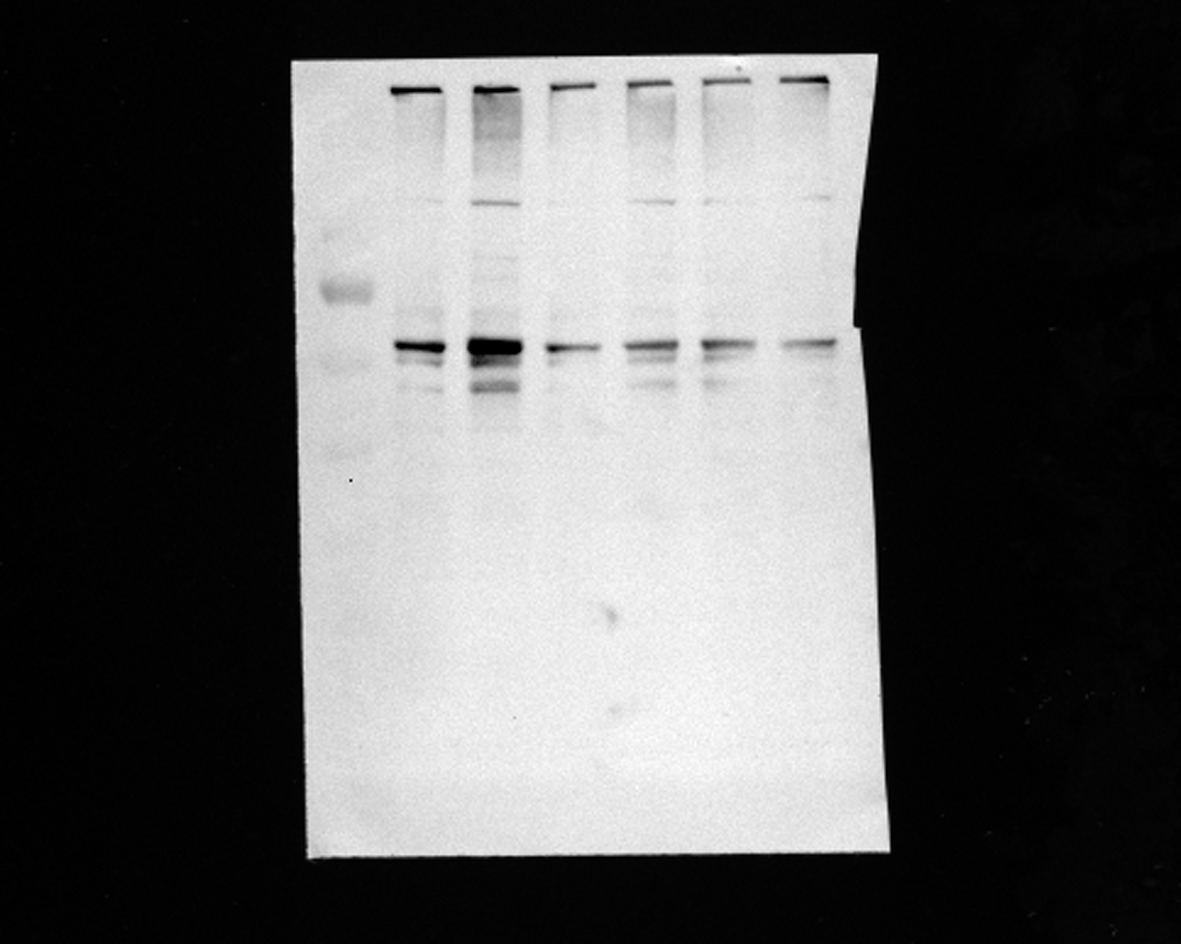

Supplement: Supplementary file 1 [file biomolecules-16-00926-s001.zip › biomolecules-4345458-WB/WB/scar formation/Alpha-SMA/membrane 1/A-SMA1.tif]

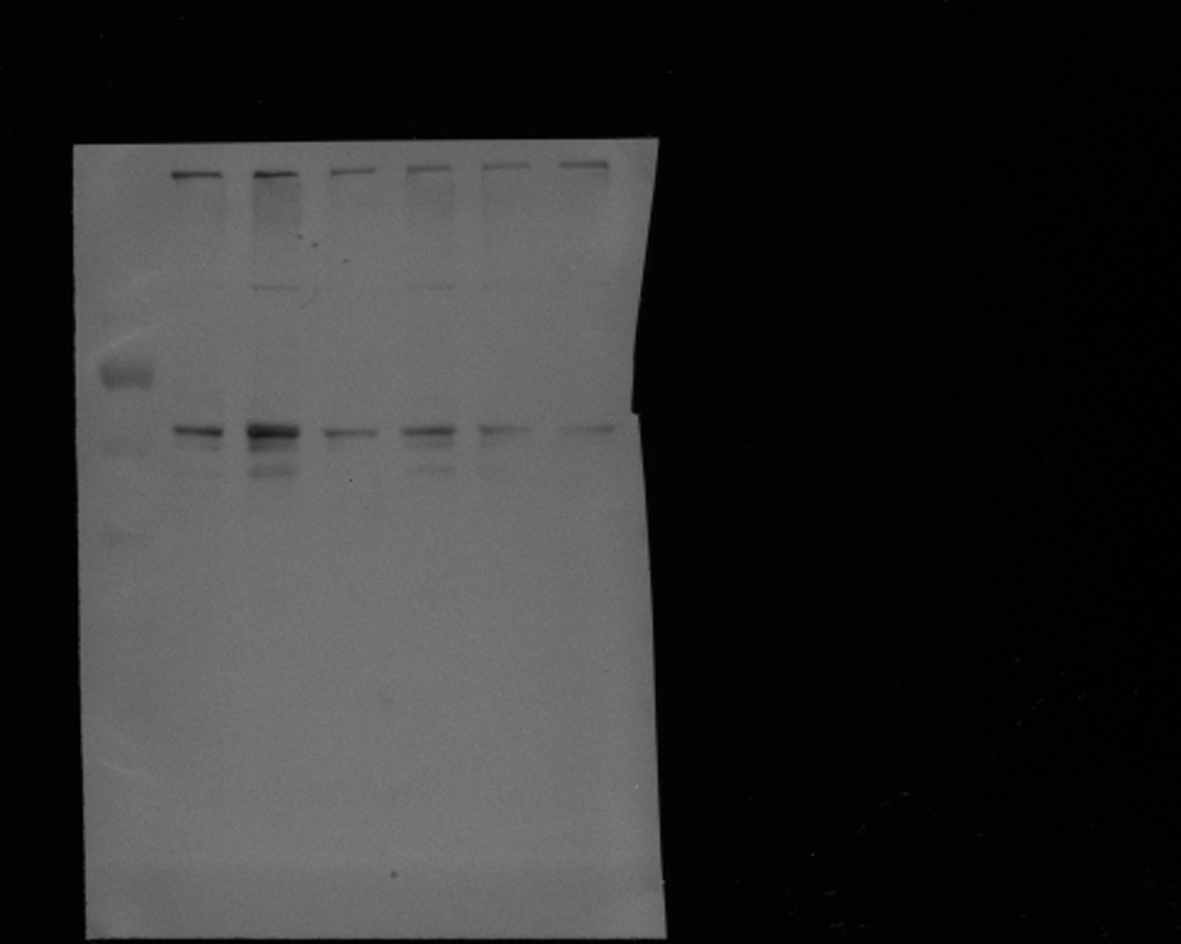

Supplement: Supplementary file 1 [file biomolecules-16-00926-s001.zip › biomolecules-4345458-WB/WB/scar formation/Alpha-SMA/membrane 1/A_SMA2.tif]

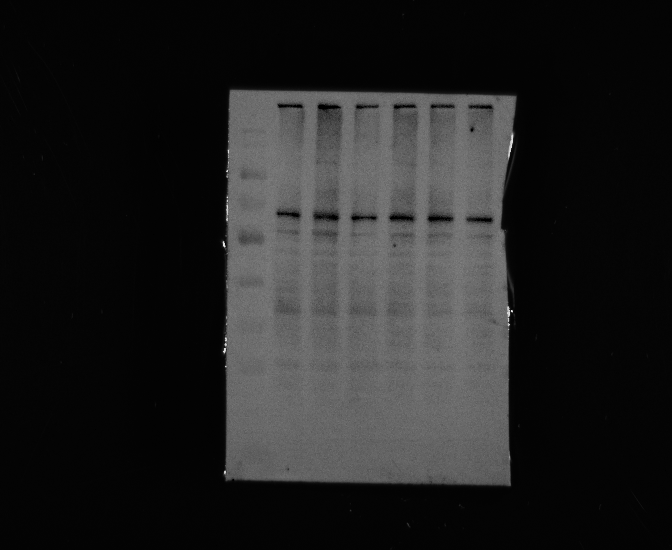

Supplement: Supplementary file 1 [file biomolecules-16-00926-s001.zip › biomolecules-4345458-WB/WB/scar formation/Alpha-SMA/membrane 1/Bactin.tif]

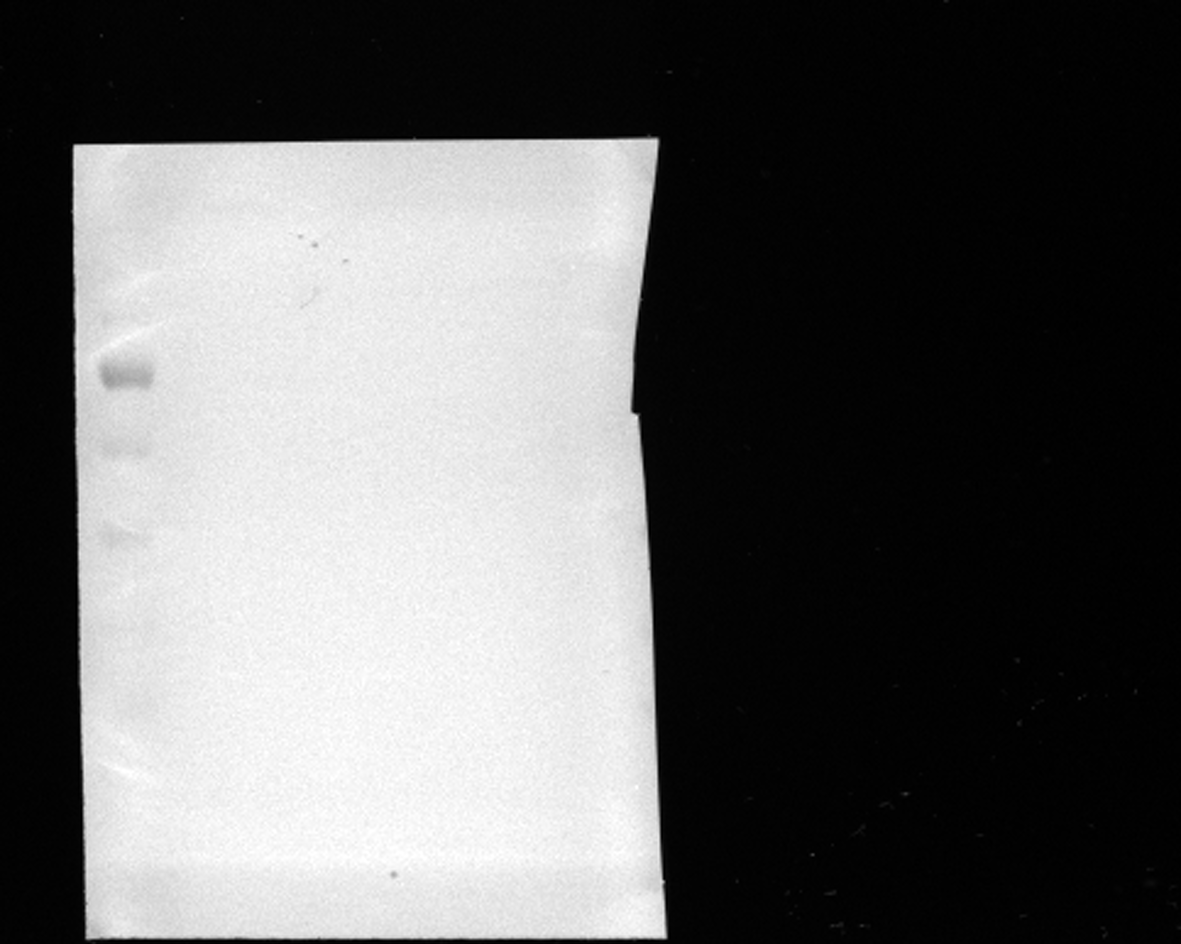

Supplement: Supplementary file 1 [file biomolecules-16-00926-s001.zip › biomolecules-4345458-WB/WB/scar formation/Alpha-SMA/membrane 1/BF.tif]

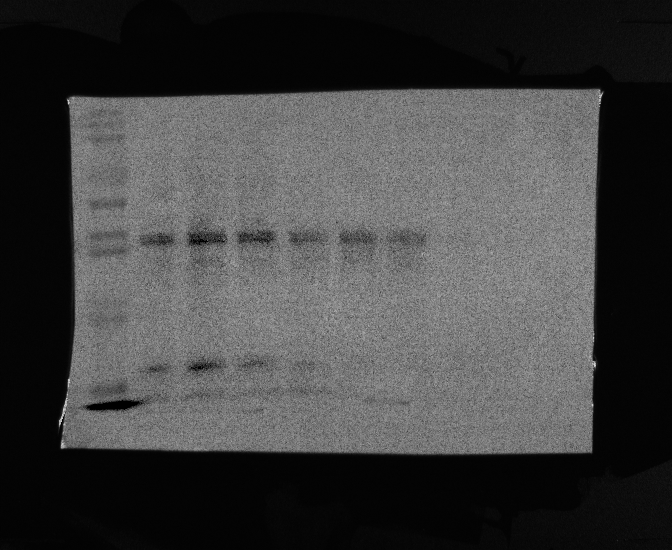

Supplement: Supplementary file 1 [file biomolecules-16-00926-s001.zip › biomolecules-4345458-WB/WB/scar formation/Alpha-SMA/membrane 2/1_8bit.tif]

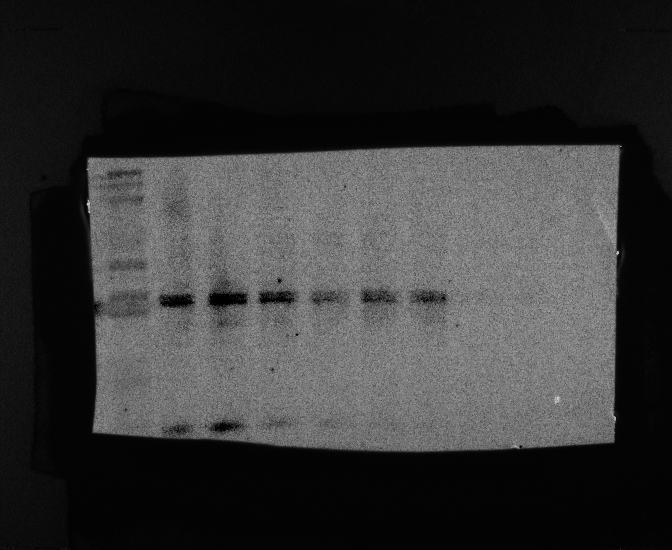

Supplement: Supplementary file 1 [file biomolecules-16-00926-s001.zip › biomolecules-4345458-WB/WB/scar formation/Alpha-SMA/membrane 2/2/1_8bit.tif]

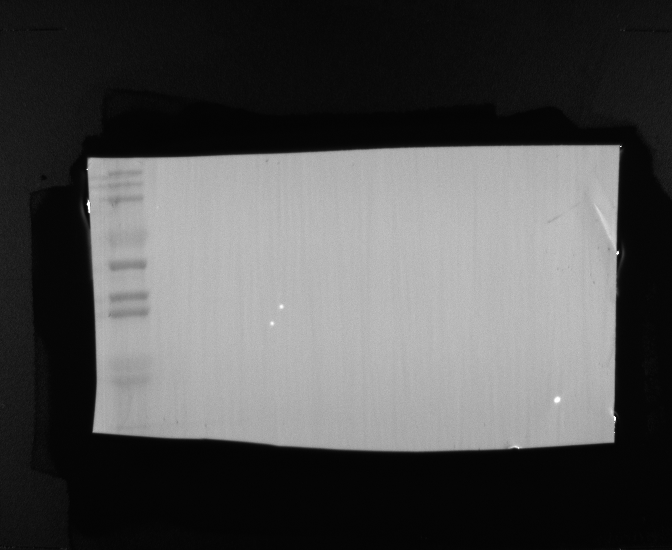

Supplement: Supplementary file 1 [file biomolecules-16-00926-s001.zip › biomolecules-4345458-WB/WB/scar formation/Alpha-SMA/membrane 2/2/bf_8bit.tif]

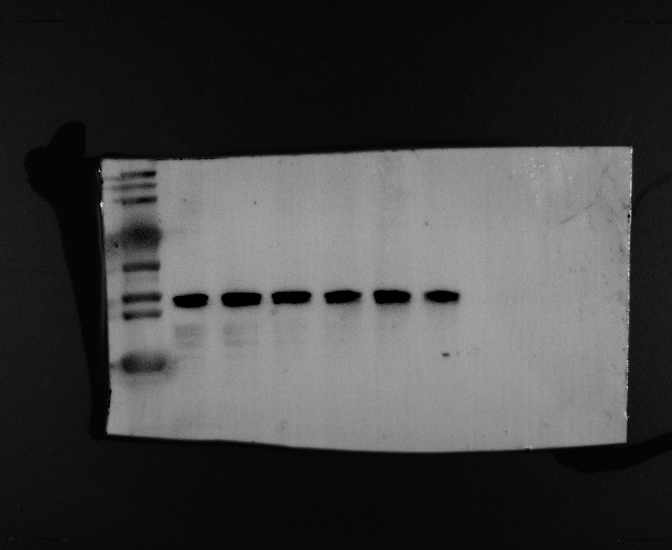

Supplement: Supplementary file 1 [file biomolecules-16-00926-s001.zip › biomolecules-4345458-WB/WB/scar formation/Alpha-SMA/membrane 2/2/Gapdh/1_8bit.tif]

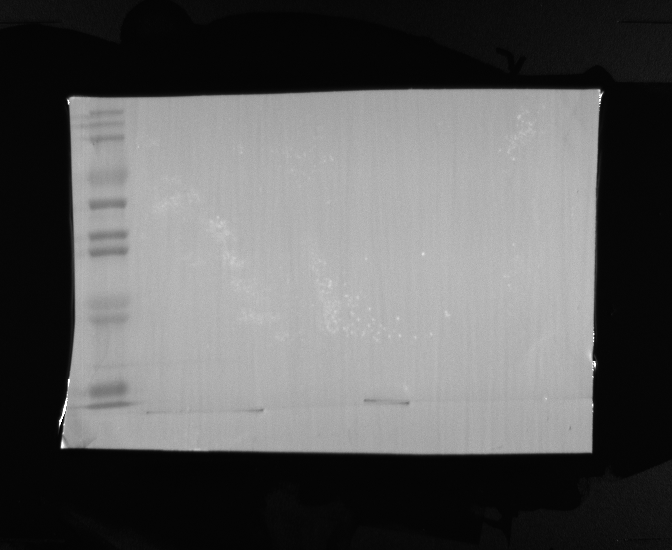

Supplement: Supplementary file 1 [file biomolecules-16-00926-s001.zip › biomolecules-4345458-WB/WB/scar formation/Alpha-SMA/membrane 2/BF_8bit.tif]

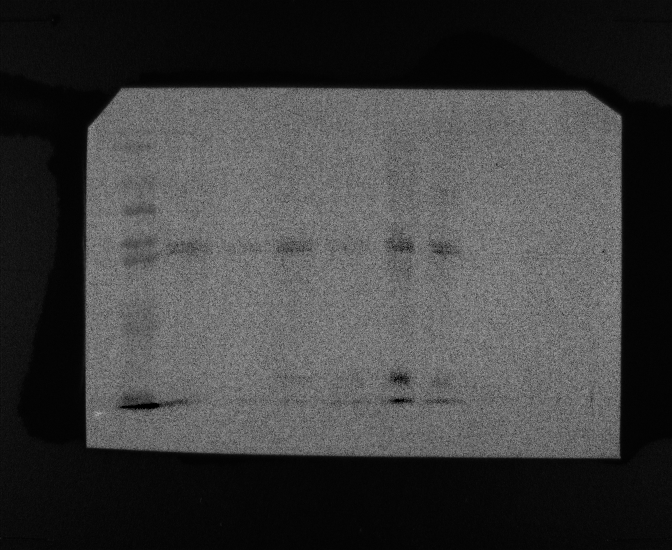

Supplement: Supplementary file 1 [file biomolecules-16-00926-s001.zip › biomolecules-4345458-WB/WB/scar formation/Alpha-SMA/membrane 3/1_8bit.tif]

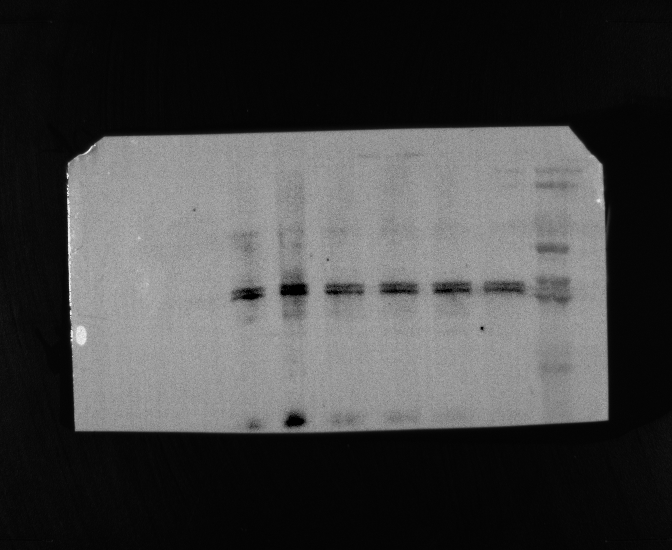

Supplement: Supplementary file 1 [file biomolecules-16-00926-s001.zip › biomolecules-4345458-WB/WB/scar formation/Alpha-SMA/membrane 3/2_8bit.tif]

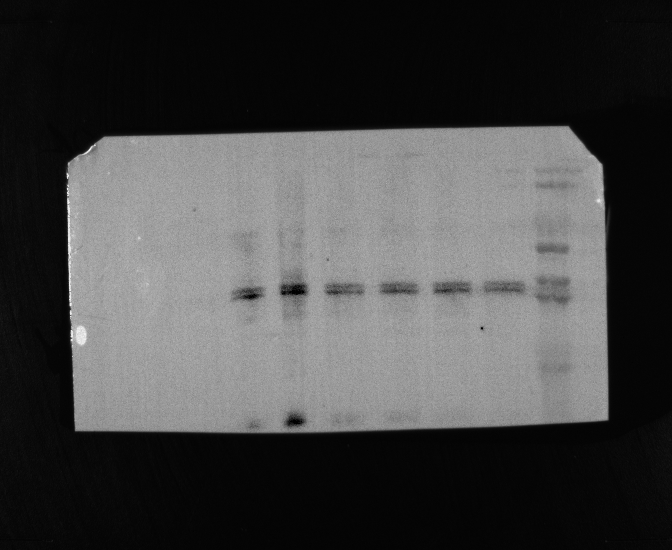

Supplement: Supplementary file 1 [file biomolecules-16-00926-s001.zip › biomolecules-4345458-WB/WB/scar formation/Alpha-SMA/membrane 3/3_8bit.tif]

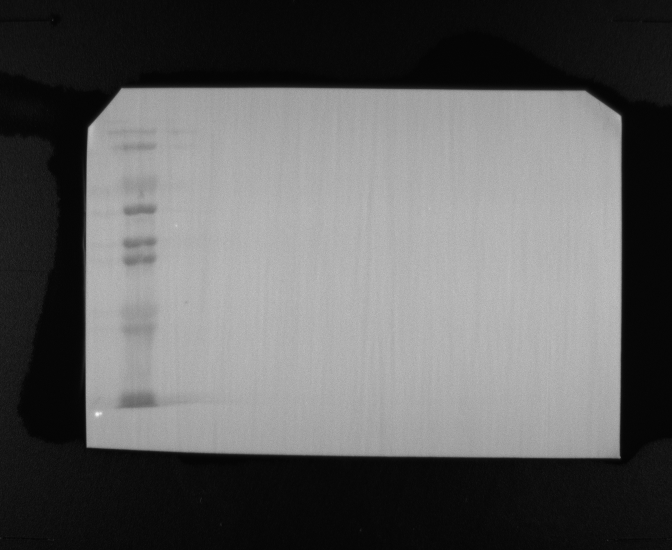

Supplement: Supplementary file 1 [file biomolecules-16-00926-s001.zip › biomolecules-4345458-WB/WB/scar formation/Alpha-SMA/membrane 3/BF_8bit.tif]

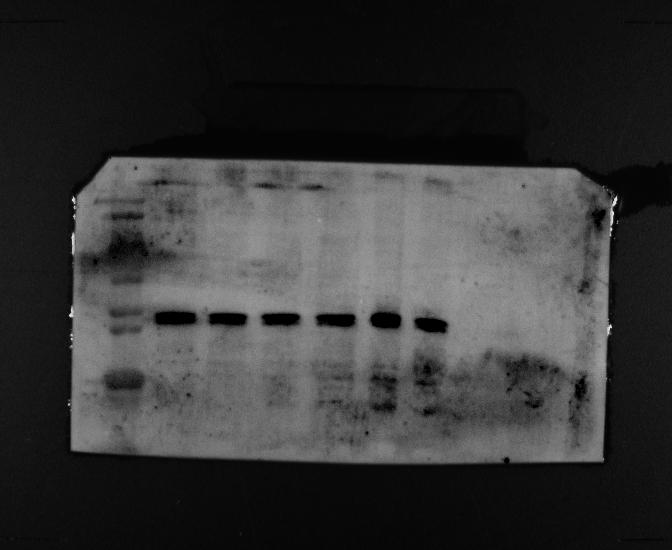

Supplement: Supplementary file 1 [file biomolecules-16-00926-s001.zip › biomolecules-4345458-WB/WB/scar formation/Alpha-SMA/membrane 3/gapdh/WB_20250903_171132_00.04_8bit.tif]

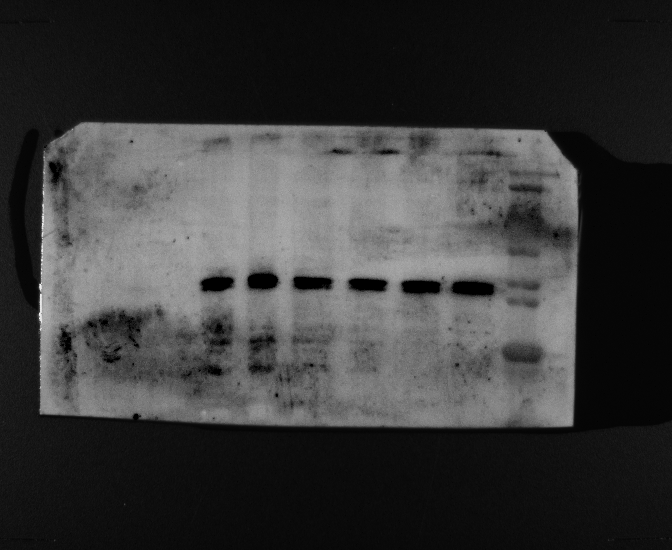

Supplement: Supplementary file 1 [file biomolecules-16-00926-s001.zip › biomolecules-4345458-WB/WB/scar formation/Alpha-SMA/membrane 3/gapdh/WB_20250903_171955_00.05_8bit.tif]

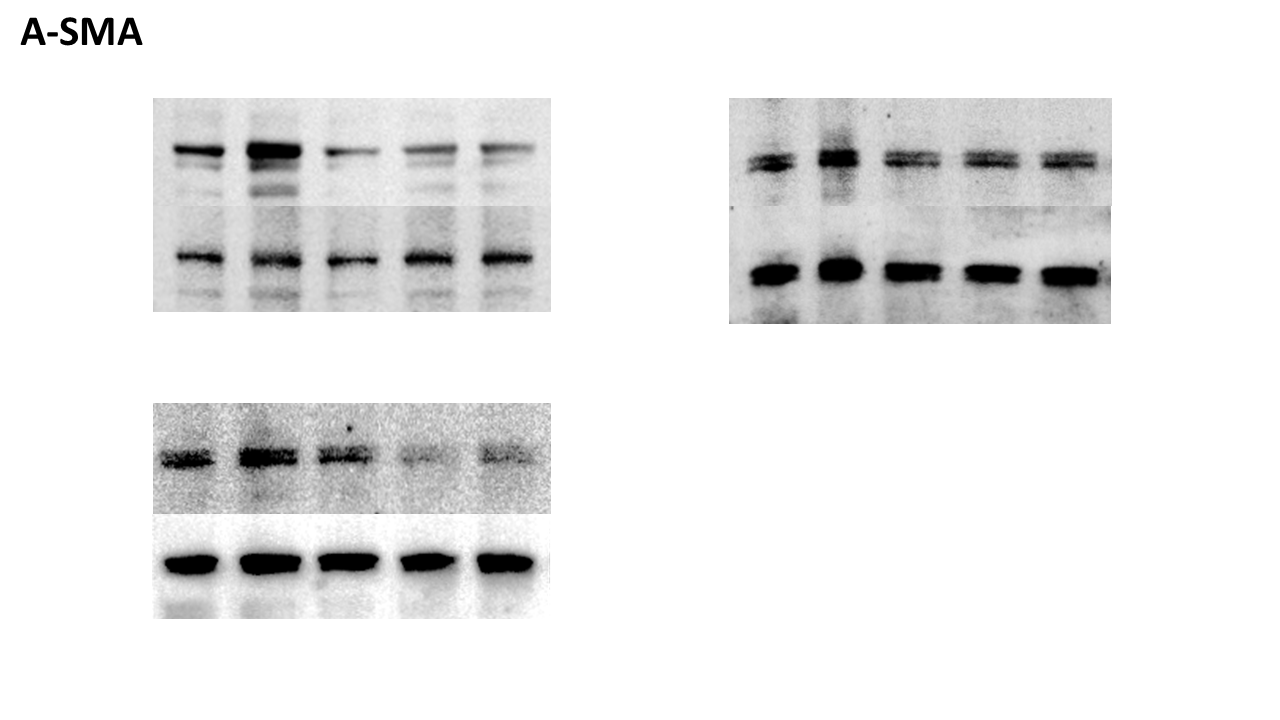

Supplement: Supplementary file 1 [file biomolecules-16-00926-s001.zip › biomolecules-4345458-WB/WB/scar formation/Alpha-SMA/WB.tif]

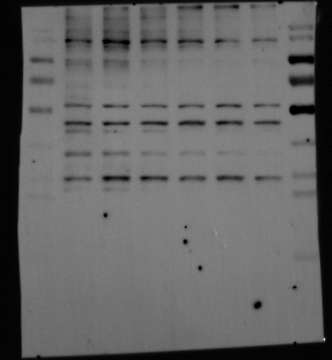

Supplement: Supplementary file 1 [file biomolecules-16-00926-s001.zip › biomolecules-4345458-WB/WB/scar formation/Collagen I/membrane 1/COLA1-1.tif]

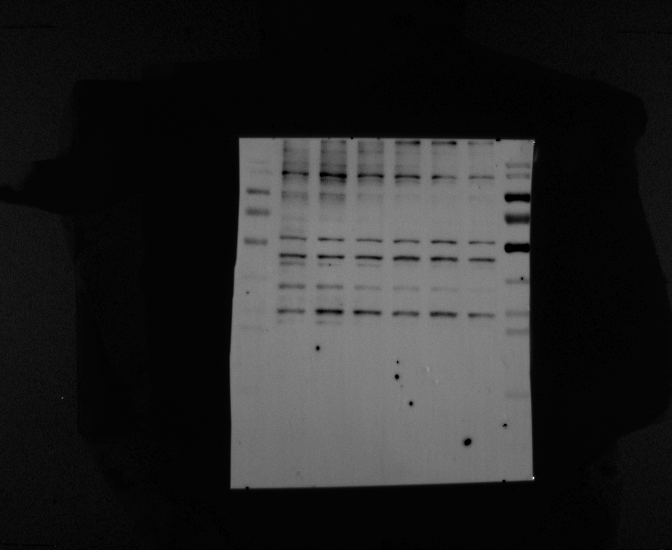

Supplement: Supplementary file 1 [file biomolecules-16-00926-s001.zip › biomolecules-4345458-WB/WB/scar formation/Collagen I/membrane 1/COLA1.tif]

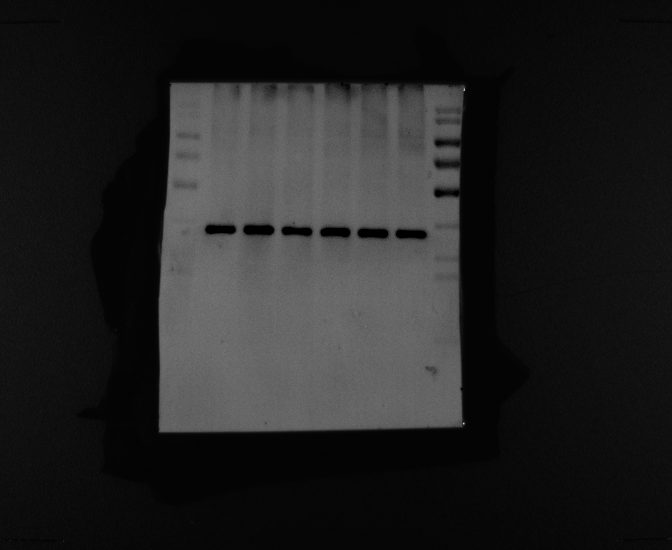

Supplement: Supplementary file 1 [file biomolecules-16-00926-s001.zip › biomolecules-4345458-WB/WB/scar formation/Collagen I/membrane 1/GAPDH_8bit.tif]

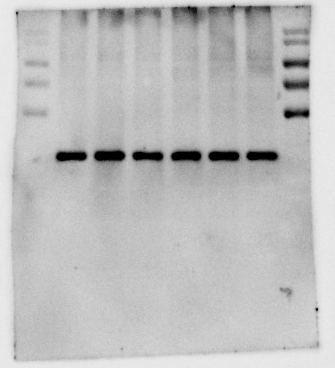

Supplement: Supplementary file 1 [file biomolecules-16-00926-s001.zip › biomolecules-4345458-WB/WB/scar formation/Collagen I/membrane 1/GAPDH_8bit_8bit-1.tif]

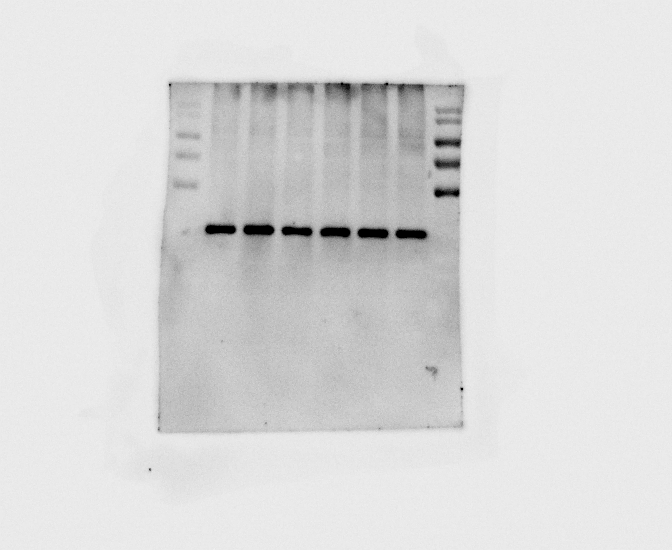

Supplement: Supplementary file 1 [file biomolecules-16-00926-s001.zip › biomolecules-4345458-WB/WB/scar formation/Collagen I/membrane 1/GAPDH_8bit_8bit.tif]

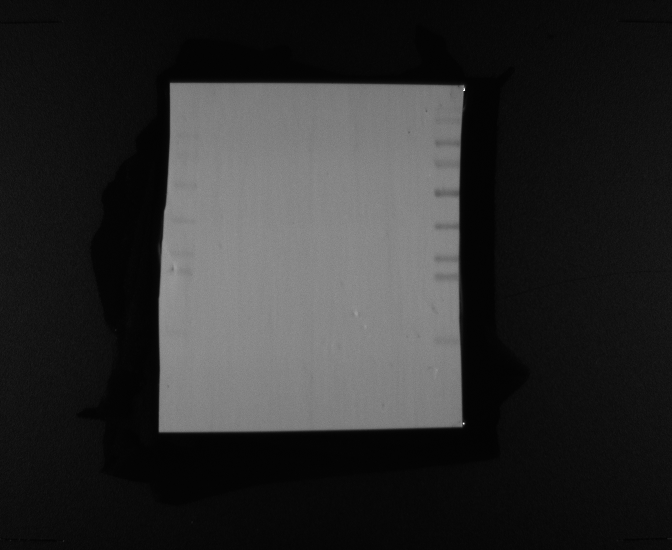

Supplement: Supplementary file 1 [file biomolecules-16-00926-s001.zip › biomolecules-4345458-WB/WB/scar formation/Collagen I/membrane 1/GAPDH_white-light_8bit.tif]

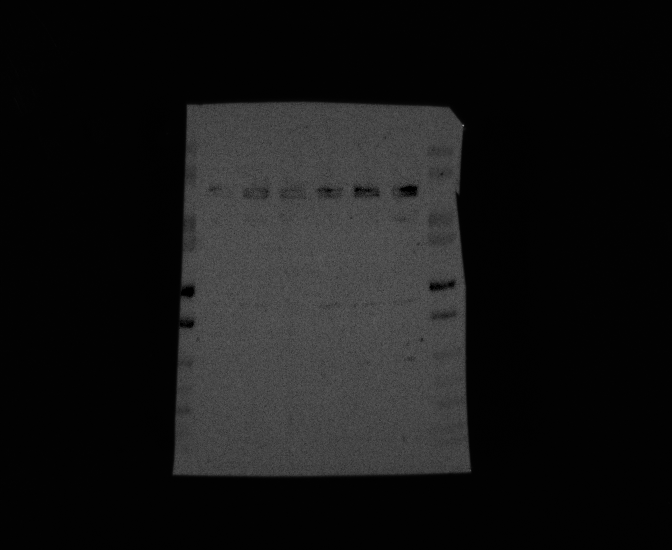

Supplement: Supplementary file 1 [file biomolecules-16-00926-s001.zip › biomolecules-4345458-WB/WB/scar formation/Collagen I/membrane 2/Collagen_I_1_30_8bit_8bit.tif]

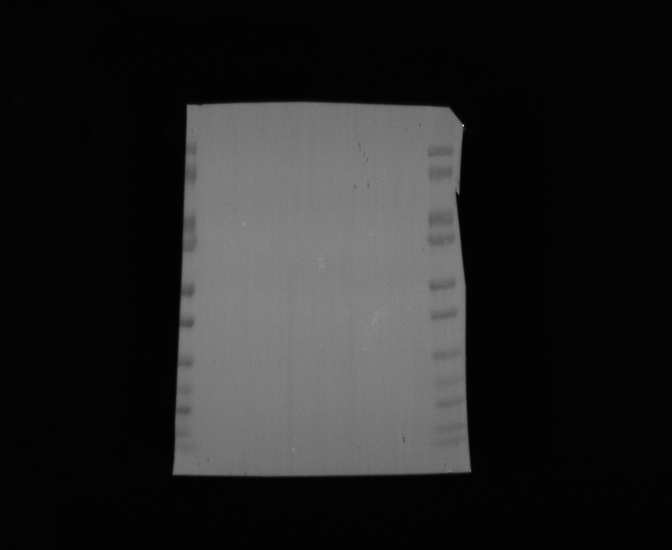

Supplement: Supplementary file 1 [file biomolecules-16-00926-s001.zip › biomolecules-4345458-WB/WB/scar formation/Collagen I/membrane 2/Collagen_I_bright filed_8bit.tif]

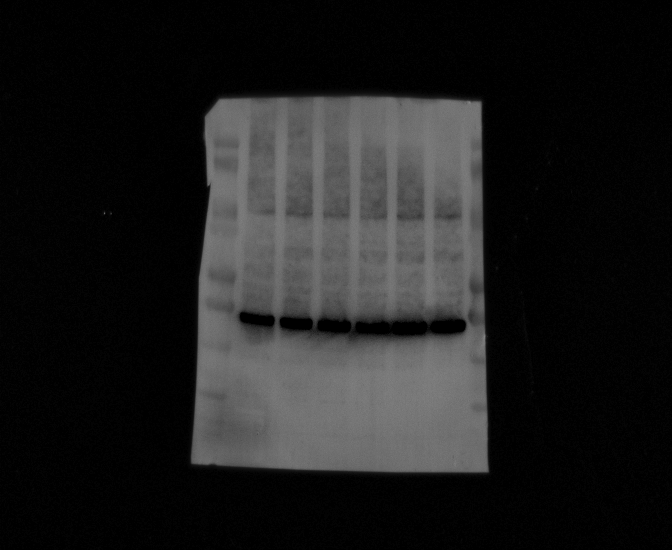

Supplement: Supplementary file 1 [file biomolecules-16-00926-s001.zip › biomolecules-4345458-WB/WB/scar formation/Collagen I/membrane 2/GAPDH/WB_20250410_165120_00.07.600-2_8bit.tif]

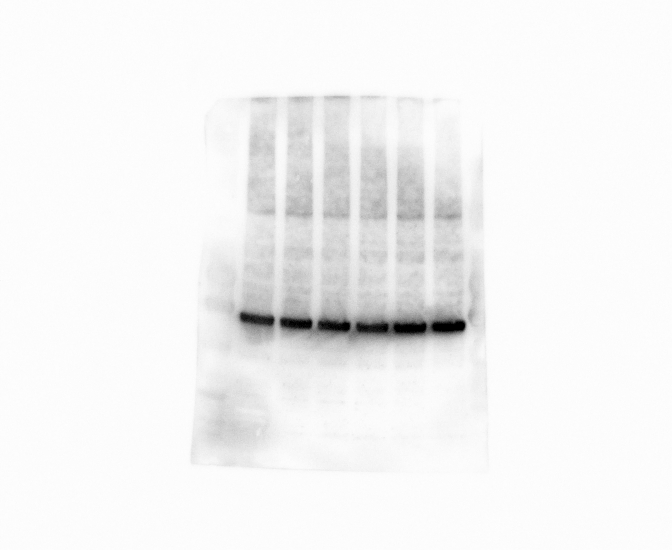

Supplement: Supplementary file 1 [file biomolecules-16-00926-s001.zip › biomolecules-4345458-WB/WB/scar formation/Collagen I/membrane 2/GAPDH/WB_20250410_165120_00.07.600-4_8bit.tif]

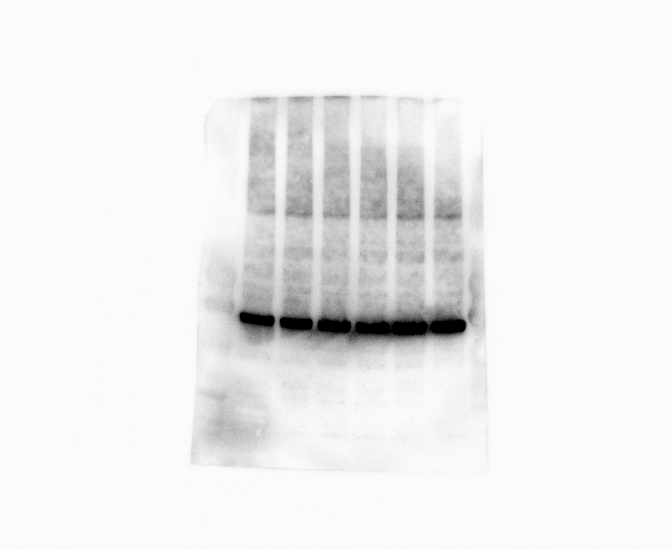

Supplement: Supplementary file 1 [file biomolecules-16-00926-s001.zip › biomolecules-4345458-WB/WB/scar formation/Collagen I/membrane 2/GAPDH/WB_20250410_165120_00.07_8bit(0).tif]

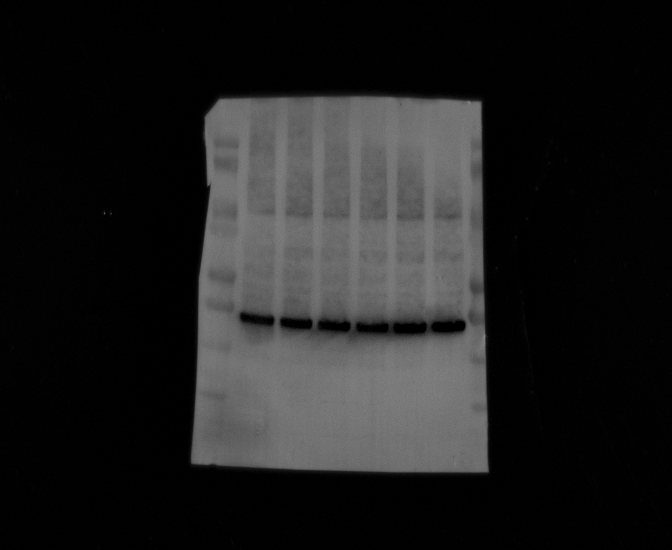

Supplement: Supplementary file 1 [file biomolecules-16-00926-s001.zip › biomolecules-4345458-WB/WB/scar formation/Collagen I/membrane 2/GAPDH/WB_20250410_165120_00.07_8bit.tif]

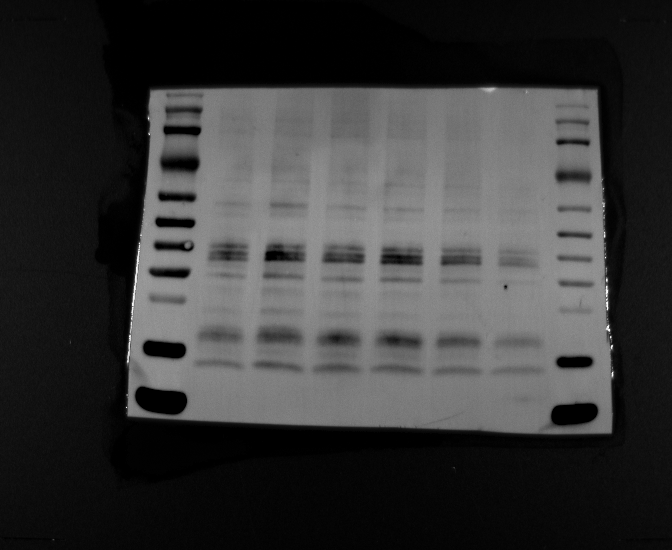

Supplement: Supplementary file 1 [file biomolecules-16-00926-s001.zip › biomolecules-4345458-WB/WB/scar formation/Collagen I/membrane 3/1_8bit.tif]

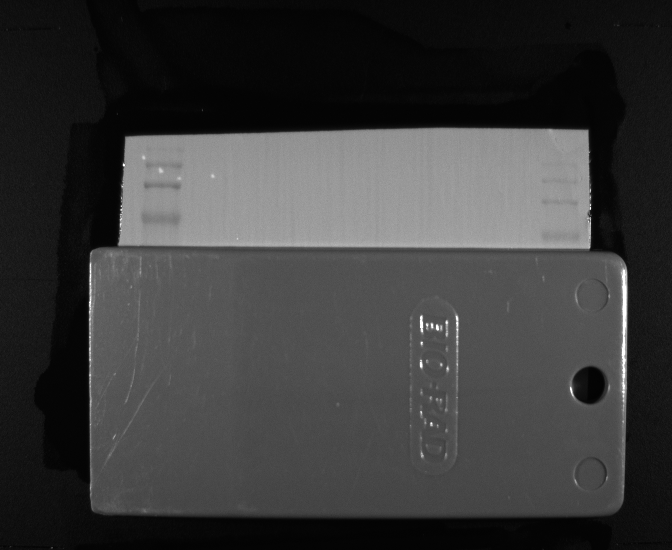

Supplement: Supplementary file 1 [file biomolecules-16-00926-s001.zip › biomolecules-4345458-WB/WB/scar formation/Collagen I/membrane 3/bf-1_8bit.tif]

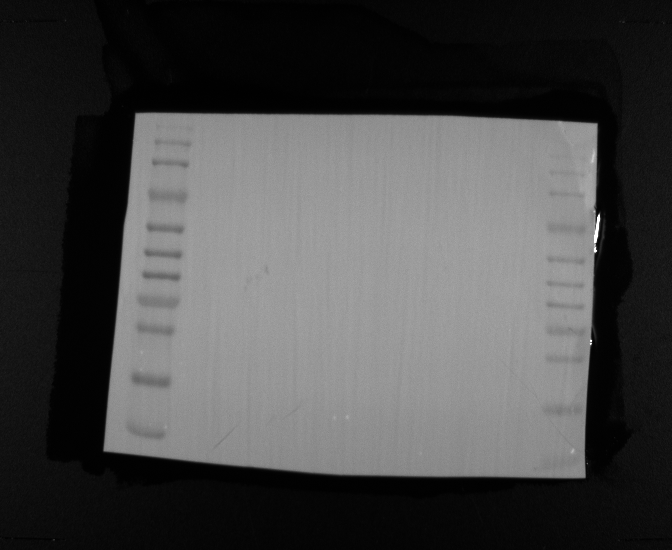

Supplement: Supplementary file 1 [file biomolecules-16-00926-s001.zip › biomolecules-4345458-WB/WB/scar formation/Collagen I/membrane 3/bf-2_8bit.tif]

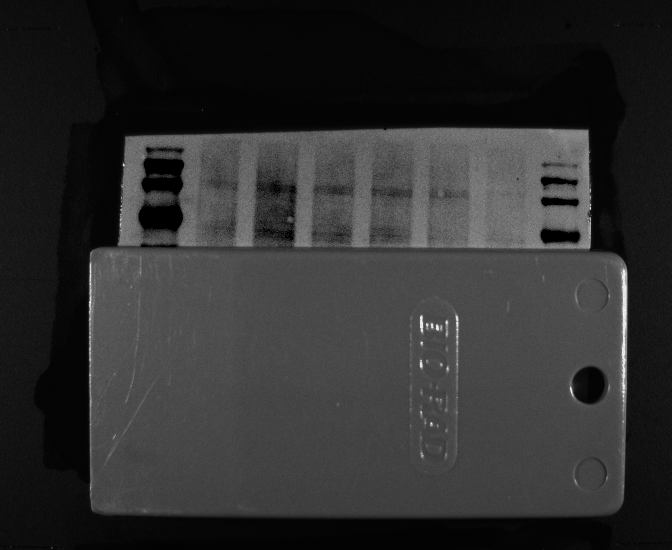

Supplement: Supplementary file 1 [file biomolecules-16-00926-s001.zip › biomolecules-4345458-WB/WB/scar formation/Collagen I/membrane 3/collegan i_8bit.tif]

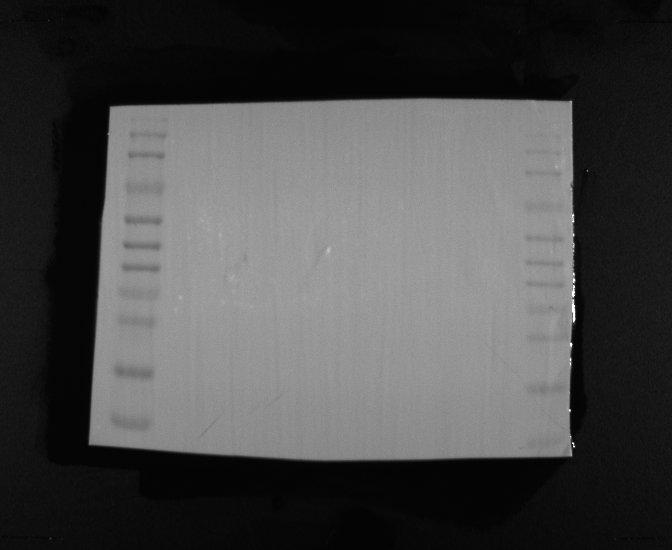

Supplement: Supplementary file 1 [file biomolecules-16-00926-s001.zip › biomolecules-4345458-WB/WB/scar formation/Collagen I/membrane 3/gapdh/1_8bit.tif]

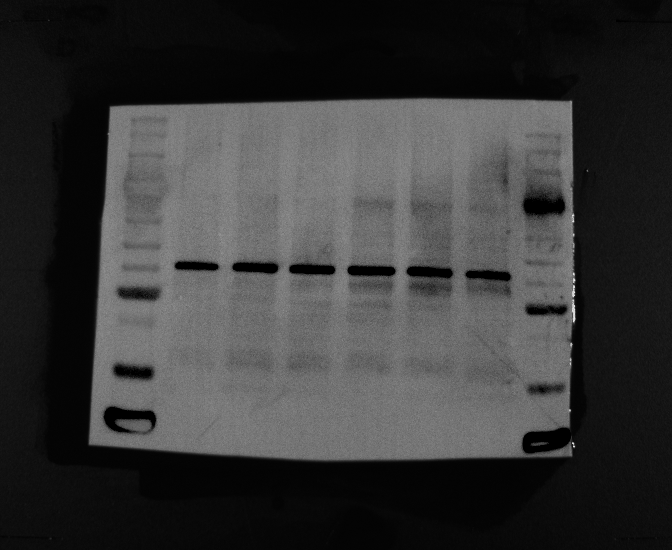

Supplement: Supplementary file 1 [file biomolecules-16-00926-s001.zip › biomolecules-4345458-WB/WB/scar formation/Collagen I/membrane 3/gapdh/gapdh_8bit.tif]

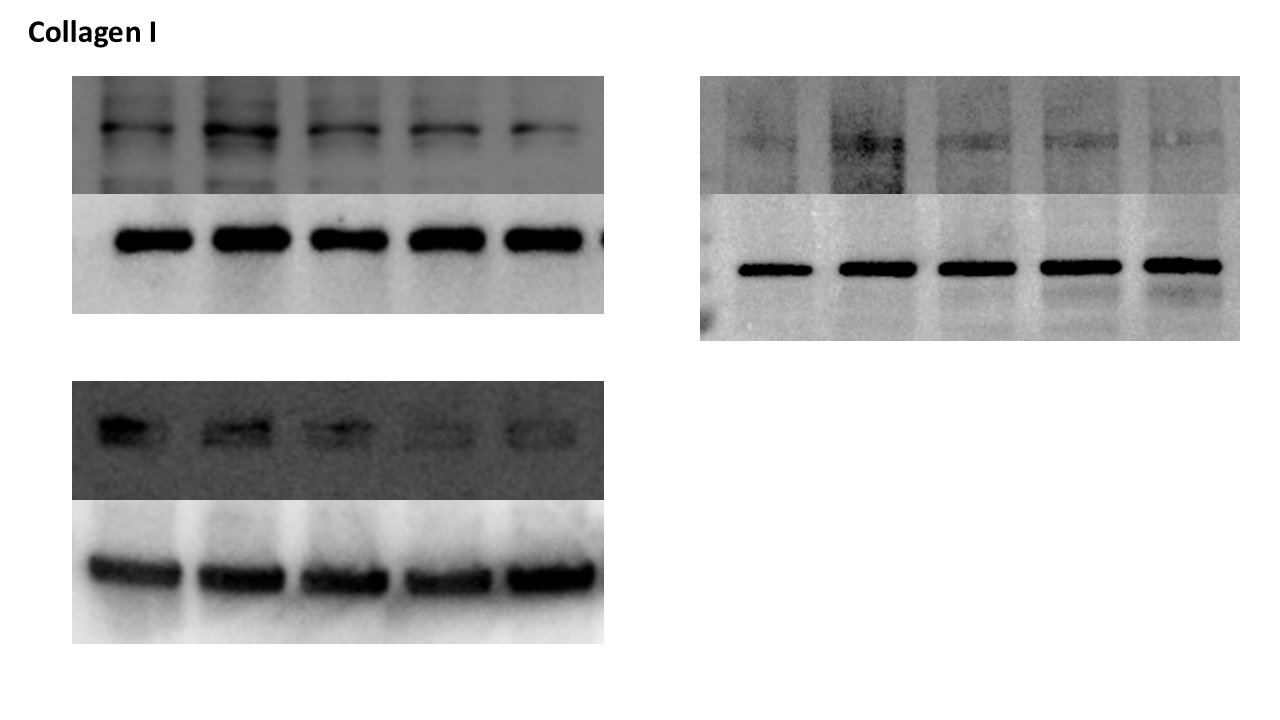

Supplement: Supplementary file 1 [file biomolecules-16-00926-s001.zip › biomolecules-4345458-WB/WB/scar formation/Collagen I/WB.tif]

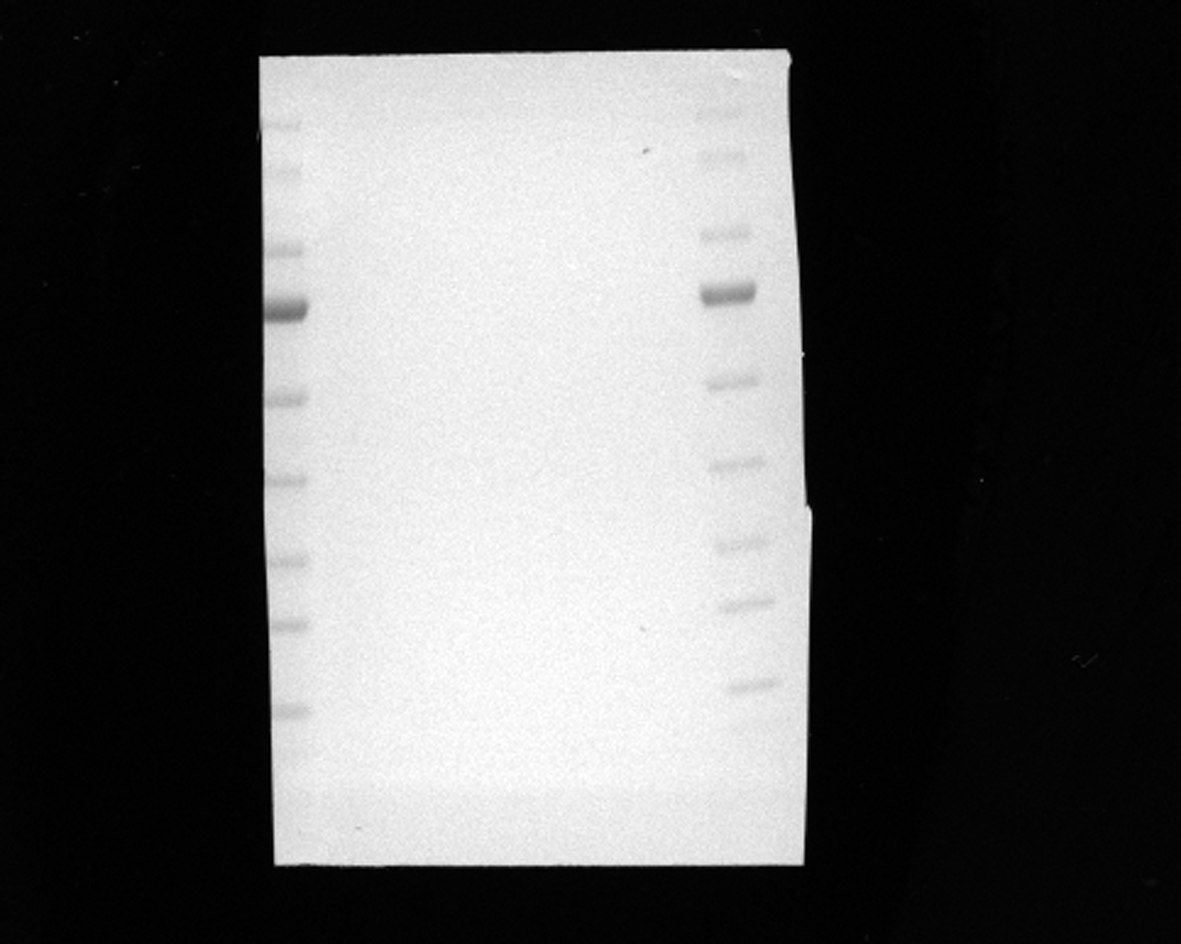

Supplement: Supplementary file 1 [file biomolecules-16-00926-s001.zip › biomolecules-4345458-WB/WB/scar formation/Collagen III/membrane 1/BF.tif]

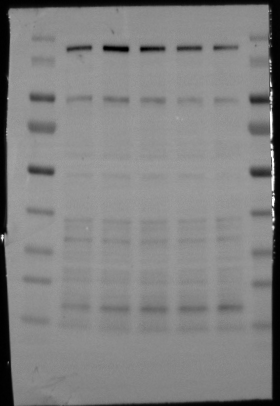

Supplement: Supplementary file 1 [file biomolecules-16-00926-s001.zip › biomolecules-4345458-WB/WB/scar formation/Collagen III/membrane 1/Collagen 3-1.tif]

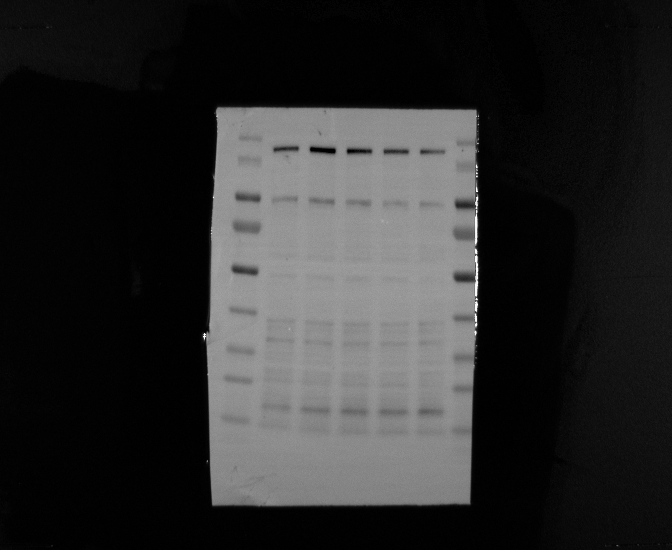

Supplement: Supplementary file 1 [file biomolecules-16-00926-s001.zip › biomolecules-4345458-WB/WB/scar formation/Collagen III/membrane 1/Collagen 3.tif]

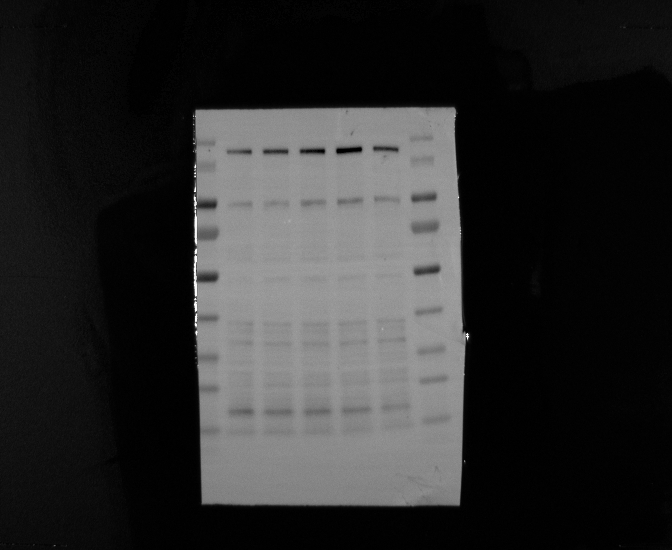

Supplement: Supplementary file 1 [file biomolecules-16-00926-s001.zip › biomolecules-4345458-WB/WB/scar formation/Collagen III/membrane 1/Collagen 3_2.tif]

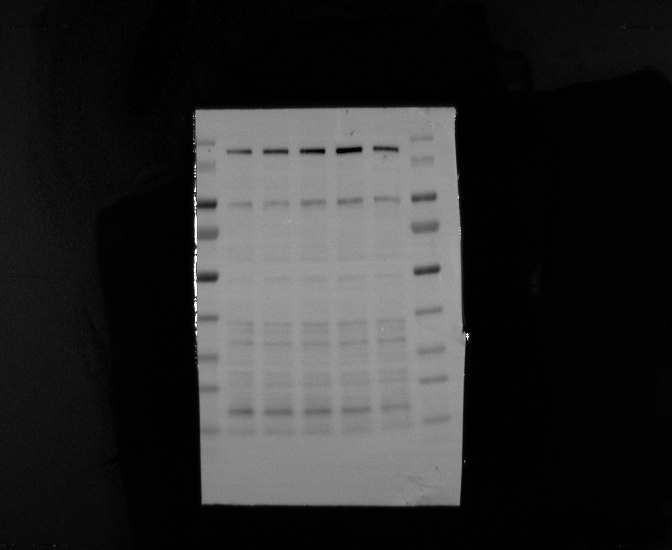

Supplement: Supplementary file 1 [file biomolecules-16-00926-s001.zip › biomolecules-4345458-WB/WB/scar formation/Collagen III/membrane 1/Collagen 3_3.tif]

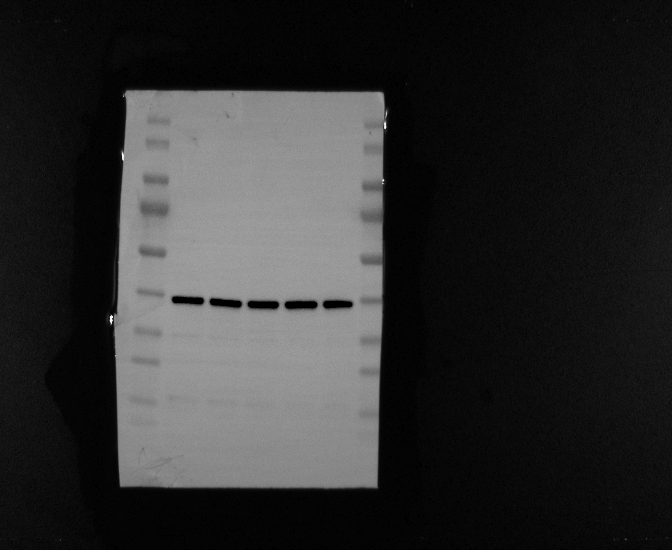

Supplement: Supplementary file 1 [file biomolecules-16-00926-s001.zip › biomolecules-4345458-WB/WB/scar formation/Collagen III/membrane 1/GAPDH.tif]

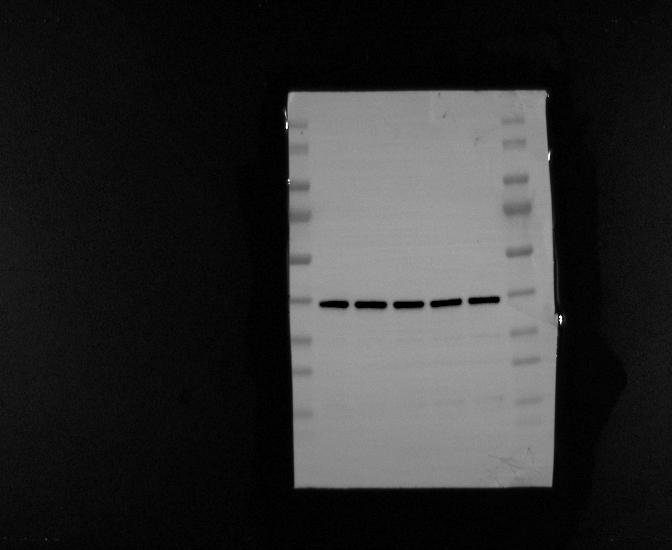

Supplement: Supplementary file 1 [file biomolecules-16-00926-s001.zip › biomolecules-4345458-WB/WB/scar formation/Collagen III/membrane 1/WB_20250911_171250_00.00_8bit_8bit.tif]

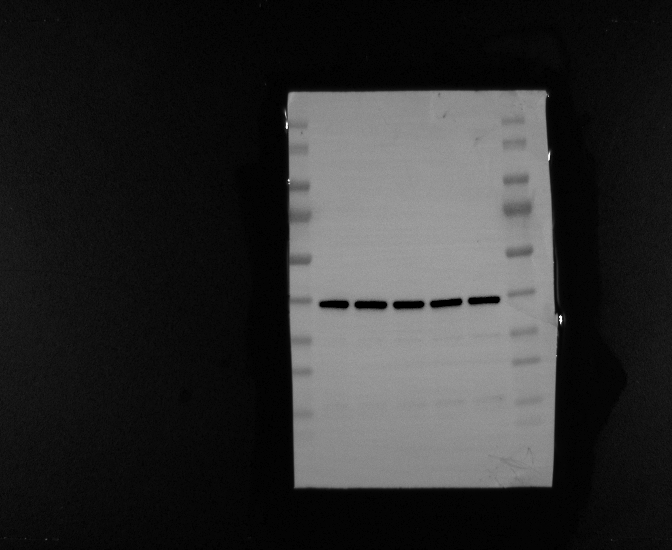

Supplement: Supplementary file 1 [file biomolecules-16-00926-s001.zip › biomolecules-4345458-WB/WB/scar formation/Collagen III/membrane 1/WB_20250911_192446_00.00.035_8bit.tif]

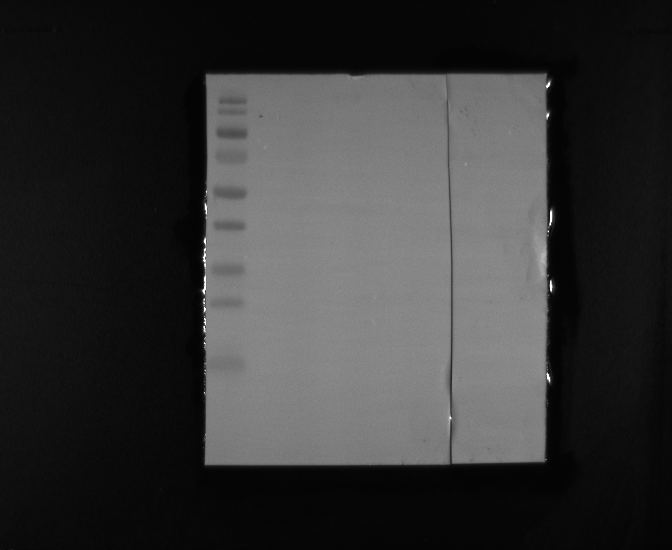

Supplement: Supplementary file 1 [file biomolecules-16-00926-s001.zip › biomolecules-4345458-WB/WB/scar formation/Collagen III/membrane 2/bright filed_8bit.tif]

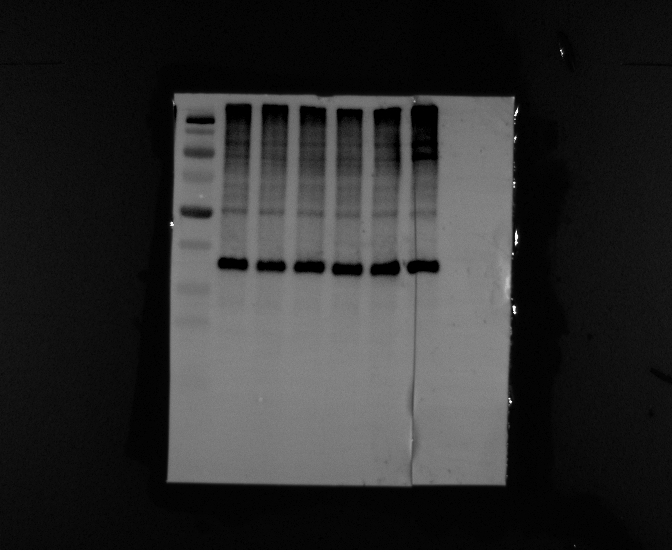

Supplement: Supplementary file 1 [file biomolecules-16-00926-s001.zip › biomolecules-4345458-WB/WB/scar formation/Collagen III/membrane 2/GAPDH_8bit.tif]

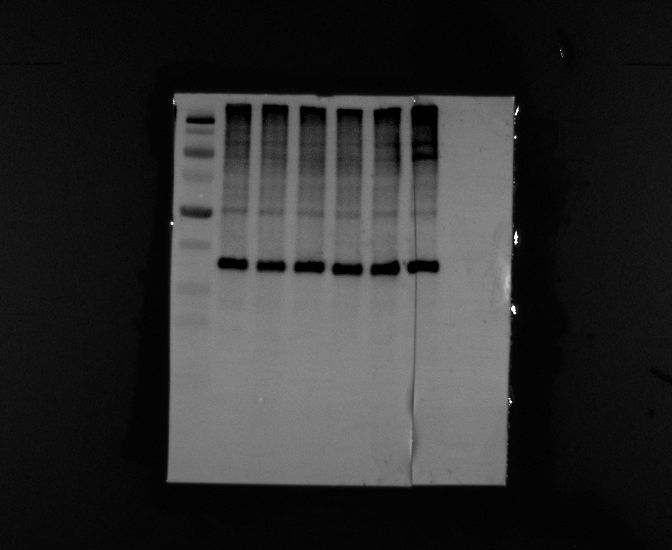

Supplement: Supplementary file 1 [file biomolecules-16-00926-s001.zip › biomolecules-4345458-WB/WB/scar formation/Collagen III/membrane 2/GAPDH_8bit_8bit.tif]

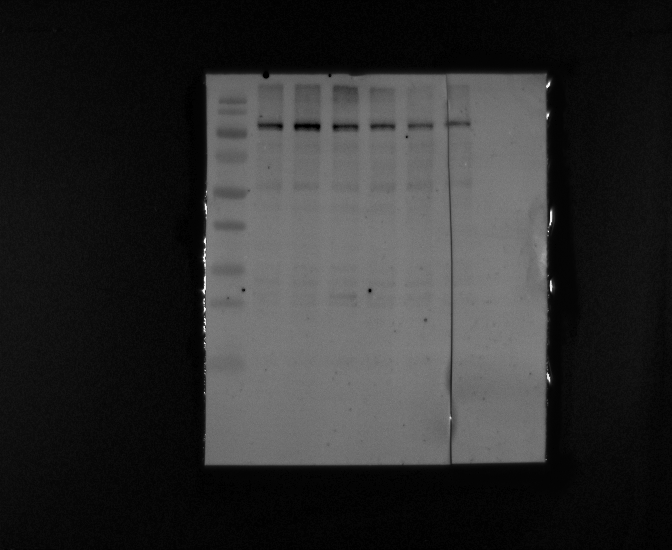

Supplement: Supplementary file 1 [file biomolecules-16-00926-s001.zip › biomolecules-4345458-WB/WB/scar formation/Collagen III/membrane 2/WB_20250415_COL1A1-样品图-EDITED-high_8bit_8bit_8bit.tif]

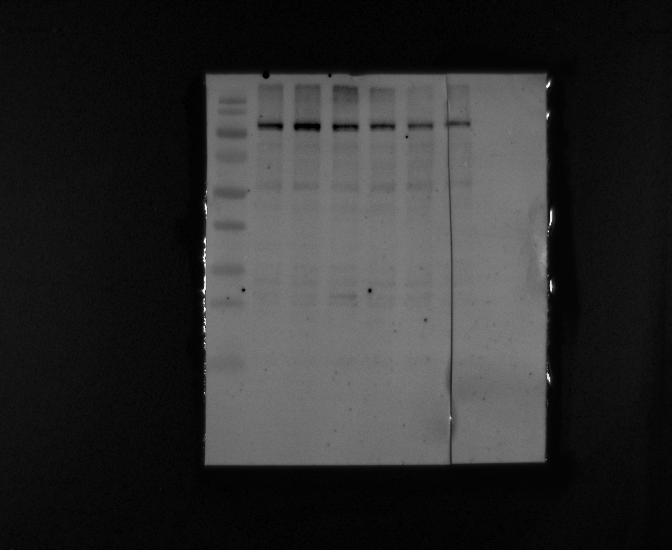

Supplement: Supplementary file 1 [file biomolecules-16-00926-s001.zip › biomolecules-4345458-WB/WB/scar formation/Collagen III/membrane 2/WB_20250421_194306_00.59.000_8bit(0).tif]

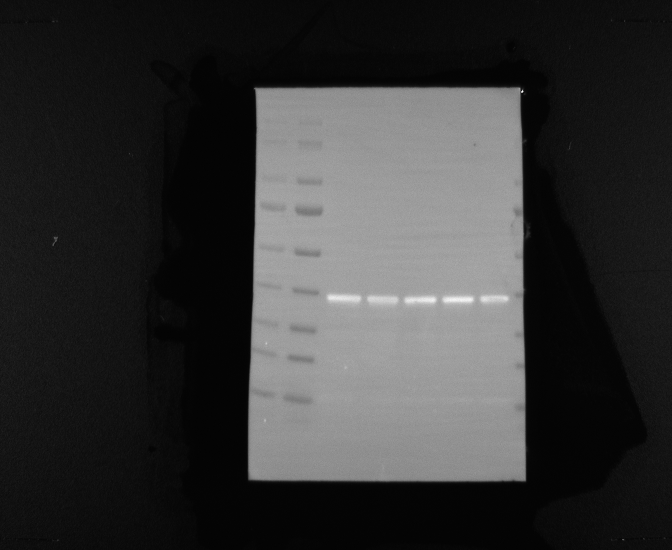

Supplement: Supplementary file 1 [file biomolecules-16-00926-s001.zip › biomolecules-4345458-WB/WB/scar formation/Collagen III/membrane 3/BRIGHT FIELD_8bit.tif]

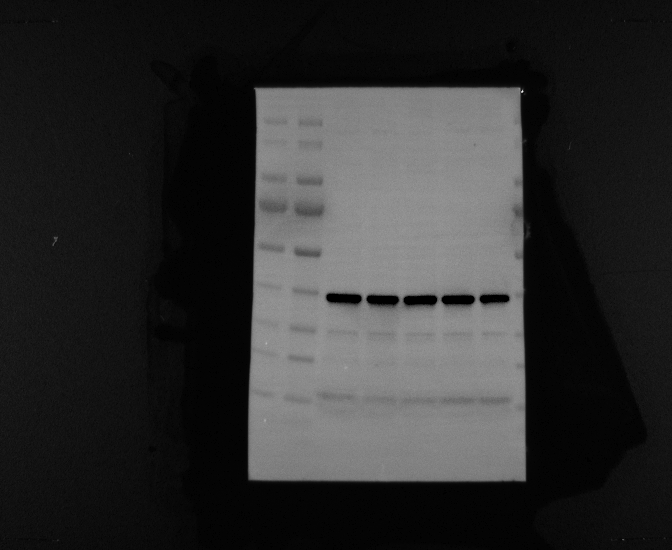

Supplement: Supplementary file 1 [file biomolecules-16-00926-s001.zip › biomolecules-4345458-WB/WB/scar formation/Collagen III/membrane 3/GAPDH_8bit.tif]

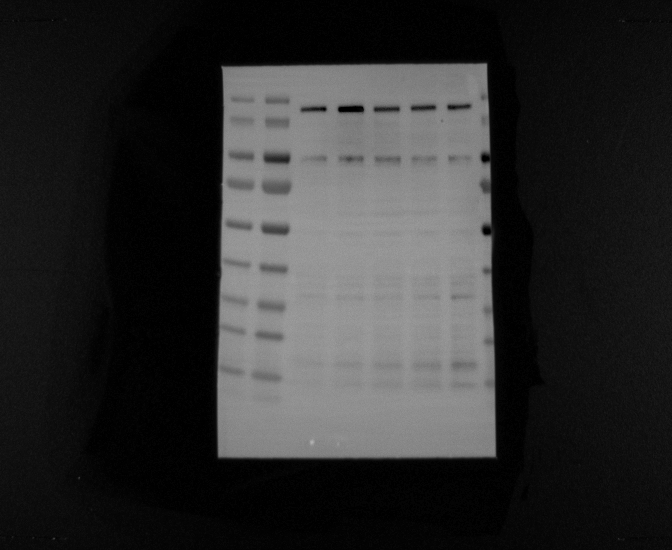

Supplement: Supplementary file 1 [file biomolecules-16-00926-s001.zip › biomolecules-4345458-WB/WB/scar formation/Collagen III/membrane 3/WB_20250907_202857_00.00.969_8bit-1.tif]

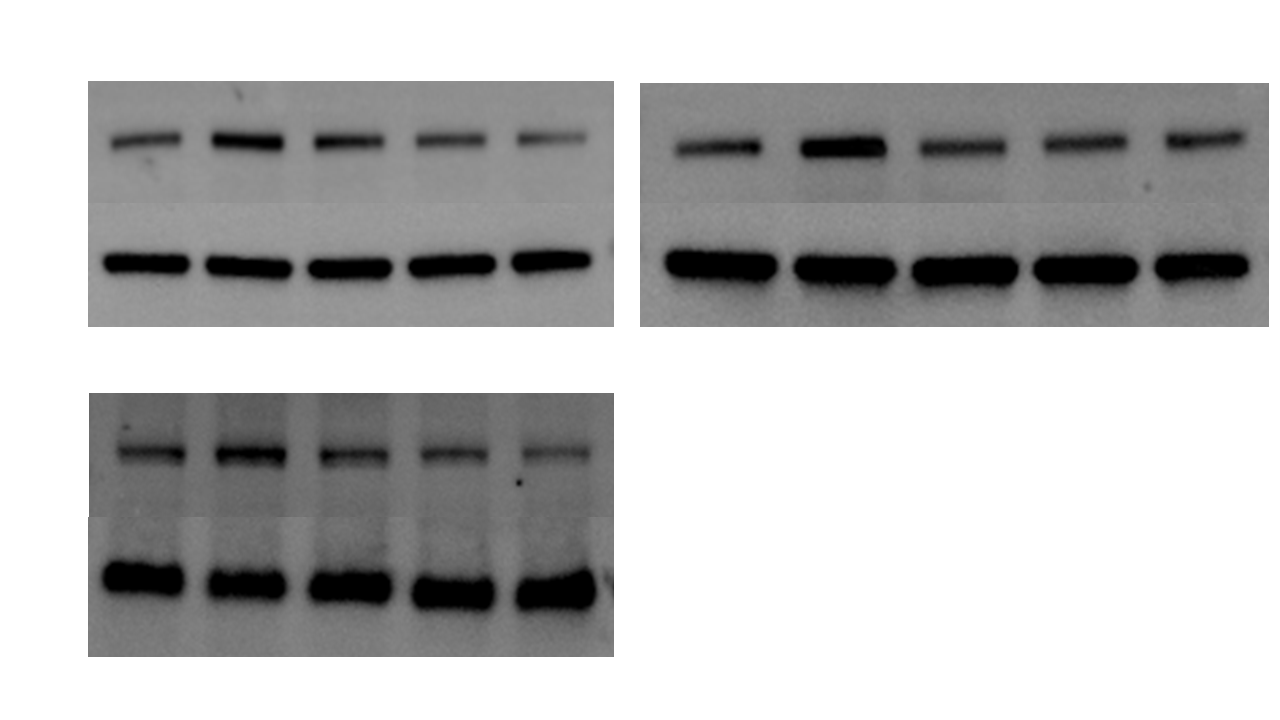

Supplement: Supplementary file 1 [file biomolecules-16-00926-s001.zip › biomolecules-4345458-WB/WB/scar formation/Collagen III/WB.tif]
