# Supplementary figures and images for: Piceatannol Promotes Burn Wound Healing by Coordinately Modulating Inflammation–Oxidative Stress Crosstalk, Angiogenesis, and Fibrotic Remodeling (part 1 of 2)
Source: Biomolecules. 2026 Jun 23;16(7):926. doi: 10.3390/biom16070926 (PMC13406660; doi:10.3390/biom16070926)

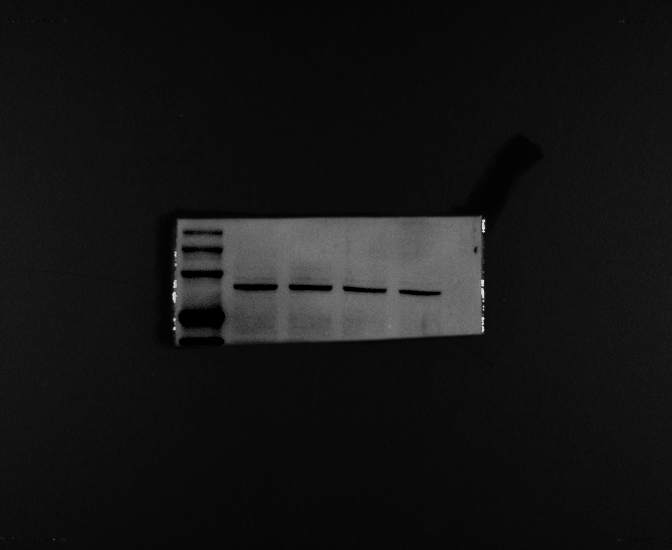

Supplement: Supplementary file 1 [file biomolecules-16-00926-s001.zip › biomolecules-4345458-WB/WB/Angiogenesis/P-stat3_stat3/membrane 1/membrane1_8bit.tif]

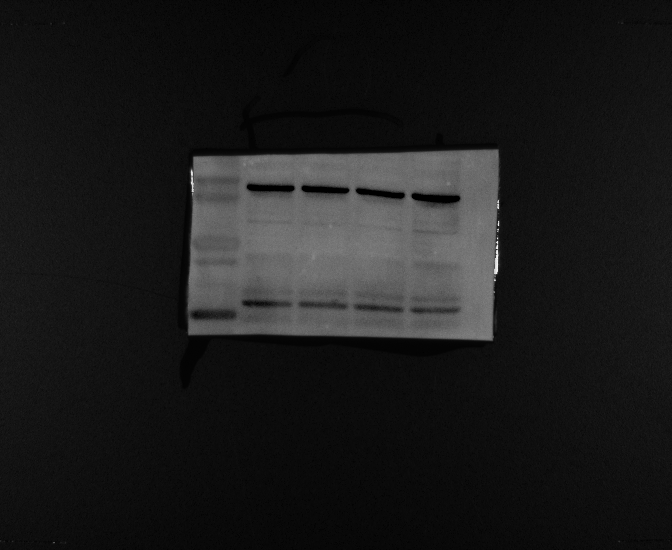

Supplement: Supplementary file 1 [file biomolecules-16-00926-s001.zip › biomolecules-4345458-WB/WB/Angiogenesis/P-stat3_stat3/membrane 1/membrane1_GAPDH8bit.tif]

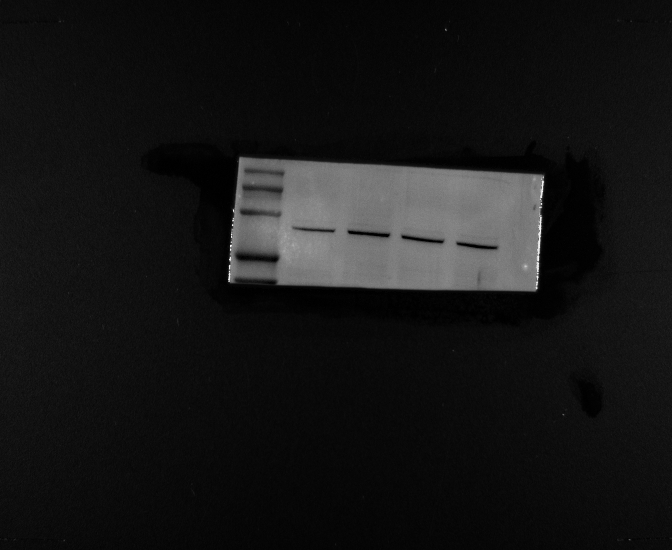

Supplement: Supplementary file 1 [file biomolecules-16-00926-s001.zip › biomolecules-4345458-WB/WB/Angiogenesis/P-stat3_stat3/membrane 1/P-stat31_8bit.tif]

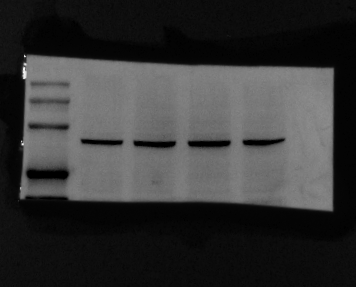

Supplement: Supplementary file 1 [file biomolecules-16-00926-s001.zip › biomolecules-4345458-WB/WB/Angiogenesis/P-stat3_stat3/membrane 2/2222_8bit_8bit-1.tif]

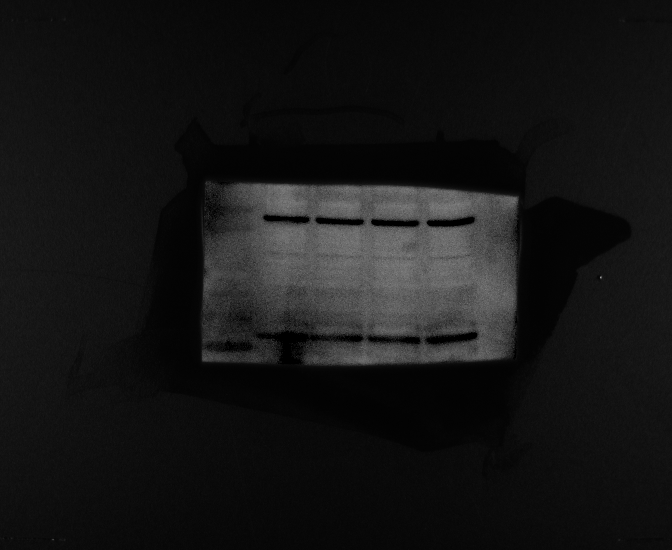

Supplement: Supplementary file 1 [file biomolecules-16-00926-s001.zip › biomolecules-4345458-WB/WB/Angiogenesis/P-stat3_stat3/membrane 2/membrane2_GAPDH8bit.tif]

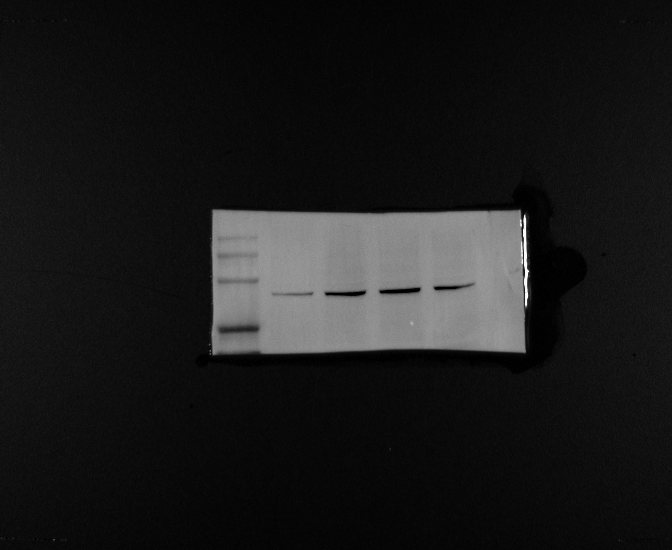

Supplement: Supplementary file 1 [file biomolecules-16-00926-s001.zip › biomolecules-4345458-WB/WB/Angiogenesis/P-stat3_stat3/membrane 2/P-stat3.tif]

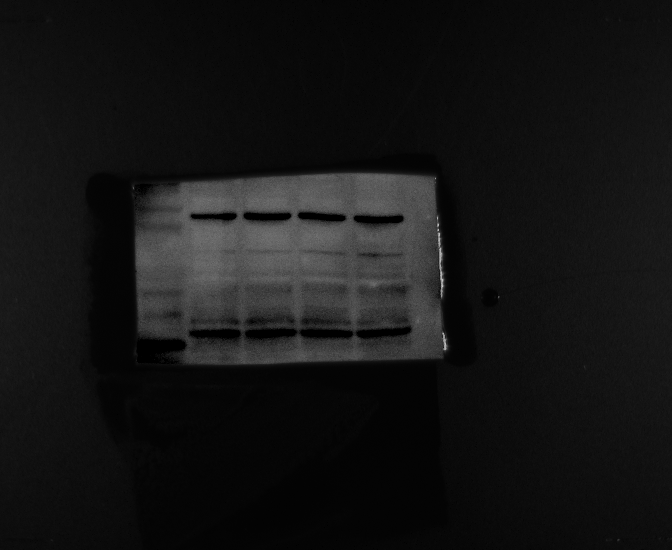

Supplement: Supplementary file 1 [file biomolecules-16-00926-s001.zip › biomolecules-4345458-WB/WB/Angiogenesis/P-stat3_stat3/membrane 3/membrane3_8bit.tif]

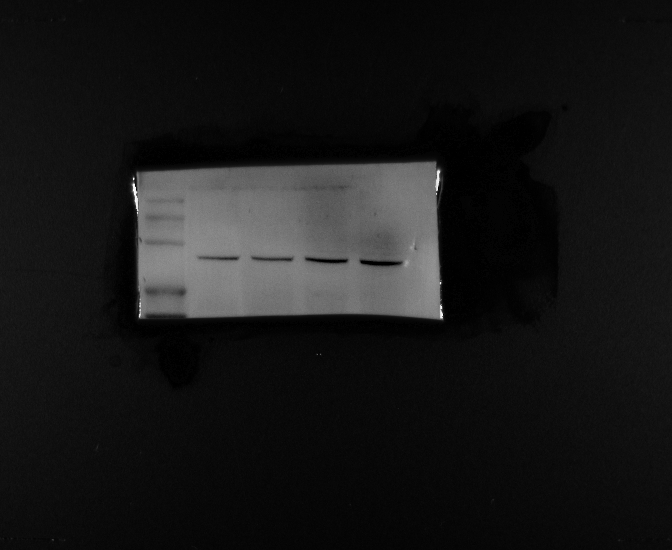

Supplement: Supplementary file 1 [file biomolecules-16-00926-s001.zip › biomolecules-4345458-WB/WB/Angiogenesis/P-stat3_stat3/membrane 3/P-stat3_1.tif]

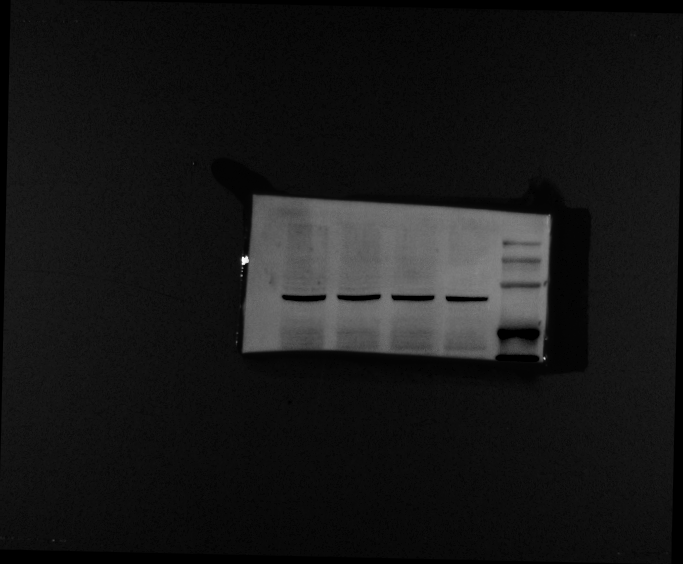

Supplement: Supplementary file 1 [file biomolecules-16-00926-s001.zip › biomolecules-4345458-WB/WB/Angiogenesis/P-stat3_stat3/membrane 3/total_stat3.tif]

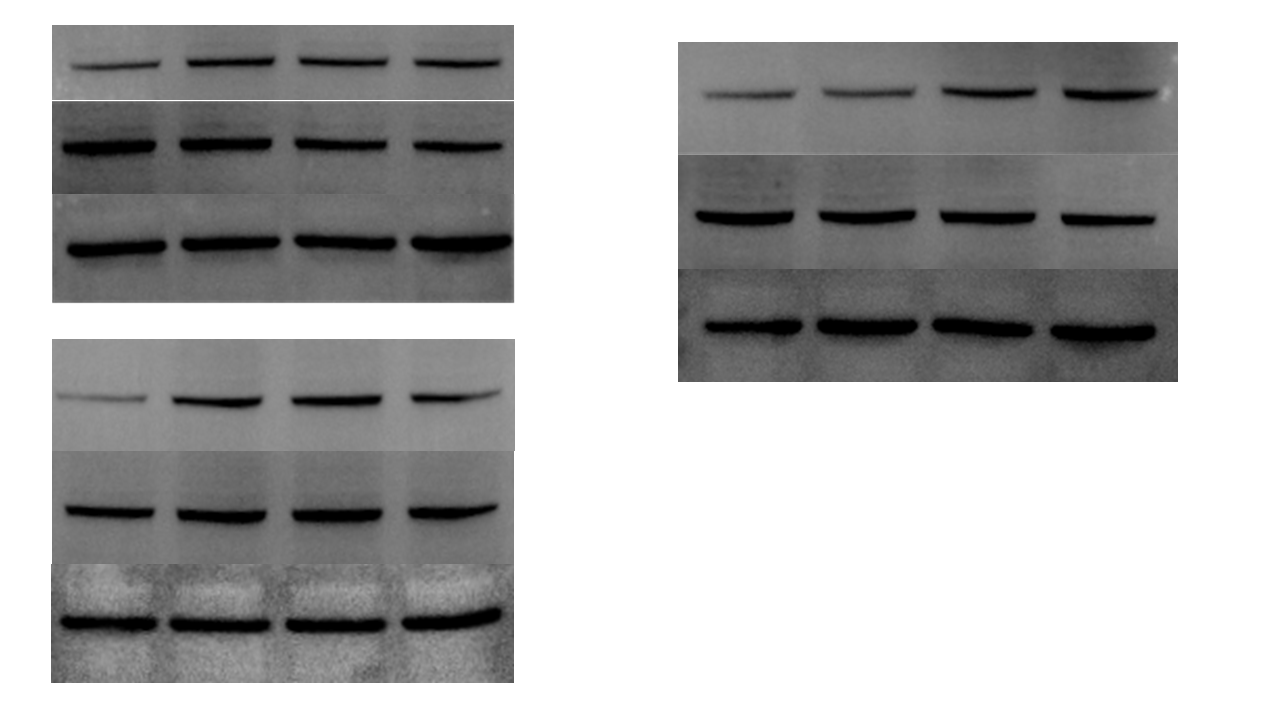

Supplement: Supplementary file 1 [file biomolecules-16-00926-s001.zip › biomolecules-4345458-WB/WB/Angiogenesis/P-stat3_stat3/WB.tif]

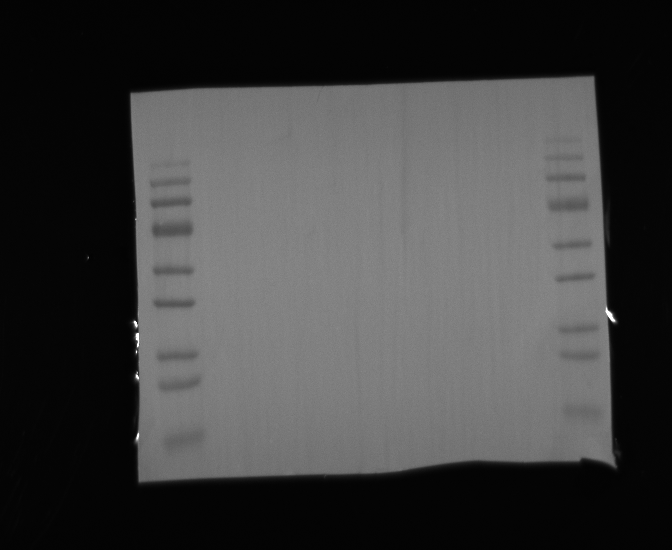

Supplement: Supplementary file 1 [file biomolecules-16-00926-s001.zip › biomolecules-4345458-WB/WB/Angiogenesis/VEGF/membrane1/-Bright_filed-.tif]

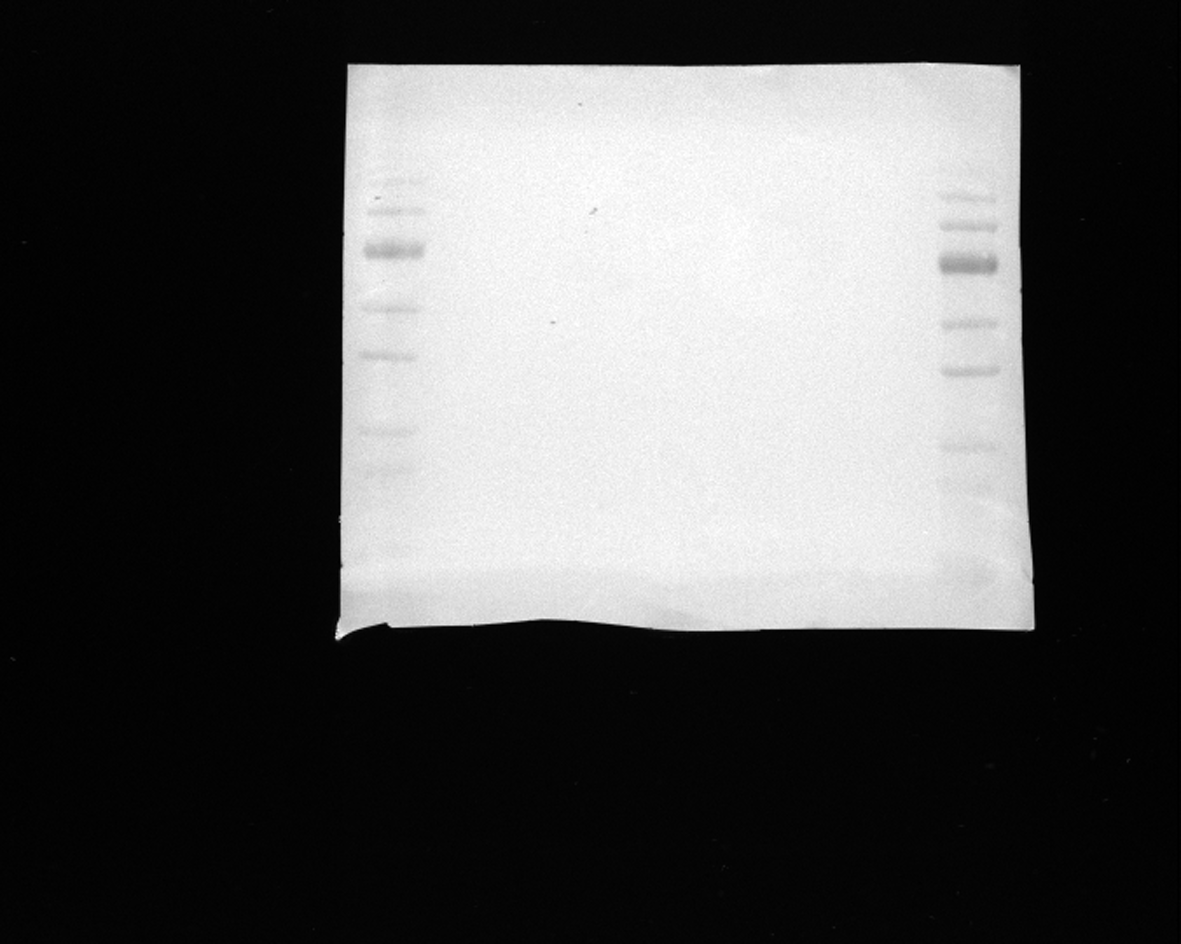

Supplement: Supplementary file 1 [file biomolecules-16-00926-s001.zip › biomolecules-4345458-WB/WB/Angiogenesis/VEGF/membrane1/CHEMI_04222025_182852_(Membrane).tif]

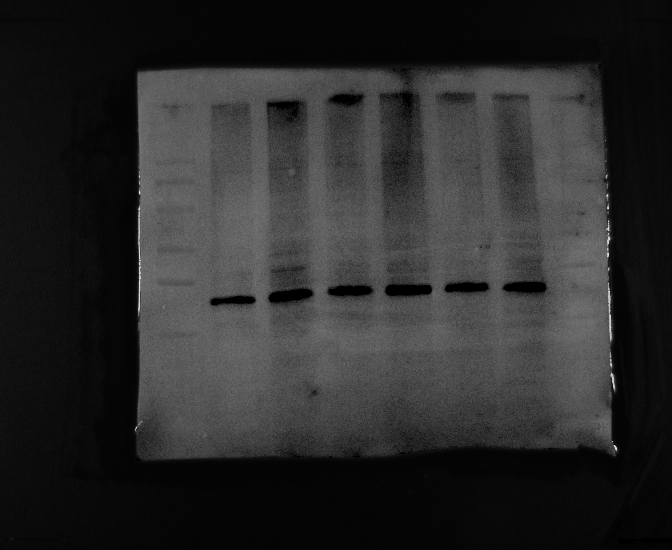

Supplement: Supplementary file 1 [file biomolecules-16-00926-s001.zip › biomolecules-4345458-WB/WB/Angiogenesis/VEGF/membrane1/GAPDH.tif]

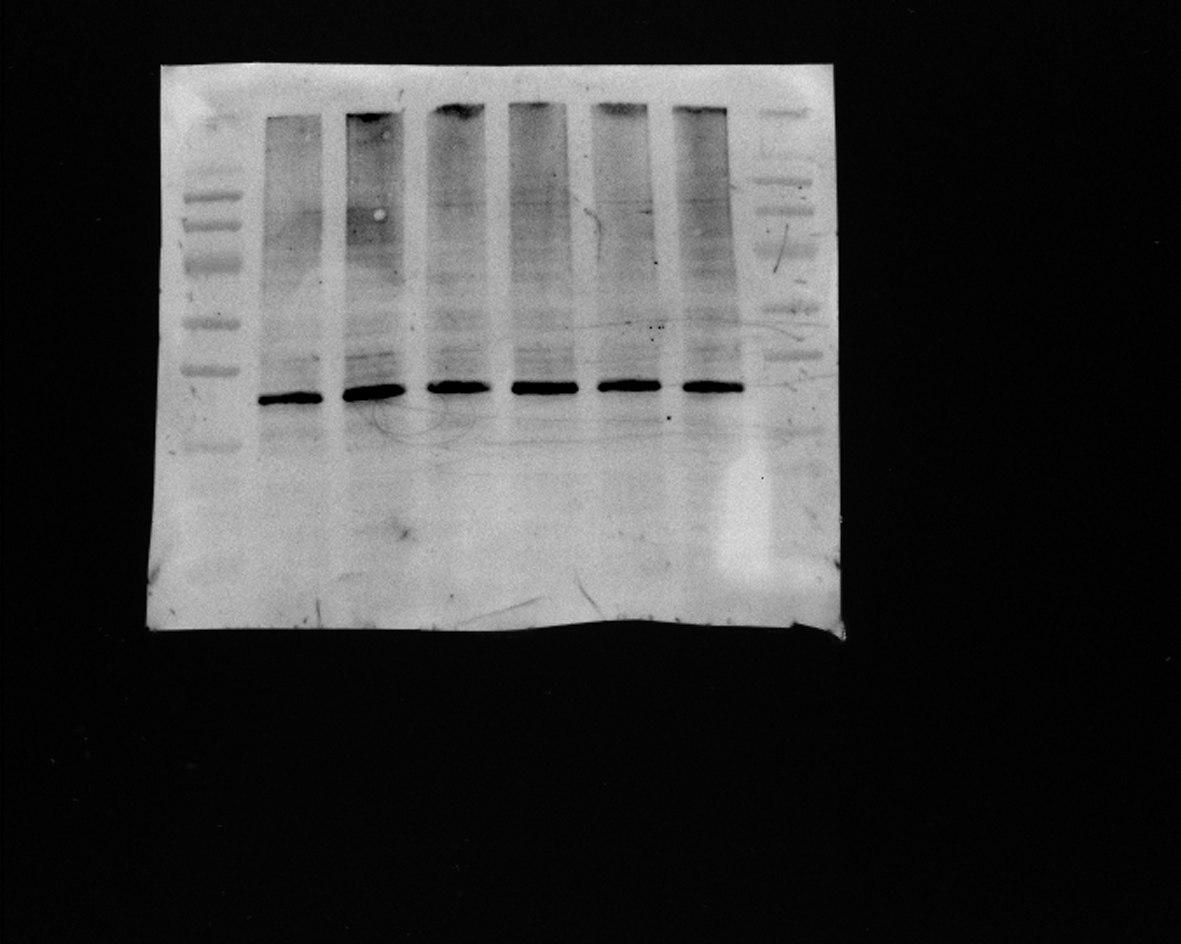

Supplement: Supplementary file 1 [file biomolecules-16-00926-s001.zip › biomolecules-4345458-WB/WB/Angiogenesis/VEGF/membrane1/GAPDH1.tif]

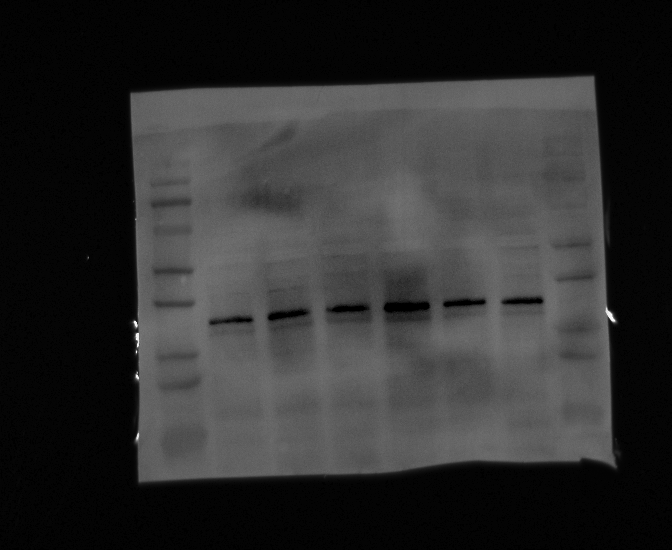

Supplement: Supplementary file 1 [file biomolecules-16-00926-s001.zip › biomolecules-4345458-WB/WB/Angiogenesis/VEGF/membrane1/Vegf_5s_01_8bit_8bit.tif]

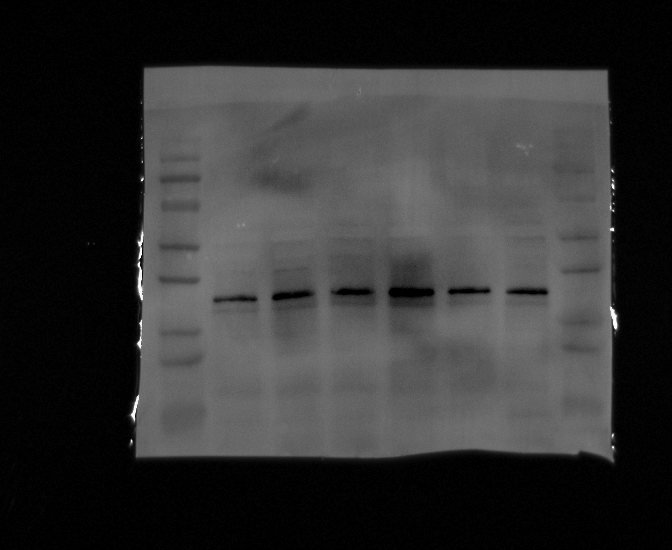

Supplement: Supplementary file 1 [file biomolecules-16-00926-s001.zip › biomolecules-4345458-WB/WB/Angiogenesis/VEGF/membrane1/Vegf_5s_02_8bit_8bit_8bit.tif]

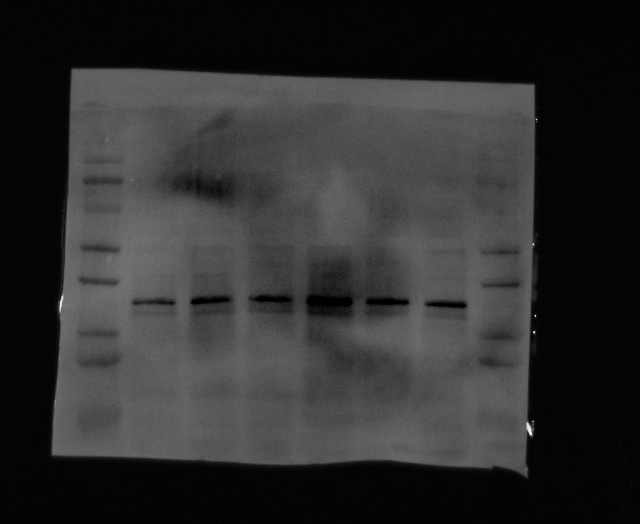

Supplement: Supplementary file 1 [file biomolecules-16-00926-s001.zip › biomolecules-4345458-WB/WB/Angiogenesis/VEGF/membrane1/Vegf_5s_03_8bit_filped_02_8bit_8b.tif]

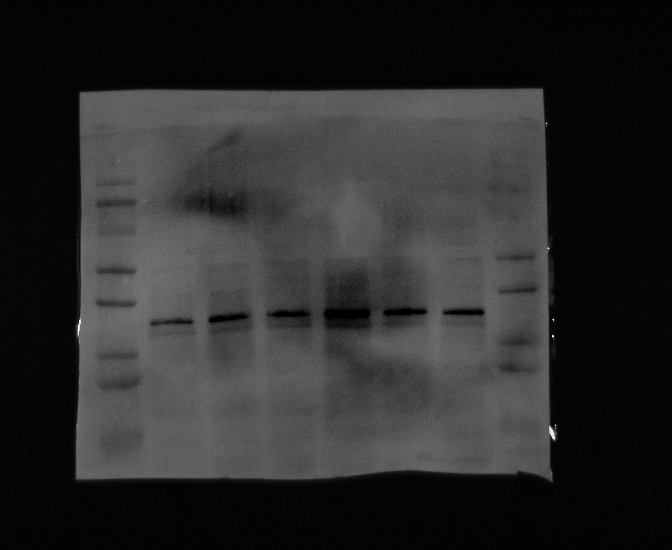

Supplement: Supplementary file 1 [file biomolecules-16-00926-s001.zip › biomolecules-4345458-WB/WB/Angiogenesis/VEGF/membrane1/Vegf_5s_03_8bit_filped_02_8bit_8bit.tif]

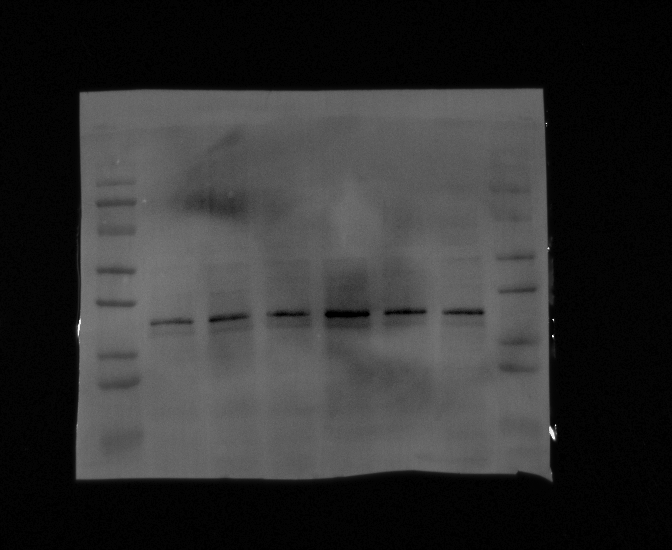

Supplement: Supplementary file 1 [file biomolecules-16-00926-s001.zip › biomolecules-4345458-WB/WB/Angiogenesis/VEGF/membrane1/Vegf_5s_03_8bit_filped_8bit.tif]

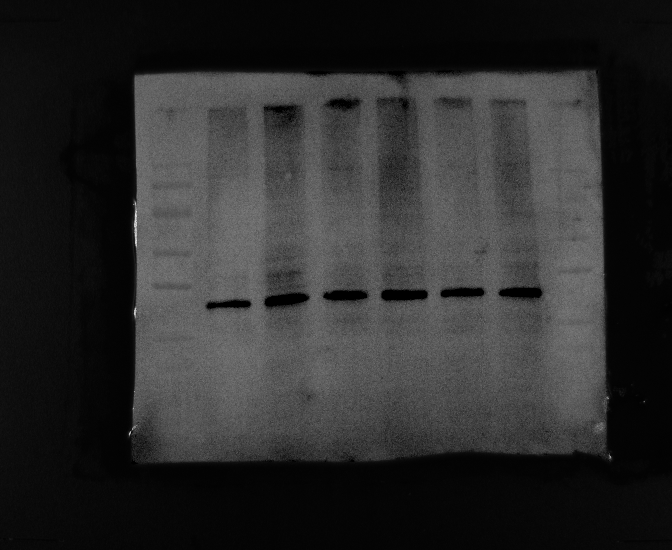

Supplement: Supplementary file 1 [file biomolecules-16-00926-s001.zip › biomolecules-4345458-WB/WB/Angiogenesis/VEGF/membrane1/WB_20250419_180728_00.05.000_02_8bit.tif]

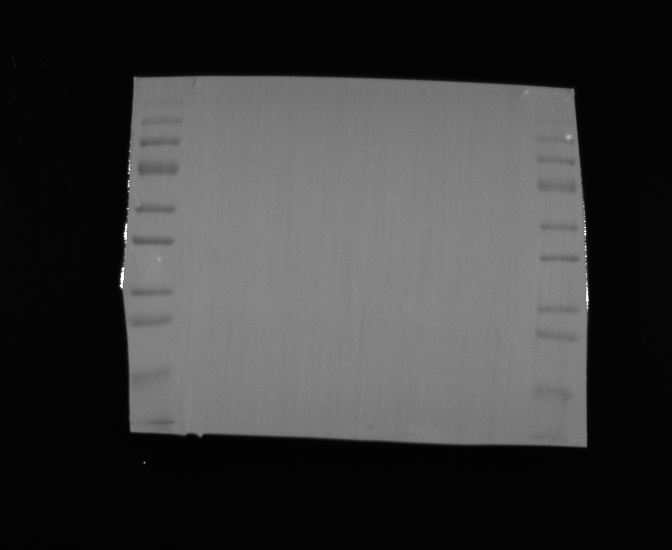

Supplement: Supplementary file 1 [file biomolecules-16-00926-s001.zip › biomolecules-4345458-WB/WB/Angiogenesis/VEGF/membrane2/bright filed_02-8bit_8bit.tif]

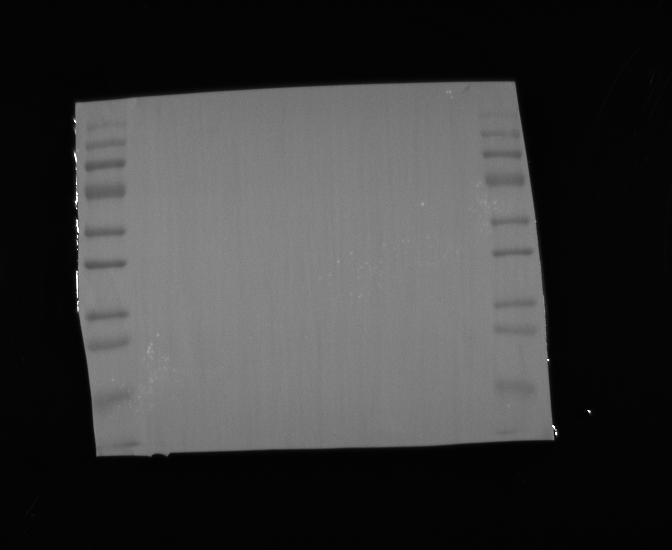

Supplement: Supplementary file 1 [file biomolecules-16-00926-s001.zip › biomolecules-4345458-WB/WB/Angiogenesis/VEGF/membrane2/bright filed_8bit.tif]

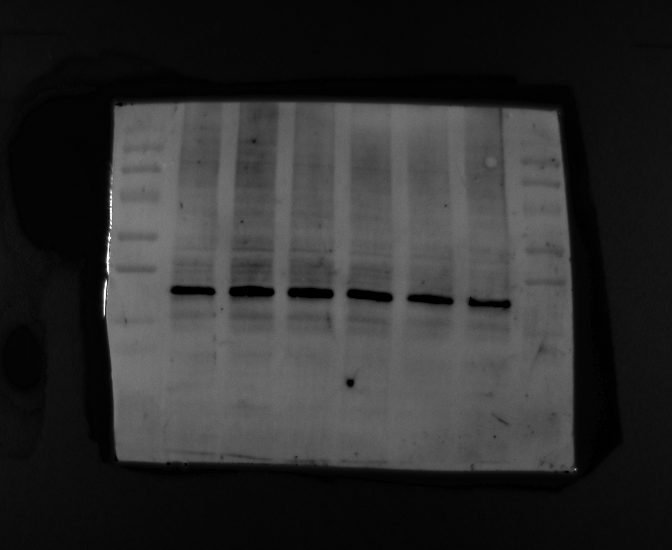

Supplement: Supplementary file 1 [file biomolecules-16-00926-s001.zip › biomolecules-4345458-WB/WB/Angiogenesis/VEGF/membrane2/GAPDH.tif]

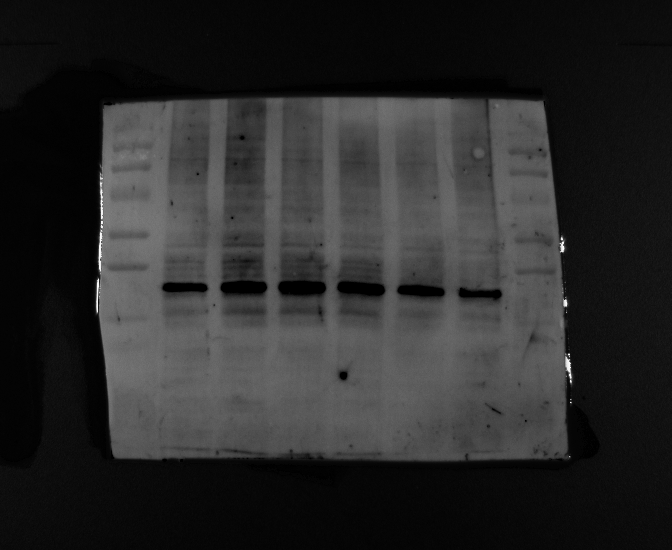

Supplement: Supplementary file 1 [file biomolecules-16-00926-s001.zip › biomolecules-4345458-WB/WB/Angiogenesis/VEGF/membrane2/GAPDH2.tif]

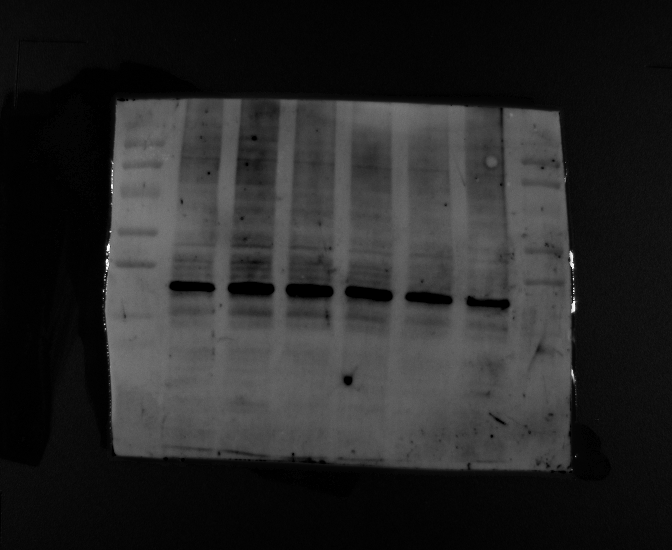

Supplement: Supplementary file 1 [file biomolecules-16-00926-s001.zip › biomolecules-4345458-WB/WB/Angiogenesis/VEGF/membrane2/GAPDH3.tif]

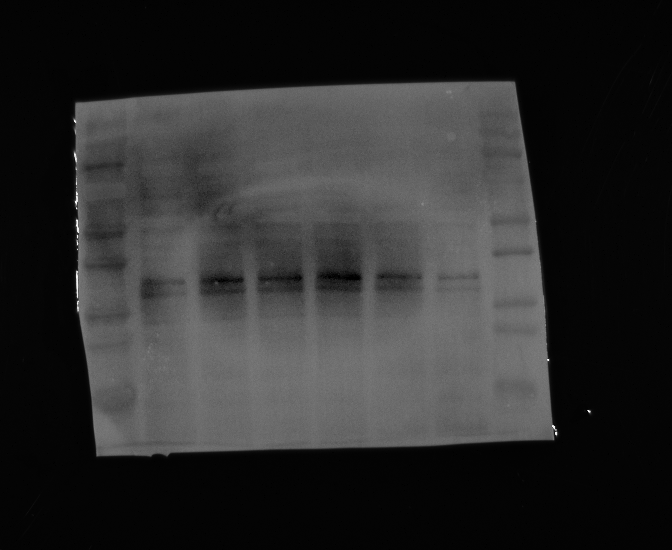

Supplement: Supplementary file 1 [file biomolecules-16-00926-s001.zip › biomolecules-4345458-WB/WB/Angiogenesis/VEGF/membrane2/WB_20250419_180728_00.05.000_1_8bit.tif]

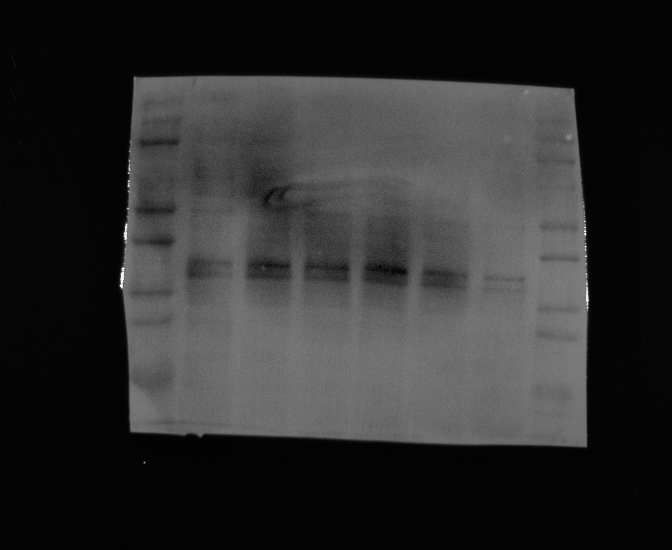

Supplement: Supplementary file 1 [file biomolecules-16-00926-s001.zip › biomolecules-4345458-WB/WB/Angiogenesis/VEGF/membrane2/WB_20250419_180728_00.05.000_2_8bit_8bit.tif]

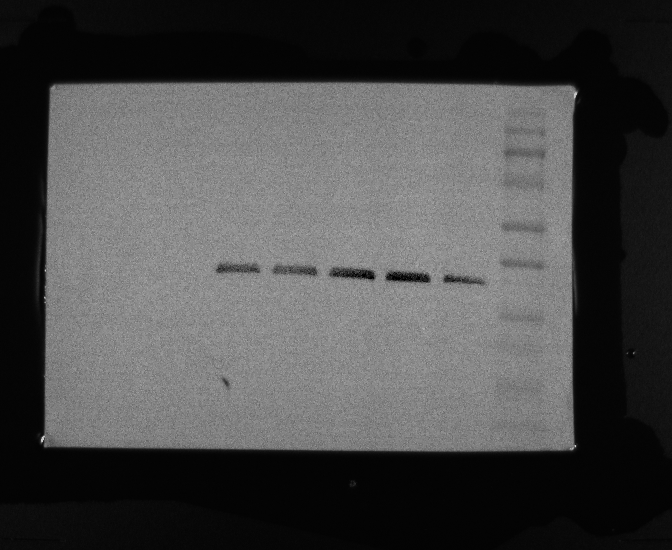

Supplement: Supplementary file 1 [file biomolecules-16-00926-s001.zip › biomolecules-4345458-WB/WB/Angiogenesis/VEGF/membrane3/10S_8bit_8bit.tif]

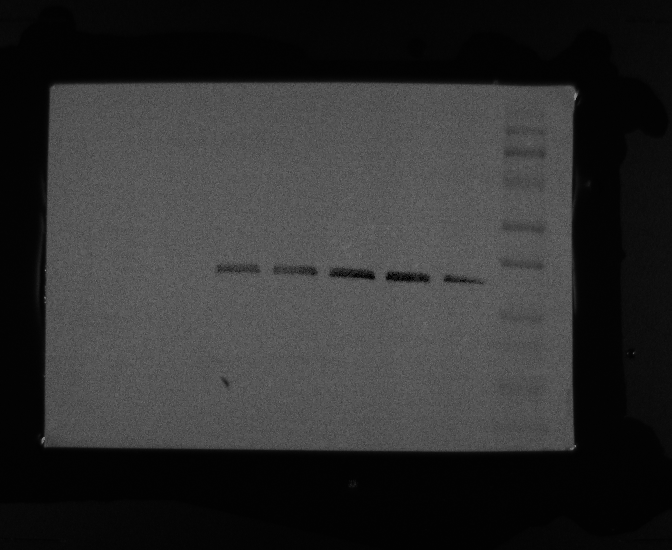

Supplement: Supplementary file 1 [file biomolecules-16-00926-s001.zip › biomolecules-4345458-WB/WB/Angiogenesis/VEGF/membrane3/3S_8bit.tif]

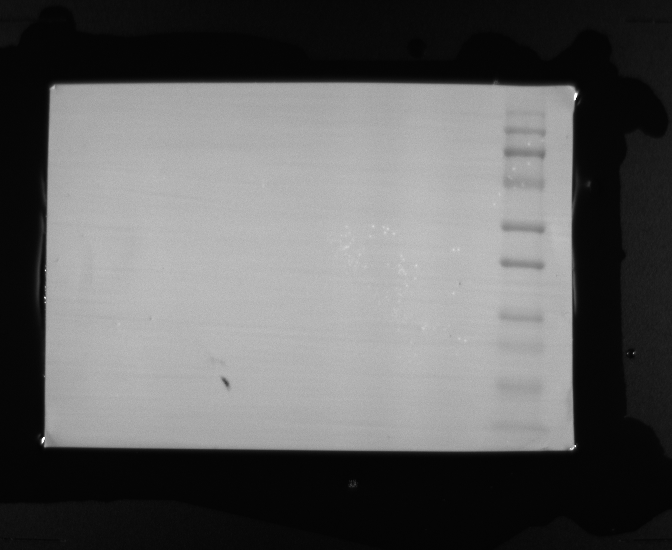

Supplement: Supplementary file 1 [file biomolecules-16-00926-s001.zip › biomolecules-4345458-WB/WB/Angiogenesis/VEGF/membrane3/BRIGHT FIELD_8bit.tif]

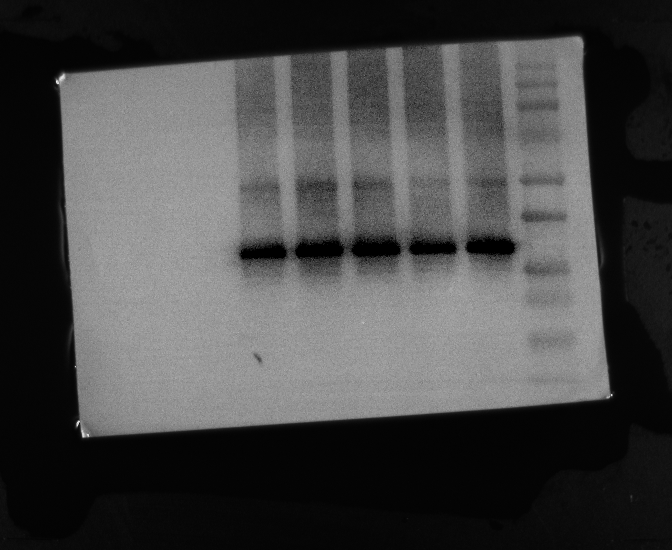

Supplement: Supplementary file 1 [file biomolecules-16-00926-s001.zip › biomolecules-4345458-WB/WB/Angiogenesis/VEGF/membrane3/GAPDH.tif]

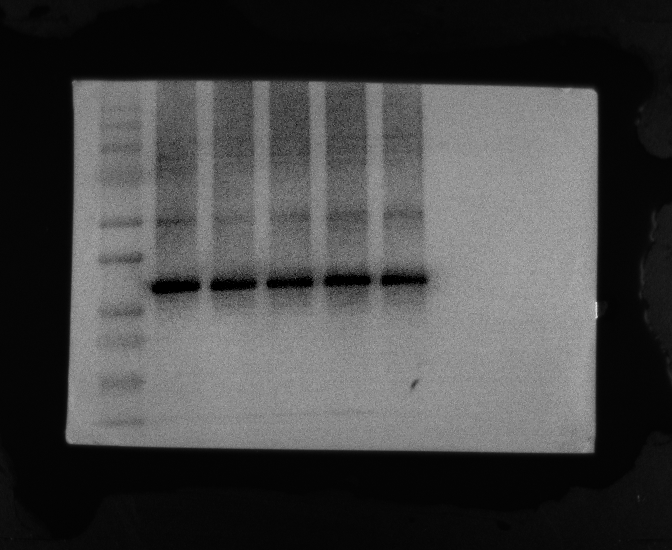

Supplement: Supplementary file 1 [file biomolecules-16-00926-s001.zip › biomolecules-4345458-WB/WB/Angiogenesis/VEGF/membrane3/GAPDH2.tif]

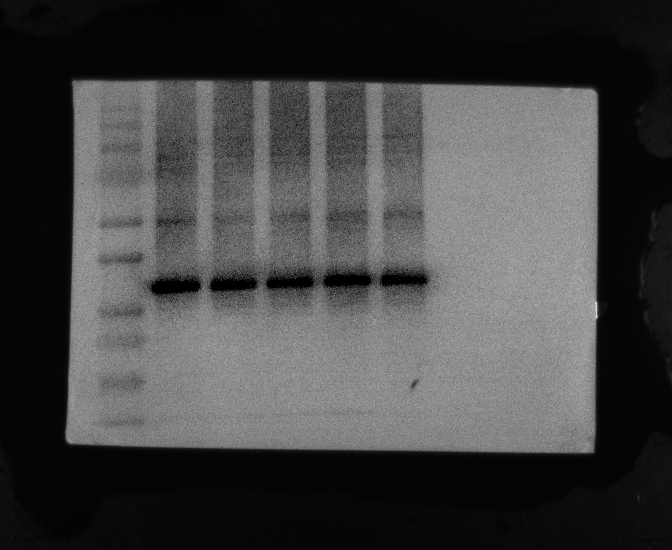

Supplement: Supplementary file 1 [file biomolecules-16-00926-s001.zip › biomolecules-4345458-WB/WB/Angiogenesis/VEGF/membrane3/GAPDH3.tif]

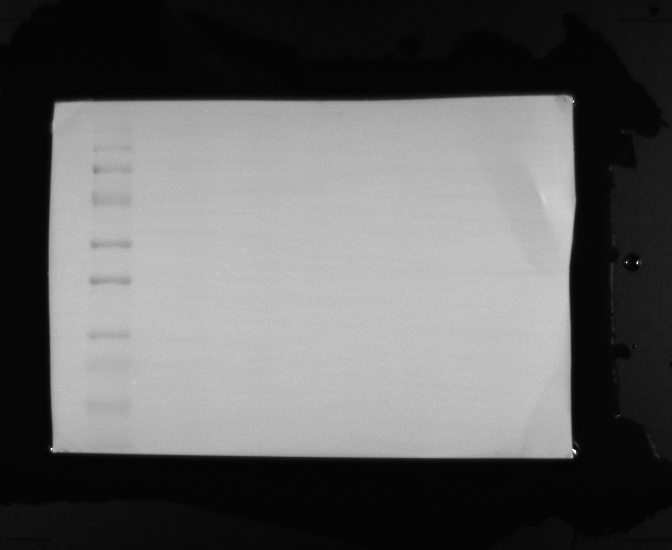

Supplement: Supplementary file 1 [file biomolecules-16-00926-s001.zip › biomolecules-4345458-WB/WB/Angiogenesis/VEGF/membrane4/BRIGHT FIELD.tif]

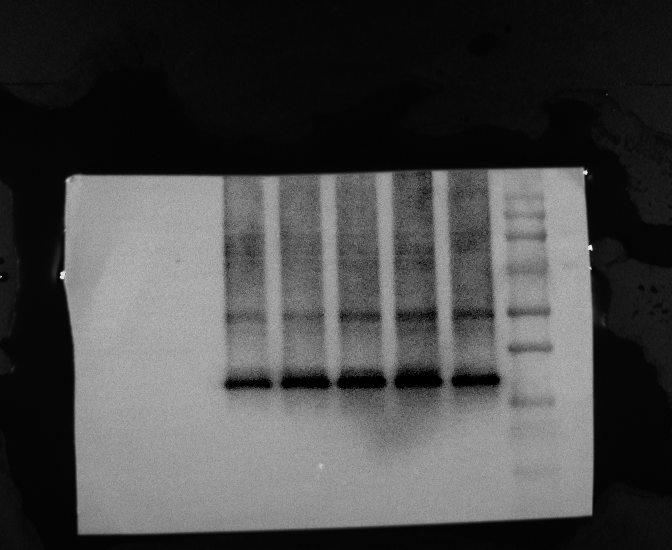

Supplement: Supplementary file 1 [file biomolecules-16-00926-s001.zip › biomolecules-4345458-WB/WB/Angiogenesis/VEGF/membrane4/GAPDH.tif]

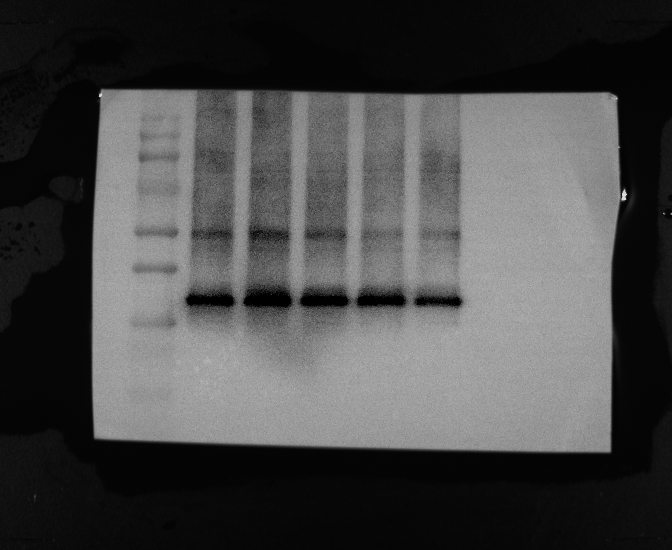

Supplement: Supplementary file 1 [file biomolecules-16-00926-s001.zip › biomolecules-4345458-WB/WB/Angiogenesis/VEGF/membrane4/GAPDH2.tif]

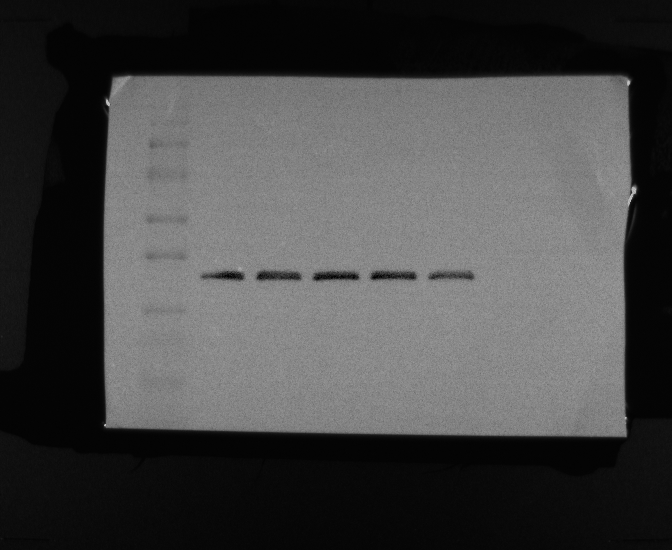

Supplement: Supplementary file 1 [file biomolecules-16-00926-s001.zip › biomolecules-4345458-WB/WB/Angiogenesis/VEGF/membrane4/VEGF.tif]

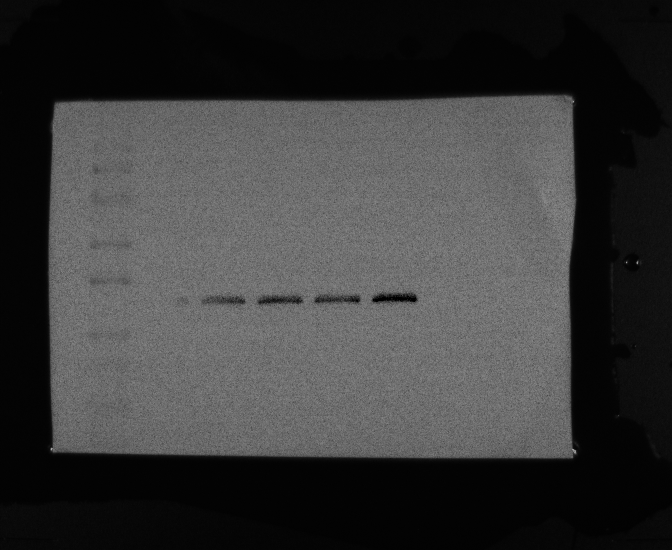

Supplement: Supplementary file 1 [file biomolecules-16-00926-s001.zip › biomolecules-4345458-WB/WB/Angiogenesis/VEGF/membrane4/VEGF_10S_8bit_8bit_8bit.tif]

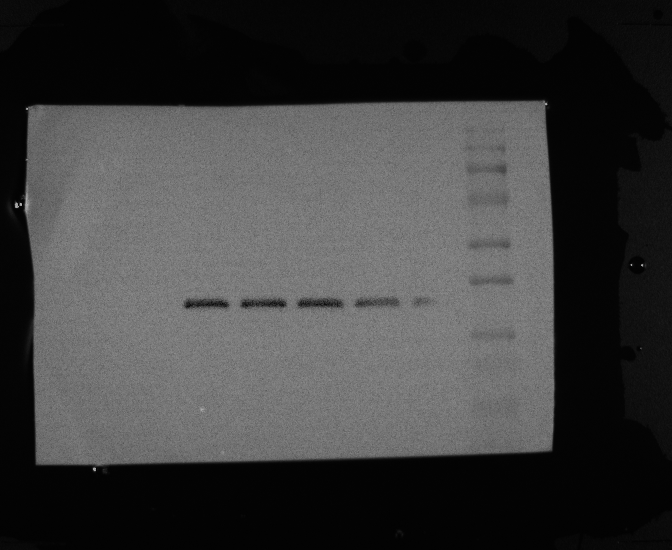

Supplement: Supplementary file 1 [file biomolecules-16-00926-s001.zip › biomolecules-4345458-WB/WB/Angiogenesis/VEGF/membrane4/VEGF_WB_20250807_165953_00.03.000_3_8bit.tif]

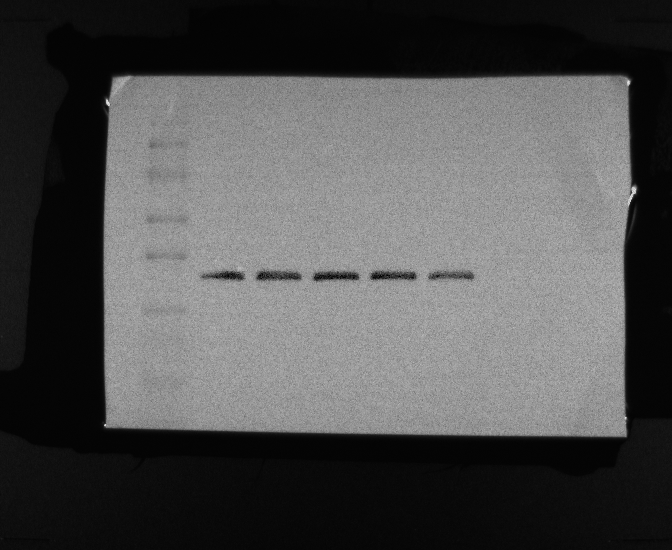

Supplement: Supplementary file 1 [file biomolecules-16-00926-s001.zip › biomolecules-4345458-WB/WB/Angiogenesis/VEGF/membrane4/VEGF_WB_20250807_170817_00.02.000_2_8bit.tif]

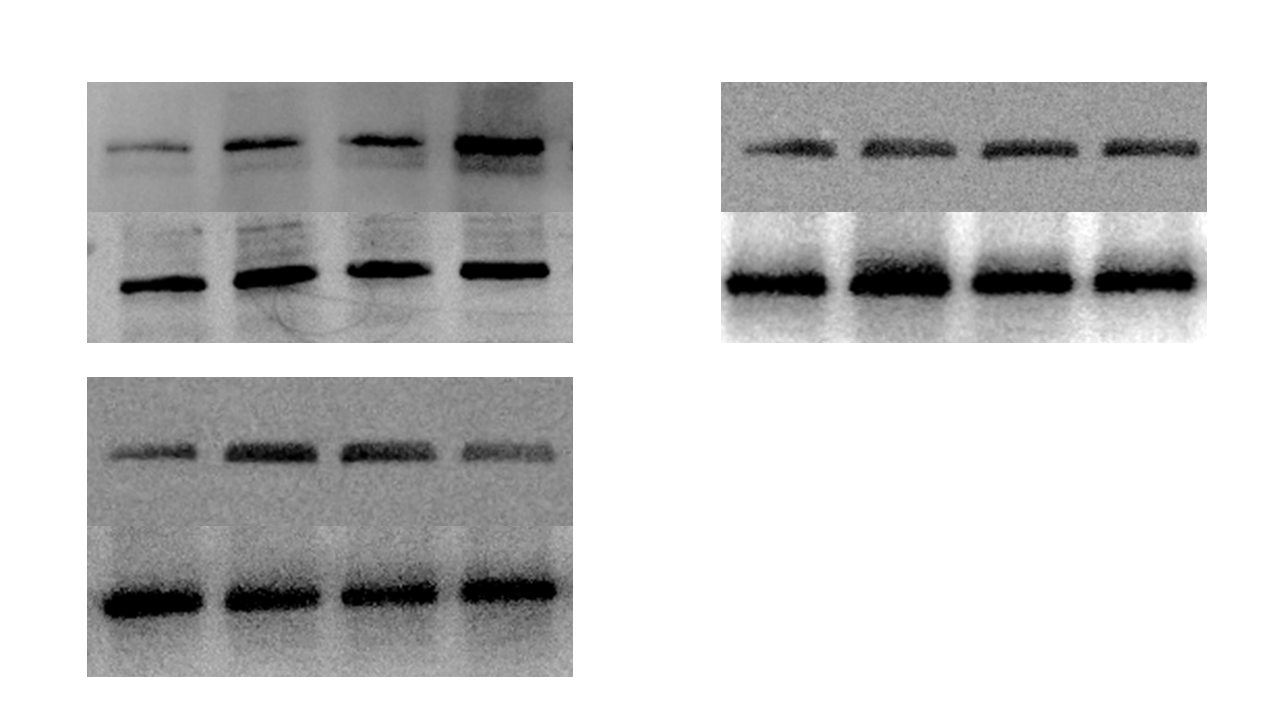

Supplement: Supplementary file 1 [file biomolecules-16-00926-s001.zip › biomolecules-4345458-WB/WB/Angiogenesis/VEGF/WB.tif]

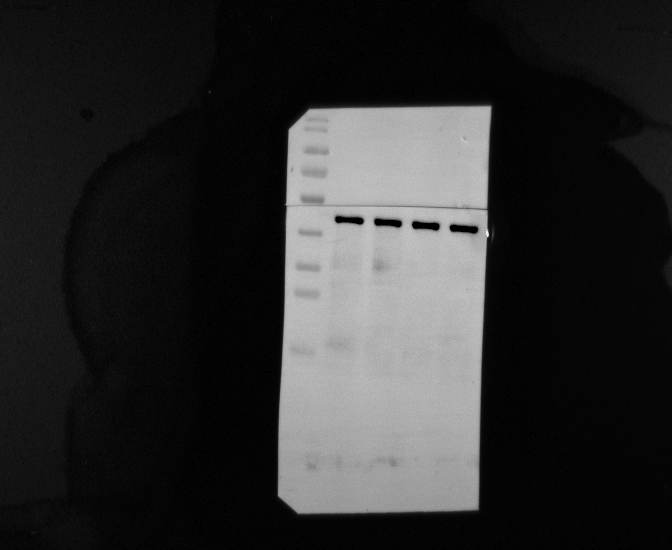

Supplement: Supplementary file 1 [file biomolecules-16-00926-s001.zip › biomolecules-4345458-WB/WB/Antioxidant/Caspase 1/membrane1/B-actin/Caspase1-1-All_8bit.tif]

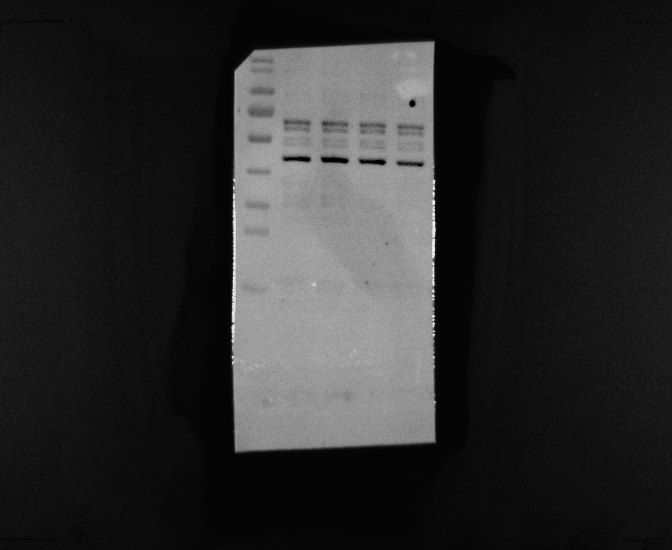

Supplement: Supplementary file 1 [file biomolecules-16-00926-s001.zip › biomolecules-4345458-WB/WB/Antioxidant/Caspase 1/membrane1/Caspase1-1-Overlay _8bit.tif]

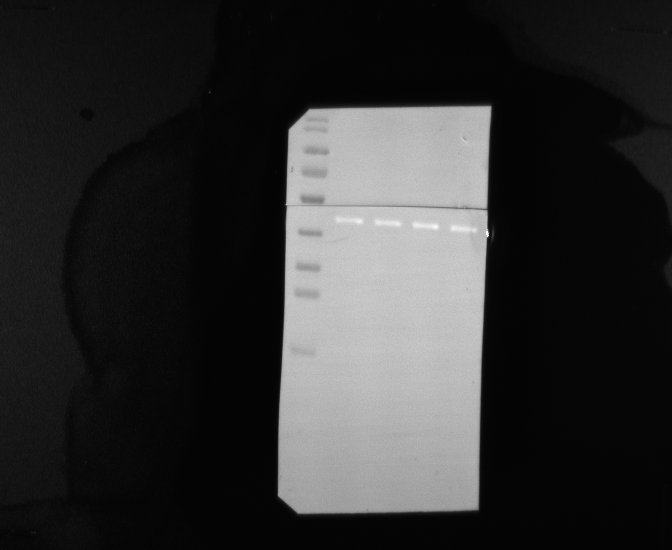

Supplement: Supplementary file 1 [file biomolecules-16-00926-s001.zip › biomolecules-4345458-WB/WB/Antioxidant/Caspase 1/membrane1/Caspase1-1-white-light_8bit.tif]

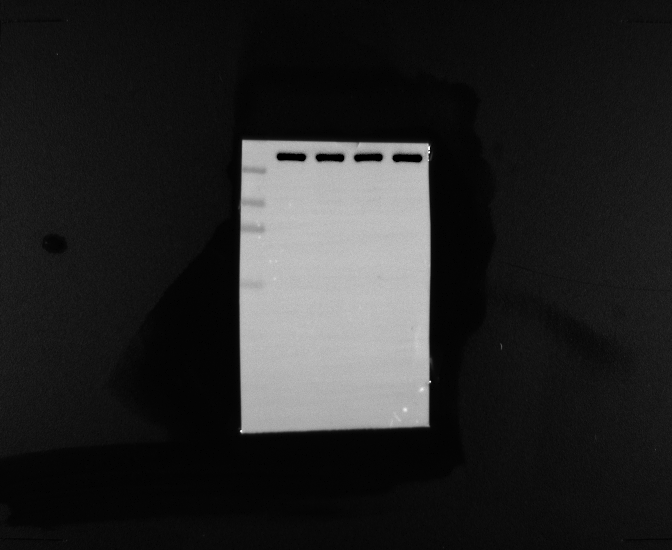

Supplement: Supplementary file 1 [file biomolecules-16-00926-s001.zip › biomolecules-4345458-WB/WB/Antioxidant/Caspase 1/membrane2/b-actin/Caspase1-2-Actin_8bit.tif]

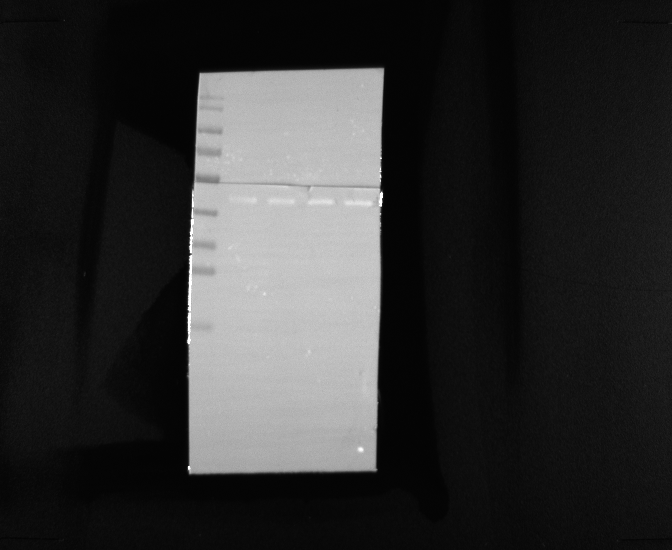

Supplement: Supplementary file 1 [file biomolecules-16-00926-s001.zip › biomolecules-4345458-WB/WB/Antioxidant/Caspase 1/membrane2/Caspase1-2-All-white-light_8bit.tif]

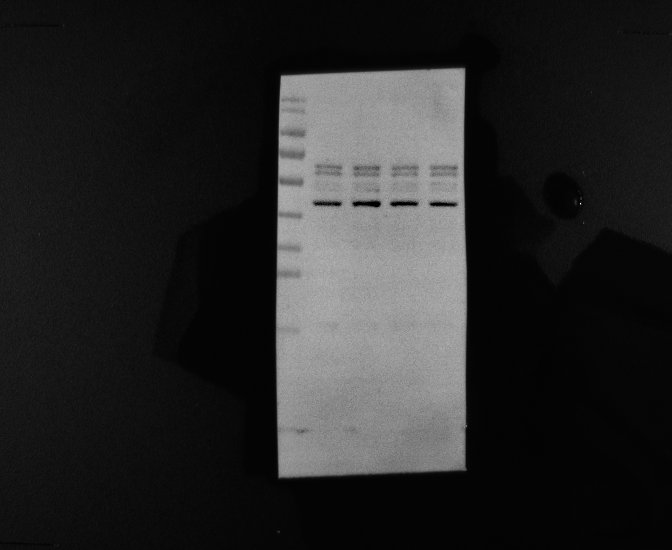

Supplement: Supplementary file 1 [file biomolecules-16-00926-s001.zip › biomolecules-4345458-WB/WB/Antioxidant/Caspase 1/membrane2/Caspase1-2-Overlay_8bit.tif]

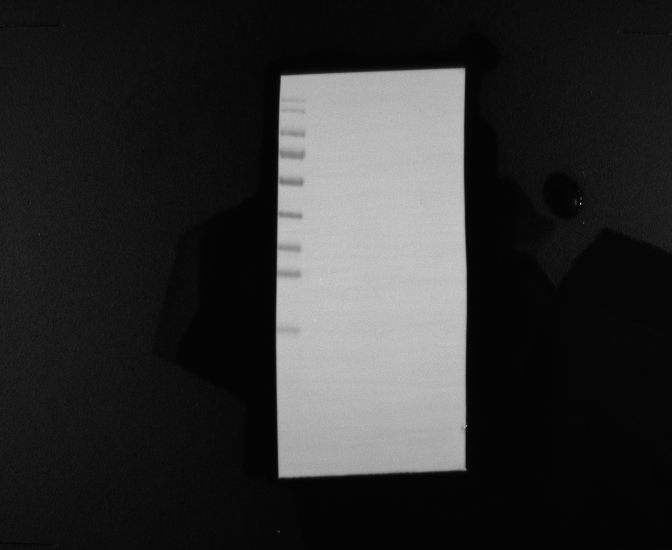

Supplement: Supplementary file 1 [file biomolecules-16-00926-s001.zip › biomolecules-4345458-WB/WB/Antioxidant/Caspase 1/membrane2/Caspase1-2-white-light_8bit.tif]

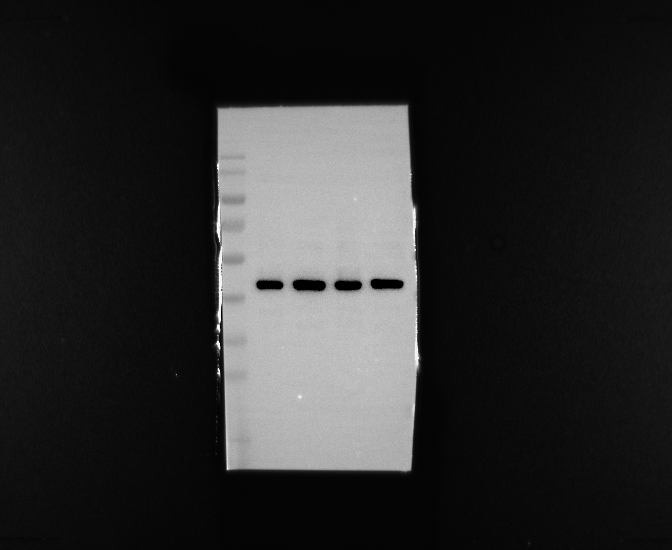

Supplement: Supplementary file 1 [file biomolecules-16-00926-s001.zip › biomolecules-4345458-WB/WB/Antioxidant/Caspase 1/membrane3/actin/β-Actin(Caspase1-3)_8bit.tif]

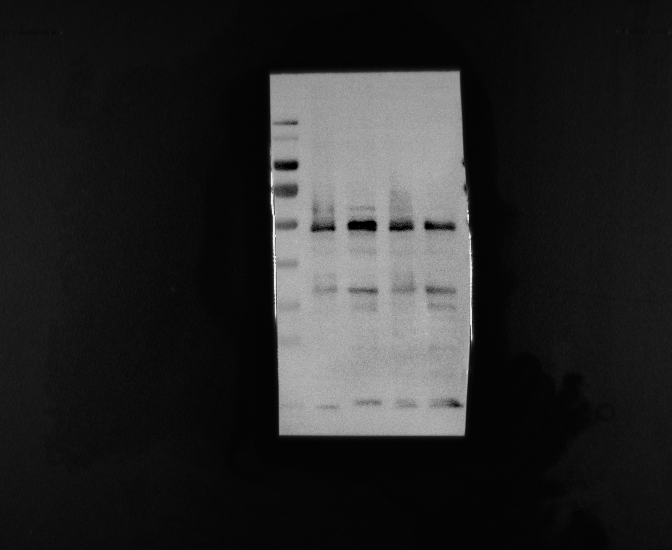

Supplement: Supplementary file 1 [file biomolecules-16-00926-s001.zip › biomolecules-4345458-WB/WB/Antioxidant/Caspase 1/membrane3/Caspase1-3-2.tif]

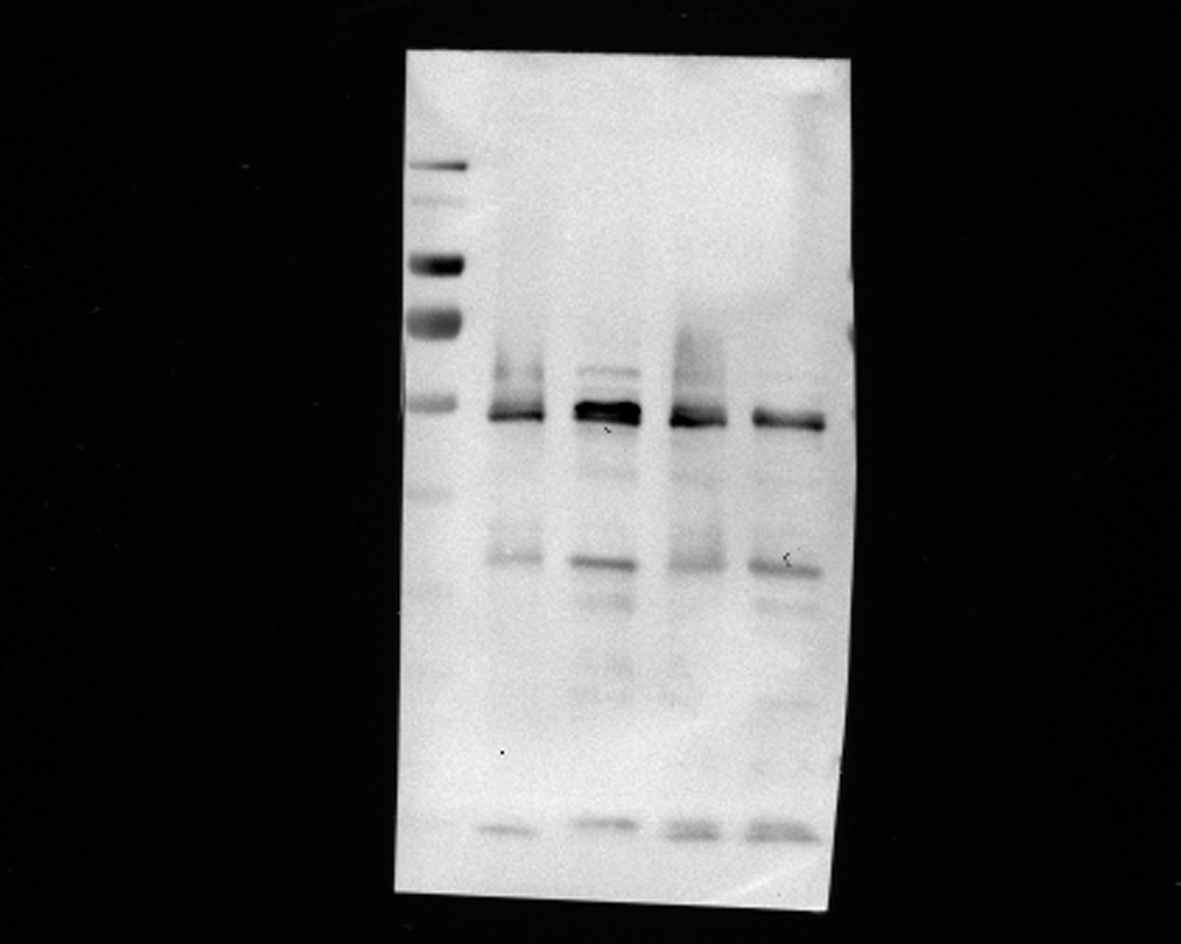

Supplement: Supplementary file 1 [file biomolecules-16-00926-s001.zip › biomolecules-4345458-WB/WB/Antioxidant/Caspase 1/membrane3/Caspase1-3.tif]

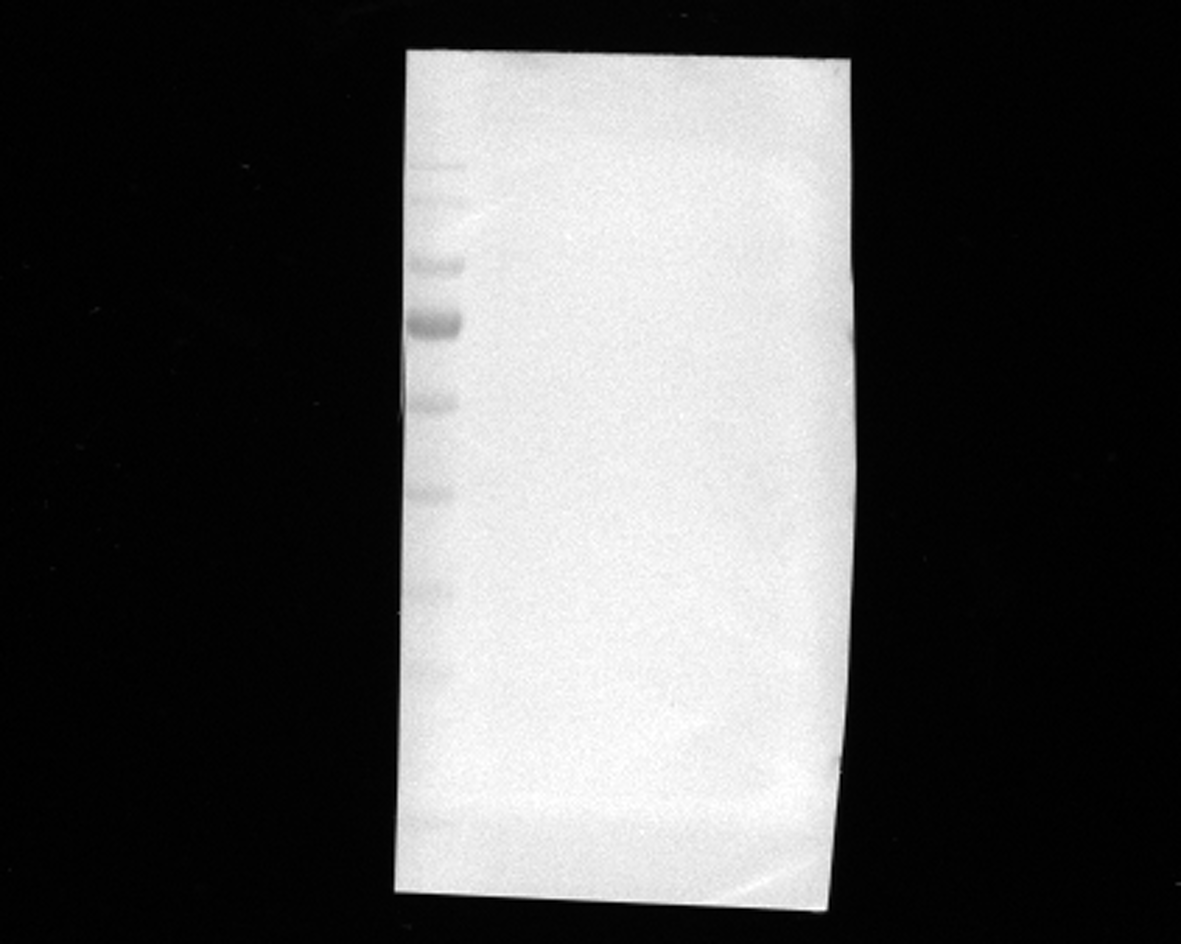

Supplement: Supplementary file 1 [file biomolecules-16-00926-s001.zip › biomolecules-4345458-WB/WB/Antioxidant/Caspase 1/membrane3/Caspase1-BF.tif]

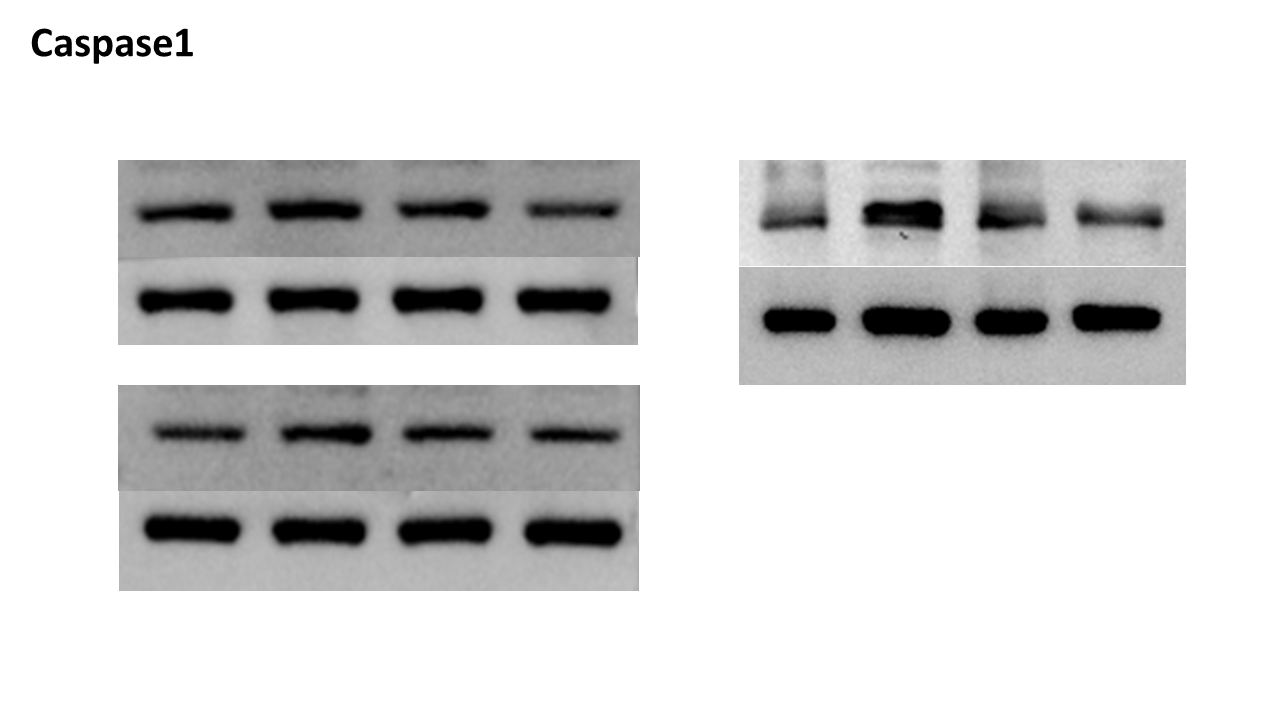

Supplement: Supplementary file 1 [file biomolecules-16-00926-s001.zip › biomolecules-4345458-WB/WB/Antioxidant/Caspase 1/WB.tif]

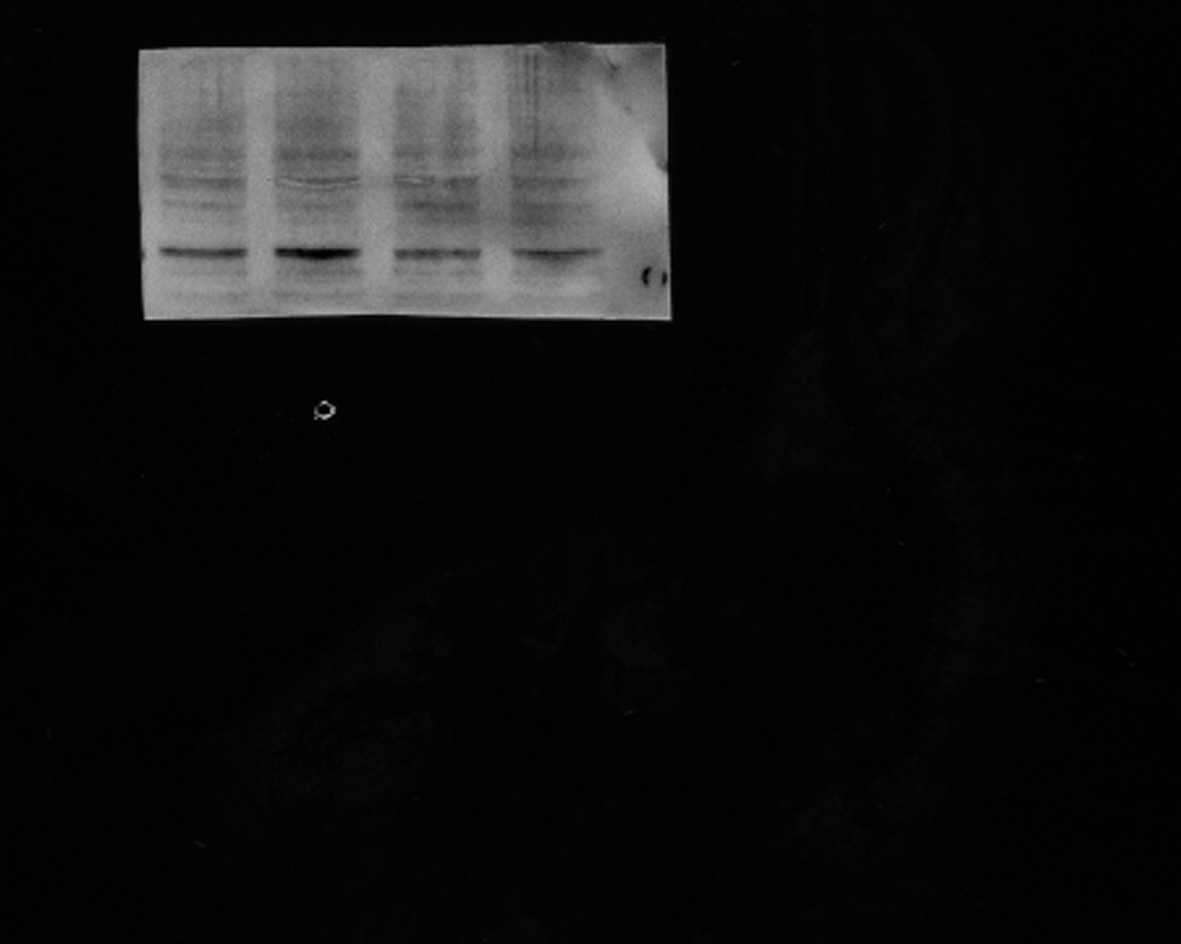

Supplement: Supplementary file 1 [file biomolecules-16-00926-s001.zip › biomolecules-4345458-WB/WB/Antioxidant/COX2/membrane 1/COX2 (1).tif]

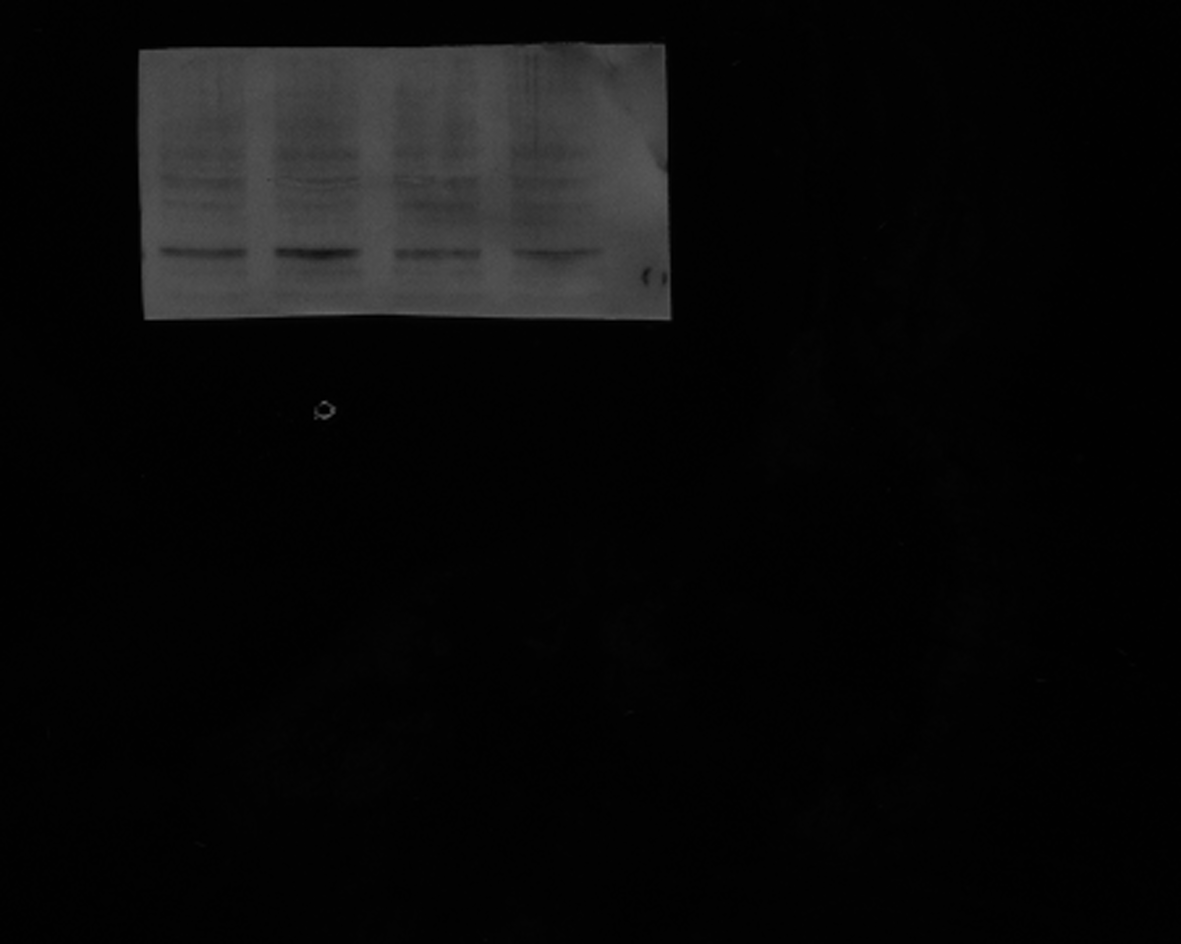

Supplement: Supplementary file 1 [file biomolecules-16-00926-s001.zip › biomolecules-4345458-WB/WB/Antioxidant/COX2/membrane 1/COX2 (3).tif]

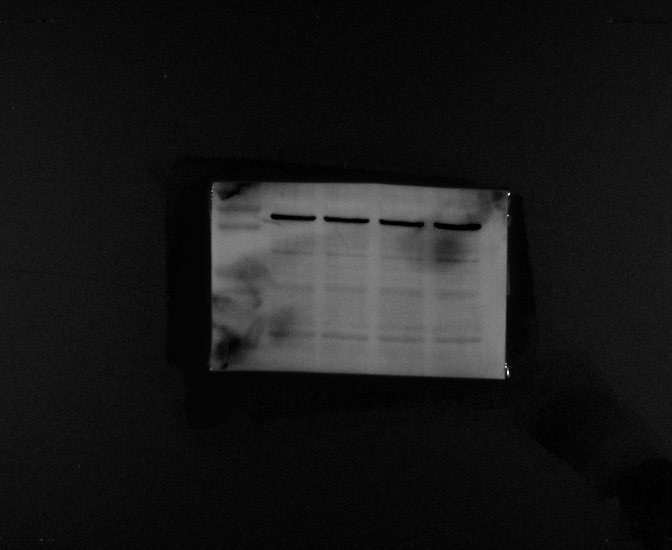

Supplement: Supplementary file 1 [file biomolecules-16-00926-s001.zip › biomolecules-4345458-WB/WB/Antioxidant/COX2/membrane 1/GAPDH.tif]

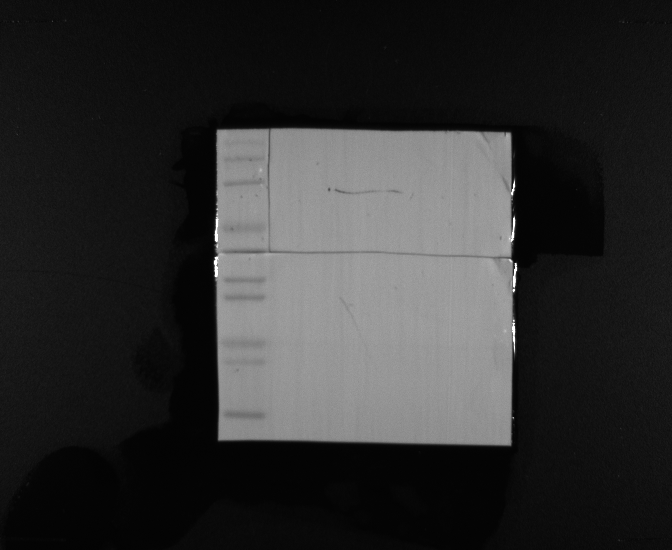

Supplement: Supplementary file 1 [file biomolecules-16-00926-s001.zip › biomolecules-4345458-WB/WB/Antioxidant/COX2/membrane 1/total3_bf_8bit_8bit_8bit.tif]

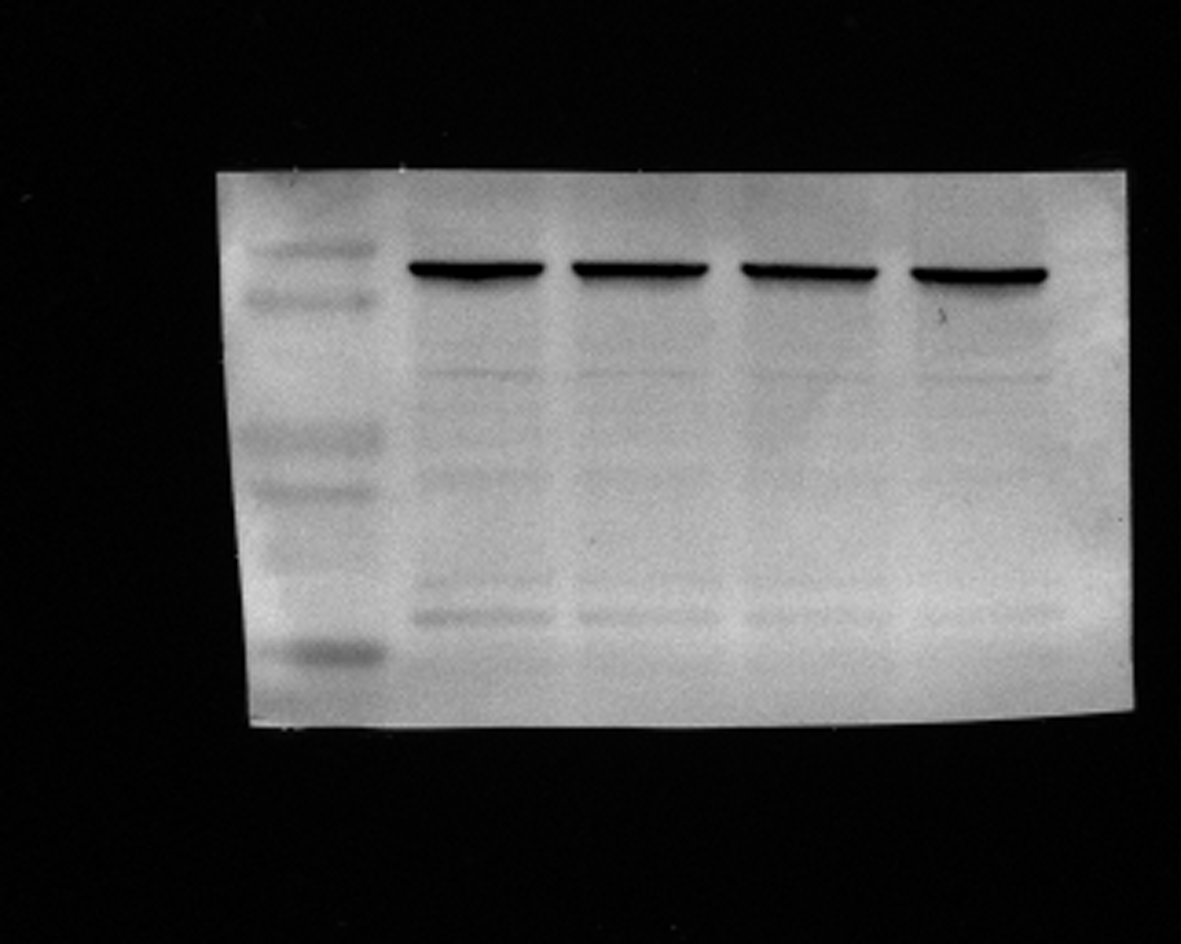

Supplement: Supplementary file 1 [file biomolecules-16-00926-s001.zip › biomolecules-4345458-WB/WB/Antioxidant/COX2/membrane 2/CHEMI_09302025_184734_(Chemi).tif]

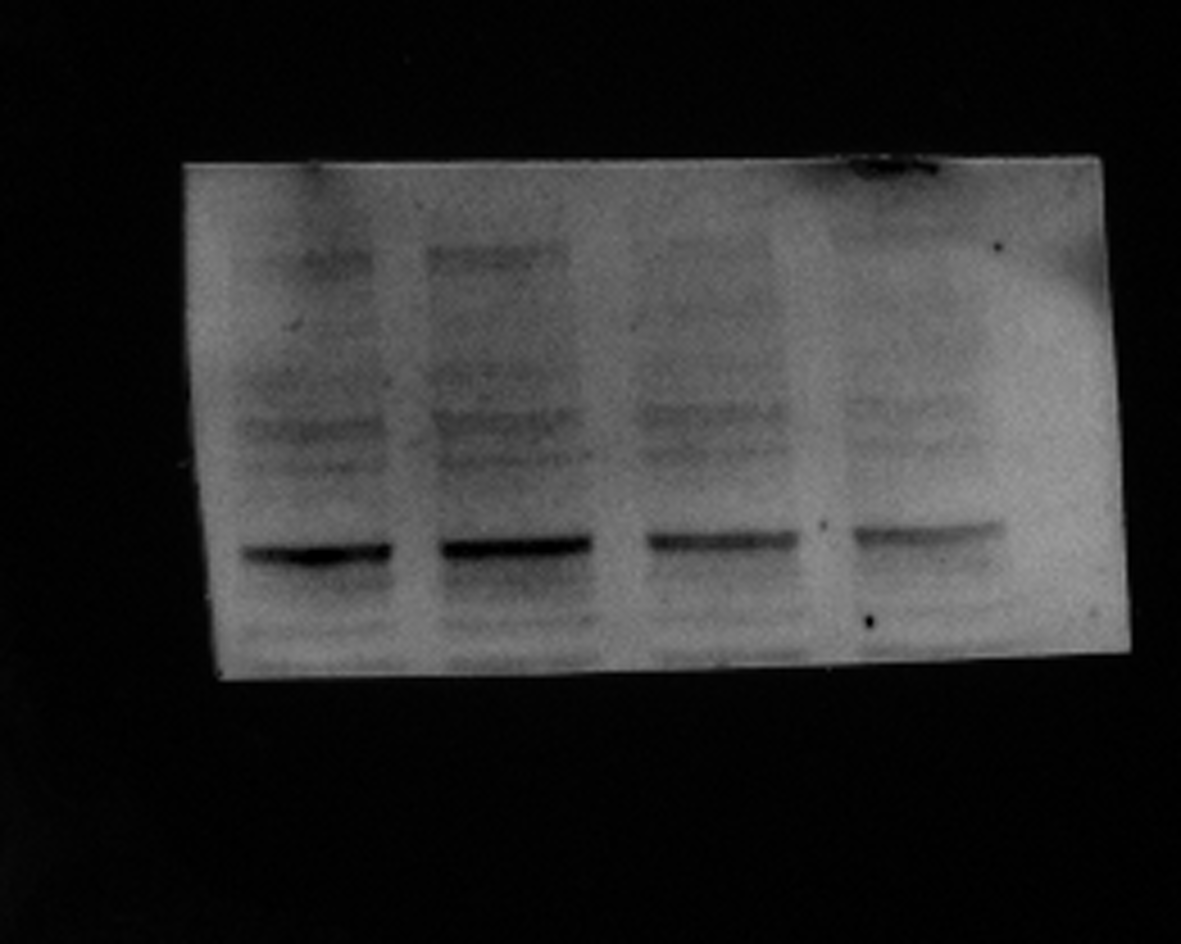

Supplement: Supplementary file 1 [file biomolecules-16-00926-s001.zip › biomolecules-4345458-WB/WB/Antioxidant/COX2/membrane 2/CHEMI_09302025_220809_(Chemi).tif]

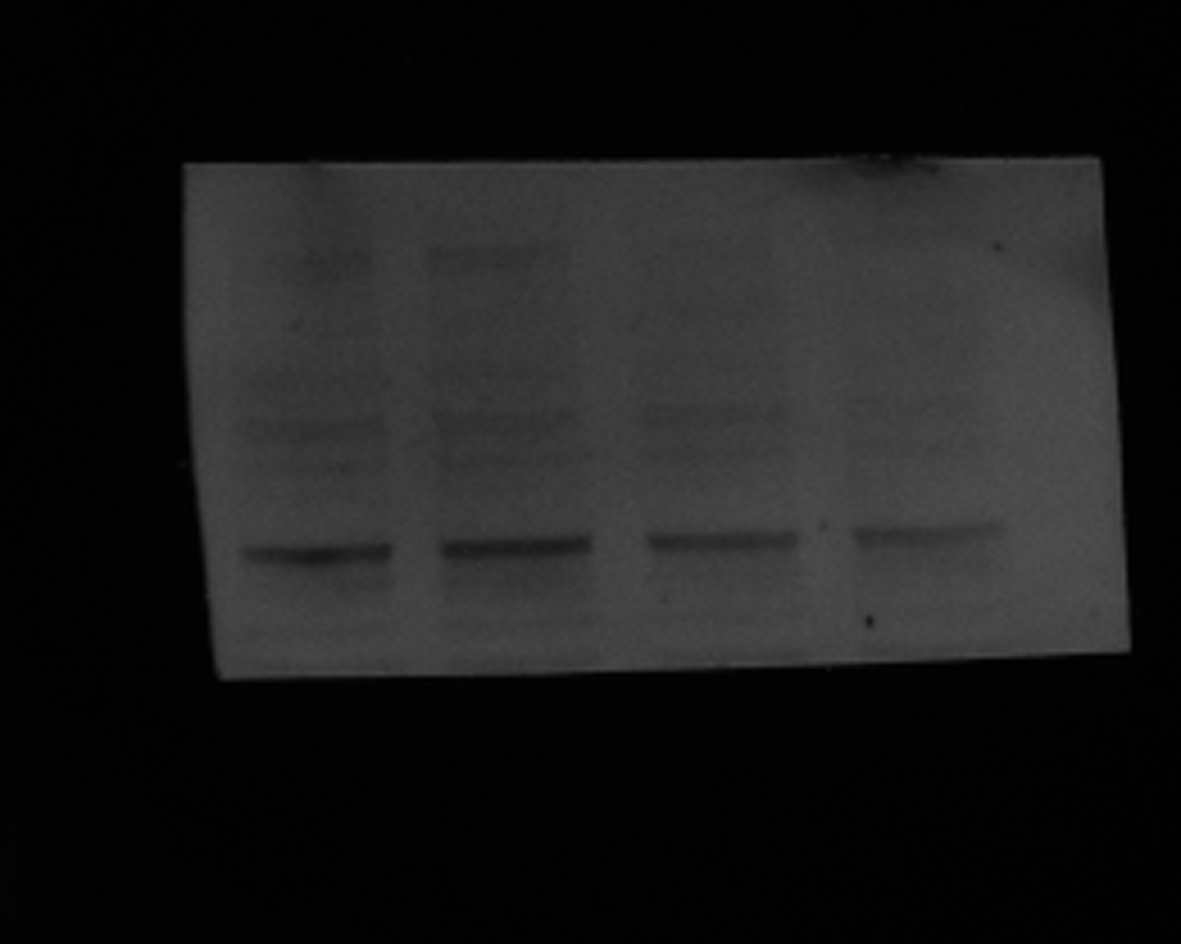

Supplement: Supplementary file 1 [file biomolecules-16-00926-s001.zip › biomolecules-4345458-WB/WB/Antioxidant/COX2/membrane 2/CHEMI_09302025_220809_(Overlay).tif]

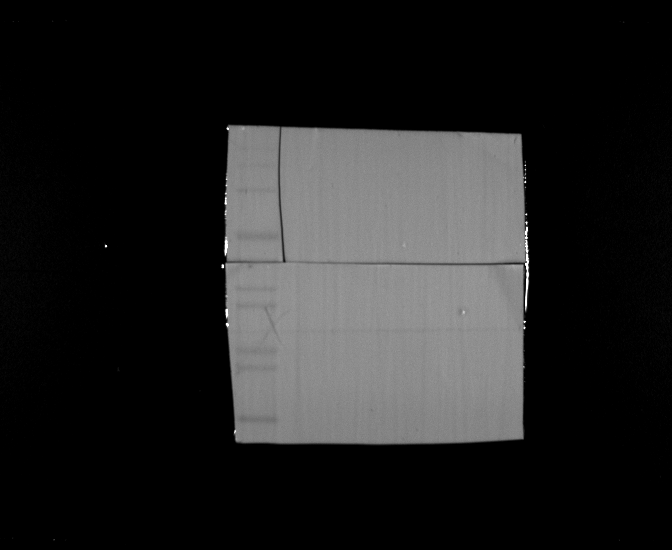

Supplement: Supplementary file 1 [file biomolecules-16-00926-s001.zip › biomolecules-4345458-WB/WB/Antioxidant/COX2/membrane 2/total1_bf_8bit.tif]

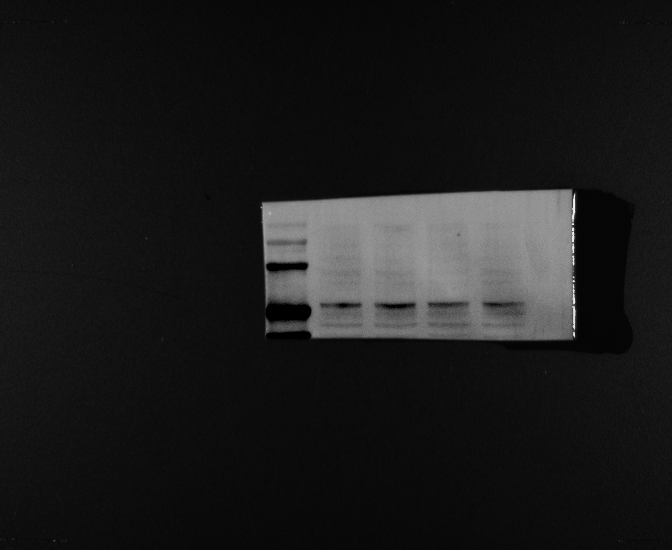

Supplement: Supplementary file 1 [file biomolecules-16-00926-s001.zip › biomolecules-4345458-WB/WB/Antioxidant/COX2/membrane 3/COX2-3.tif]

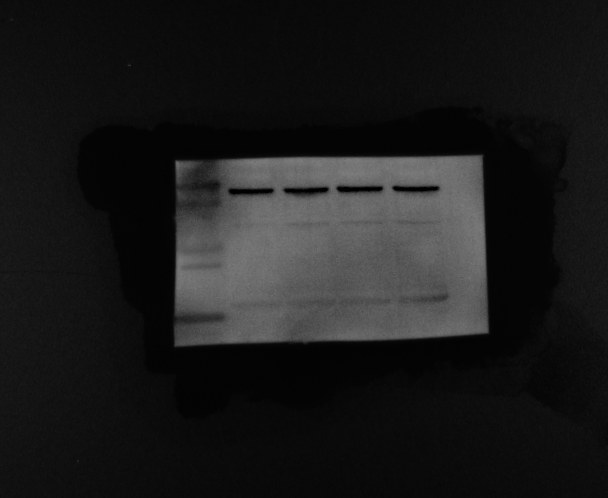

Supplement: Supplementary file 1 [file biomolecules-16-00926-s001.zip › biomolecules-4345458-WB/WB/Antioxidant/COX2/membrane 3/membrane 2_8bit.tif]

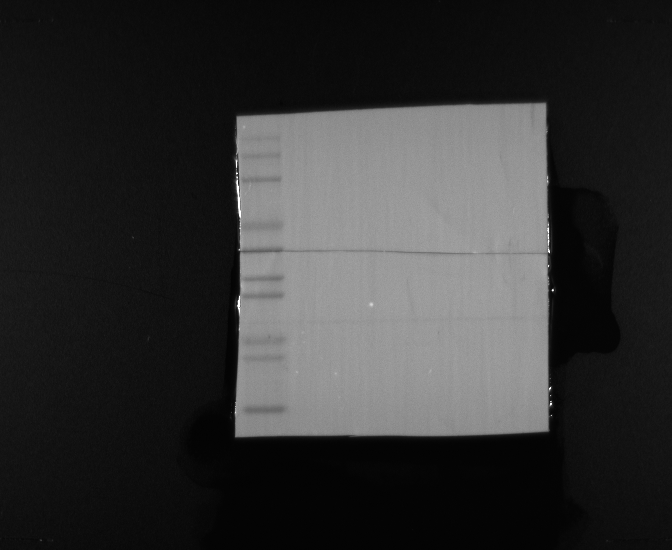

Supplement: Supplementary file 1 [file biomolecules-16-00926-s001.zip › biomolecules-4345458-WB/WB/Antioxidant/COX2/membrane 3/total2_bf_8bit_8bit.tif]

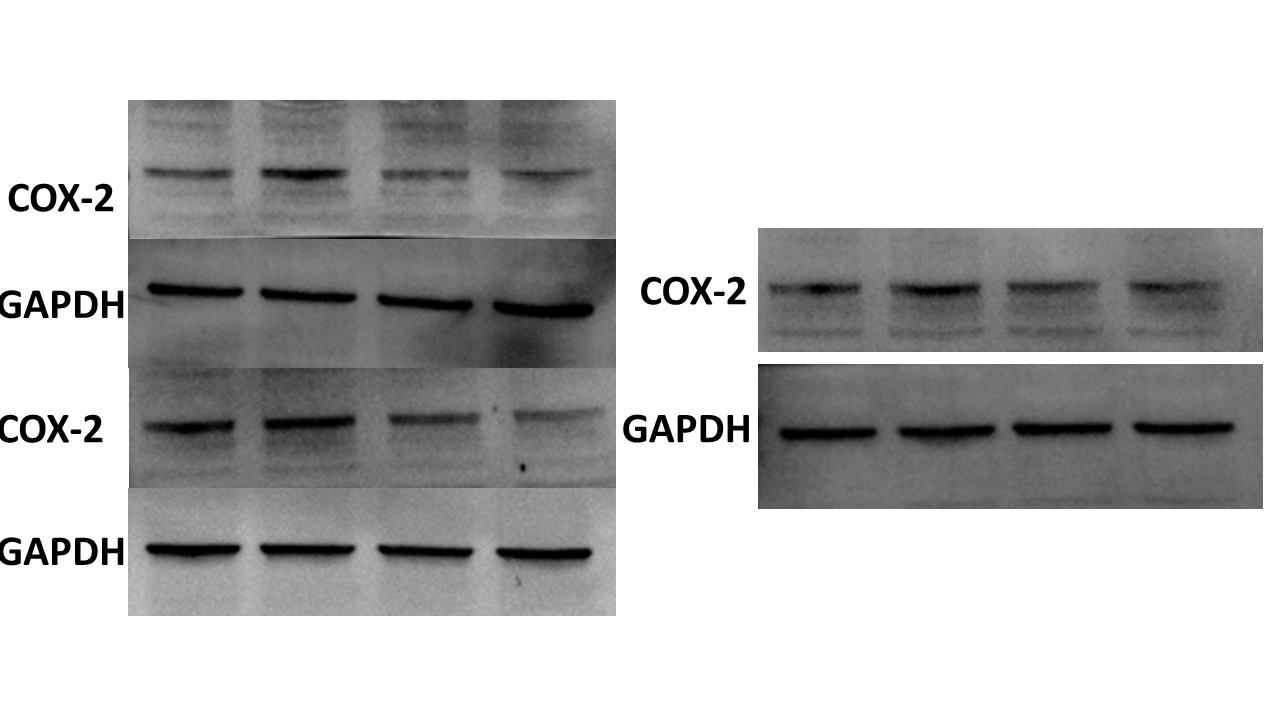

Supplement: Supplementary file 1 [file biomolecules-16-00926-s001.zip › biomolecules-4345458-WB/WB/Antioxidant/COX2/WB.tif]

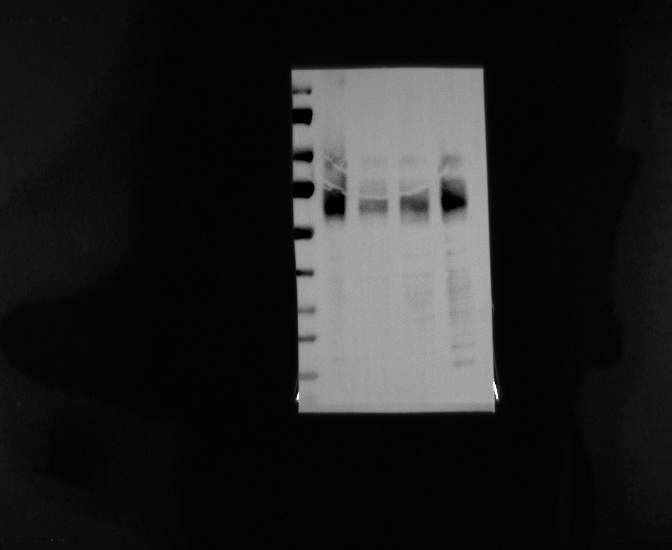

Supplement: Supplementary file 1 [file biomolecules-16-00926-s001.zip › biomolecules-4345458-WB/WB/Antioxidant/KEAP-1/keap1/1/WB_20251031_185152_00.00.225_8bit.tif]

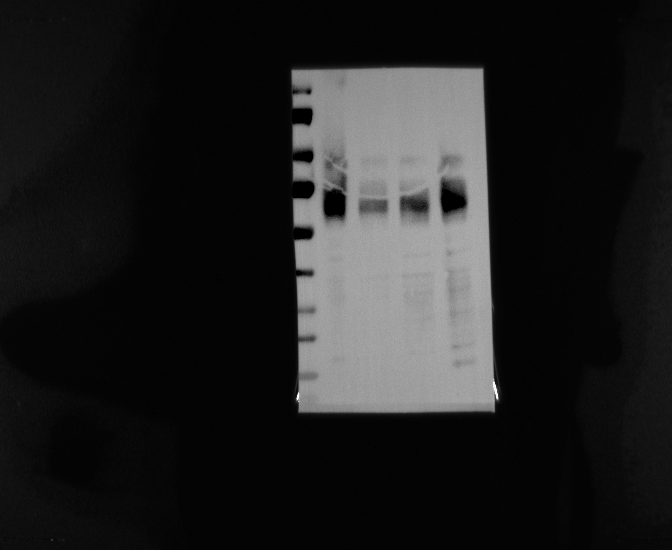

Supplement: Supplementary file 1 [file biomolecules-16-00926-s001.zip › biomolecules-4345458-WB/WB/Antioxidant/KEAP-1/keap1/1/WB_20251031_185152_00.00_8bit.tif]

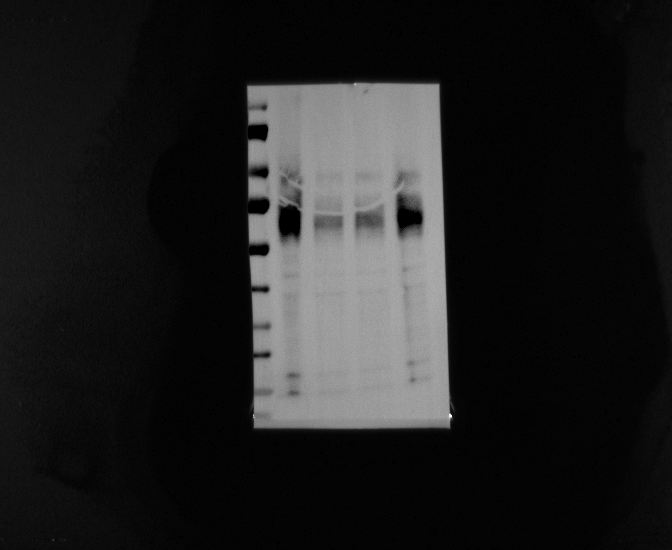

Supplement: Supplementary file 1 [file biomolecules-16-00926-s001.zip › biomolecules-4345458-WB/WB/Antioxidant/KEAP-1/keap1/1/WB_20251031_185444_00.00_8bit.tif]

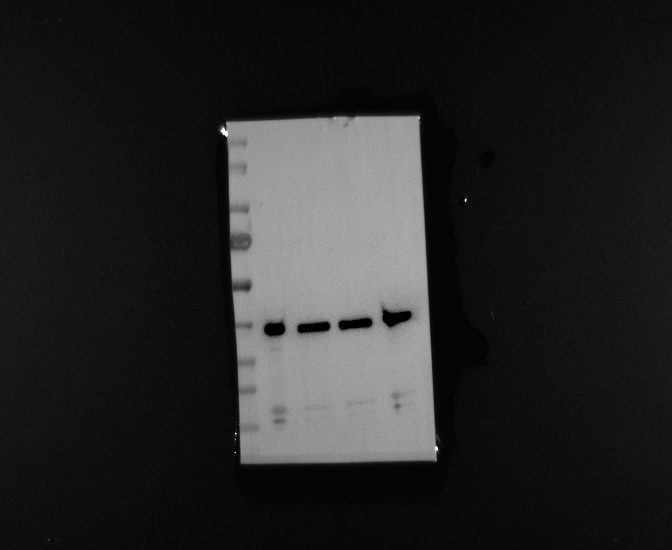

Supplement: Supplementary file 1 [file biomolecules-16-00926-s001.zip › biomolecules-4345458-WB/WB/Antioxidant/KEAP-1/keap1/1/WB_20251101_203707_00.00_8bit.tif]

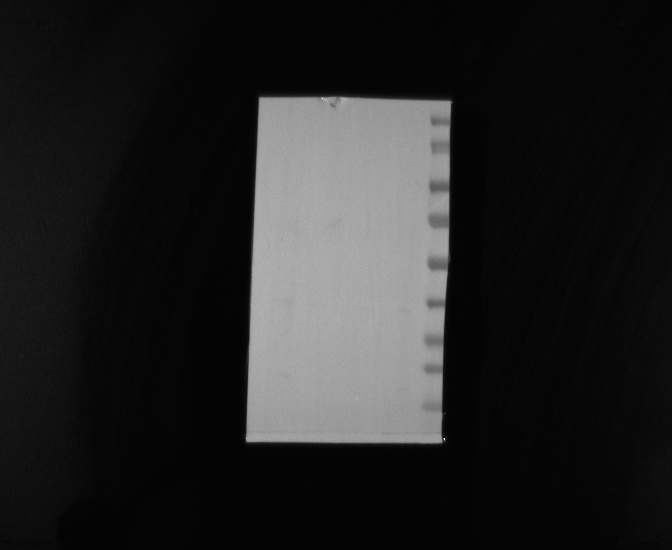

Supplement: Supplementary file 1 [file biomolecules-16-00926-s001.zip › biomolecules-4345458-WB/WB/Antioxidant/KEAP-1/keap1/1/WB_20251101_204530_00.01_8bit.tif]

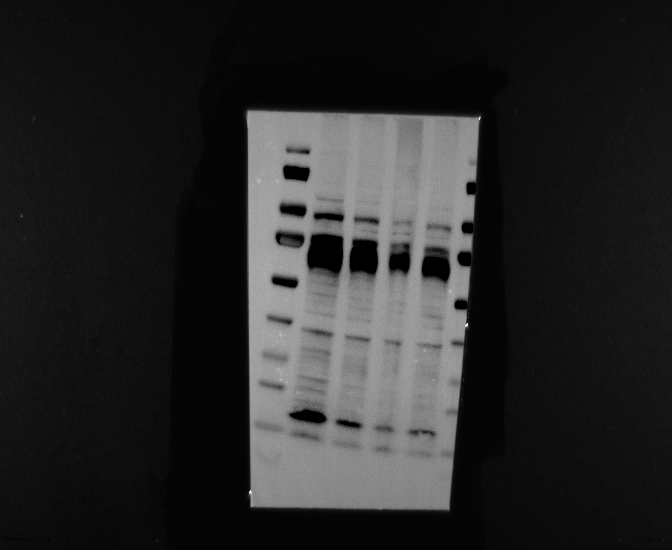

Supplement: Supplementary file 1 [file biomolecules-16-00926-s001.zip › biomolecules-4345458-WB/WB/Antioxidant/KEAP-1/keap1/2/WB_20251029_162618_00.00_8bit.tif]

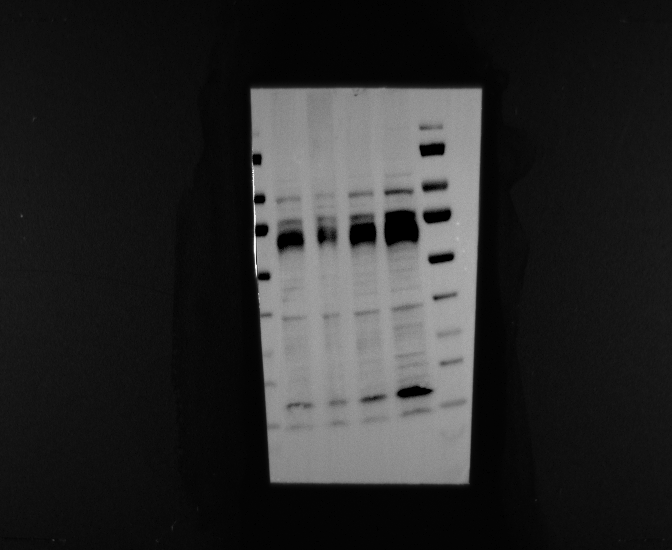

Supplement: Supplementary file 1 [file biomolecules-16-00926-s001.zip › biomolecules-4345458-WB/WB/Antioxidant/KEAP-1/keap1/2/WB_20251029_162901_00.00.108_8bit.tif]

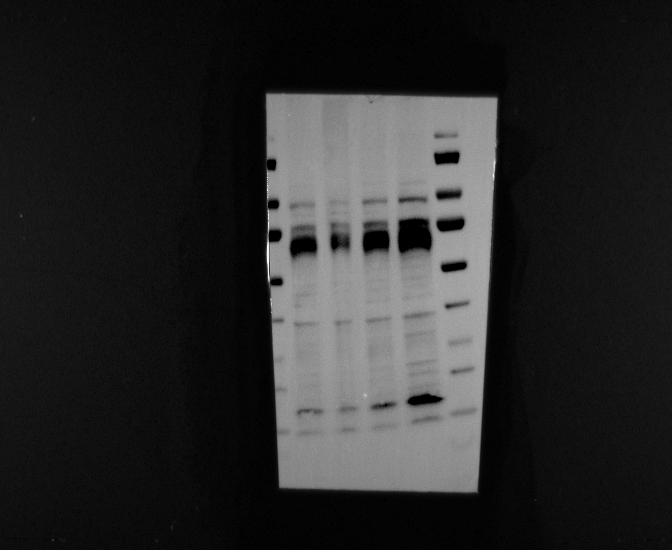

Supplement: Supplementary file 1 [file biomolecules-16-00926-s001.zip › biomolecules-4345458-WB/WB/Antioxidant/KEAP-1/keap1/2/WB_20251029_163132_00.00_8bit.tif]

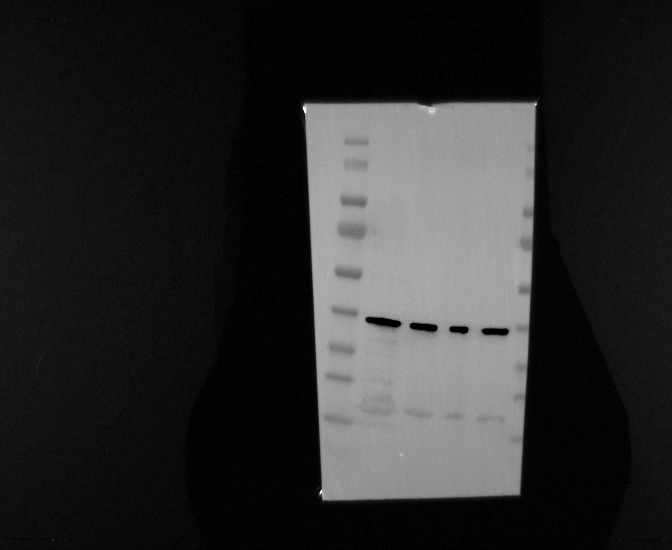

Supplement: Supplementary file 1 [file biomolecules-16-00926-s001.zip › biomolecules-4345458-WB/WB/Antioxidant/KEAP-1/keap1/2/WB_20251030_155137_00.00_8bit.tif]

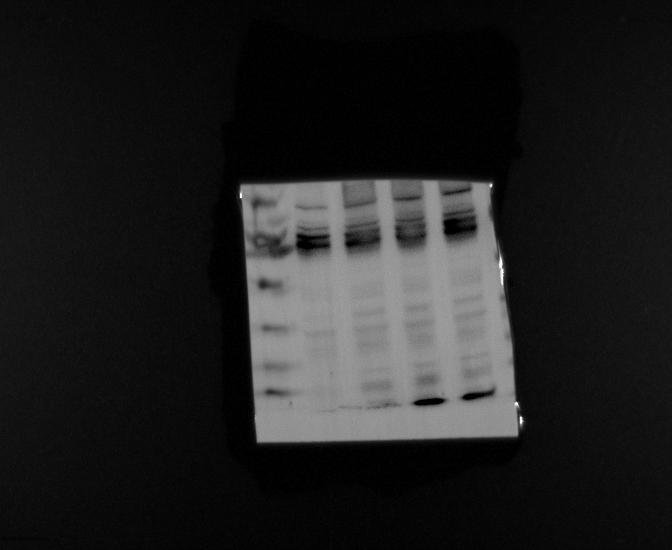

Supplement: Supplementary file 1 [file biomolecules-16-00926-s001.zip › biomolecules-4345458-WB/WB/Antioxidant/KEAP-1/keap1/3/WB_20251020_123634_00.00_8bit.tif]

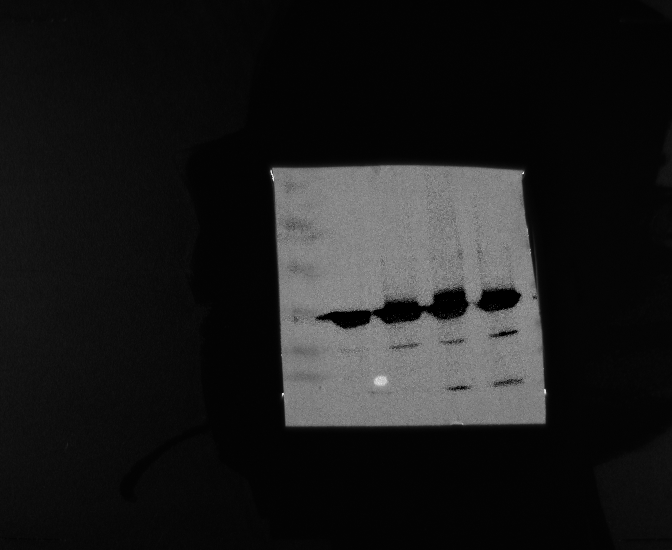

Supplement: Supplementary file 1 [file biomolecules-16-00926-s001.zip › biomolecules-4345458-WB/WB/Antioxidant/KEAP-1/keap1/3/WB_20251028_105529_00.00_8bit.tif]

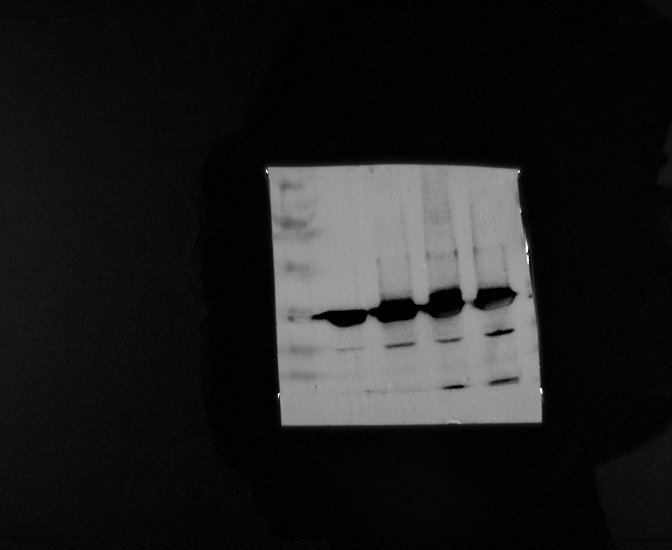

Supplement: Supplementary file 1 [file biomolecules-16-00926-s001.zip › biomolecules-4345458-WB/WB/Antioxidant/KEAP-1/keap1/3/WB_20251028_105554_00.00.011_8bit.tif]

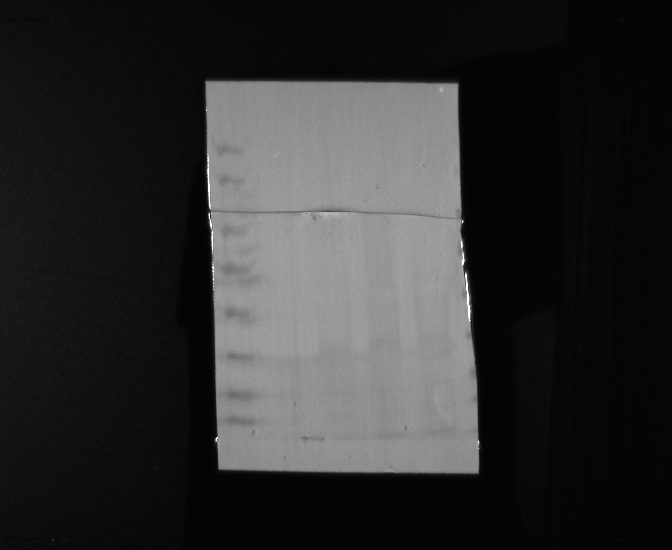

Supplement: Supplementary file 1 [file biomolecules-16-00926-s001.zip › biomolecules-4345458-WB/WB/Antioxidant/KEAP-1/keap1/3/WB_20251028_110028_00.01_8bit.tif]

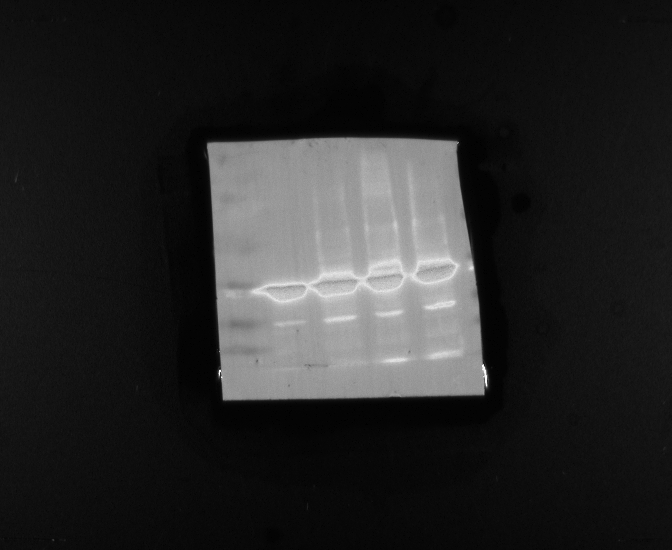

Supplement: Supplementary file 1 [file biomolecules-16-00926-s001.zip › biomolecules-4345458-WB/WB/Antioxidant/KEAP-1/keap1/3/WB_20251028_123744_00.00_8bit(0).tif]

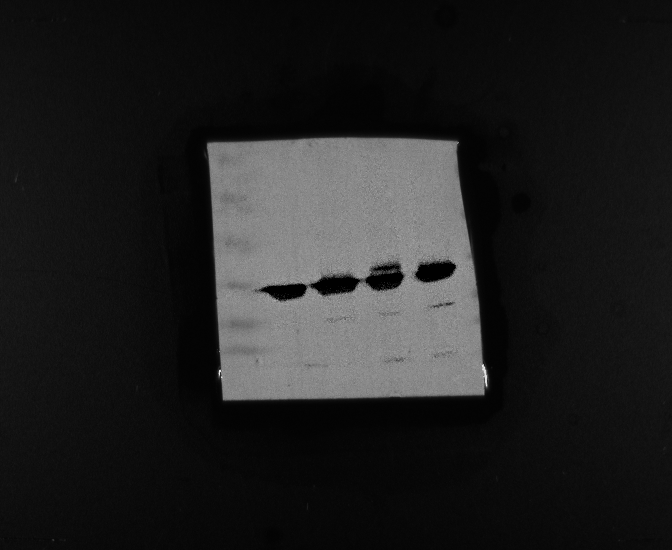

Supplement: Supplementary file 1 [file biomolecules-16-00926-s001.zip › biomolecules-4345458-WB/WB/Antioxidant/KEAP-1/keap1/3/WB_20251028_123744_00.00_8bit.tif]

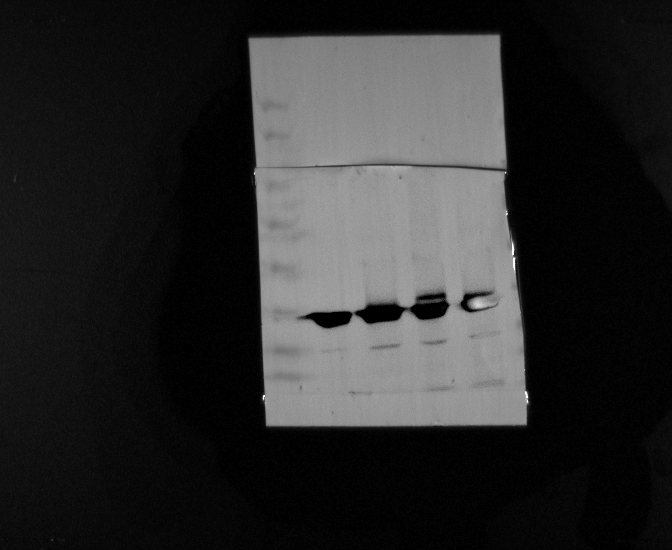

Supplement: Supplementary file 1 [file biomolecules-16-00926-s001.zip › biomolecules-4345458-WB/WB/Antioxidant/KEAP-1/keap1/3/WB_20251028_124253_00.00_8bit.tif]

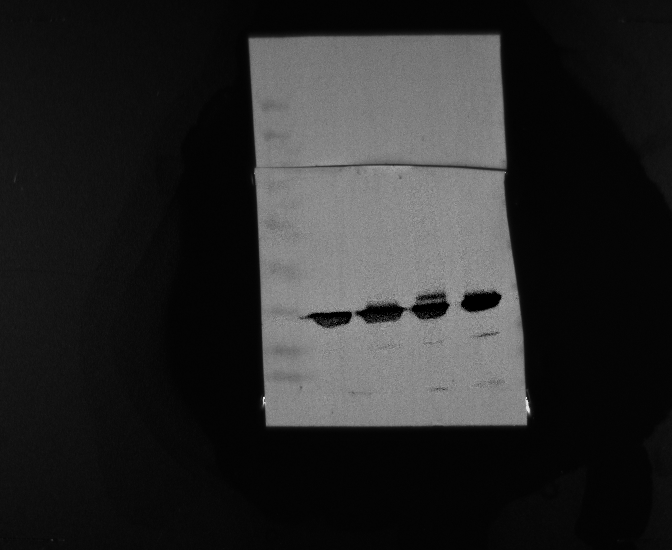

Supplement: Supplementary file 1 [file biomolecules-16-00926-s001.zip › biomolecules-4345458-WB/WB/Antioxidant/KEAP-1/keap1/3/WB_20251028_124320_00.00_8bit.tif]

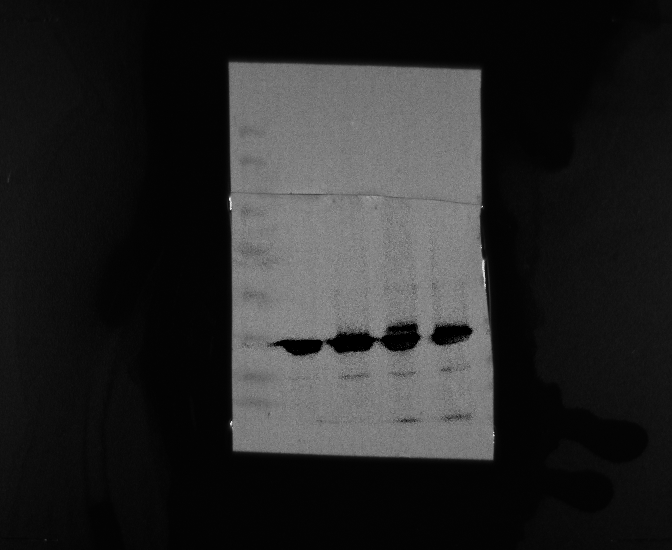

Supplement: Supplementary file 1 [file biomolecules-16-00926-s001.zip › biomolecules-4345458-WB/WB/Antioxidant/KEAP-1/keap1/3/WB_20251028_124828_00.00_8bit.tif]

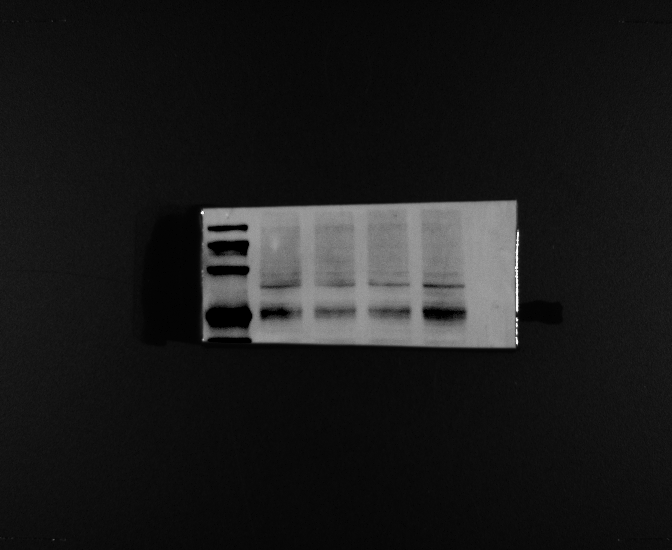

Supplement: Supplementary file 1 [file biomolecules-16-00926-s001.zip › biomolecules-4345458-WB/WB/Antioxidant/KEAP-1/membrane1/111_8bit.tif]

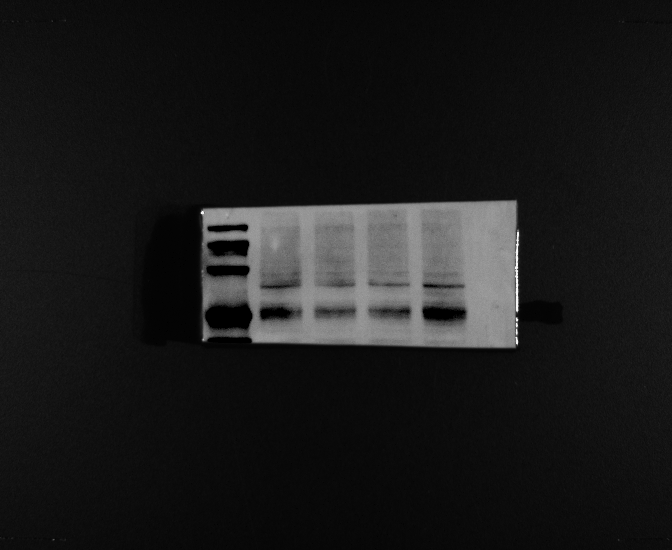

Supplement: Supplementary file 1 [file biomolecules-16-00926-s001.zip › biomolecules-4345458-WB/WB/Antioxidant/KEAP-1/membrane1/11_8bit.tif]

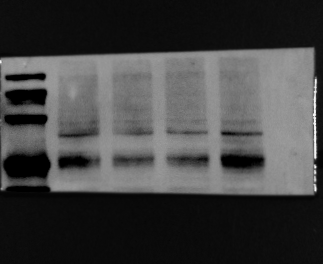

Supplement: Supplementary file 1 [file biomolecules-16-00926-s001.zip › biomolecules-4345458-WB/WB/Antioxidant/KEAP-1/membrane1/11_8bit1.tif]

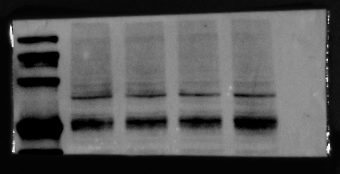

Supplement: Supplementary file 1 [file biomolecules-16-00926-s001.zip › biomolecules-4345458-WB/WB/Antioxidant/KEAP-1/membrane1/1_8b.tif]

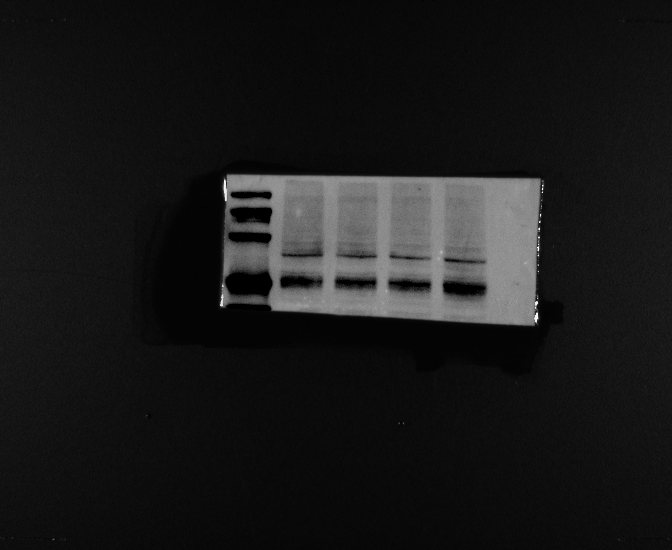

Supplement: Supplementary file 1 [file biomolecules-16-00926-s001.zip › biomolecules-4345458-WB/WB/Antioxidant/KEAP-1/membrane1/1_8bit.tif]

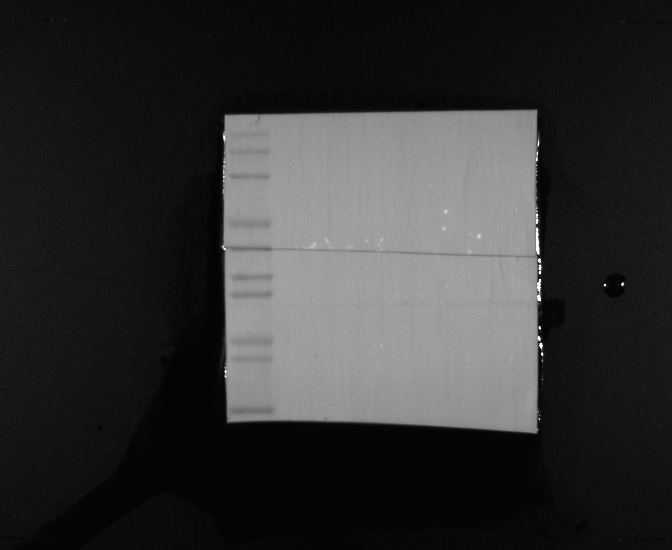

Supplement: Supplementary file 1 [file biomolecules-16-00926-s001.zip › biomolecules-4345458-WB/WB/Antioxidant/KEAP-1/membrane1/bright field_8bit.tif]

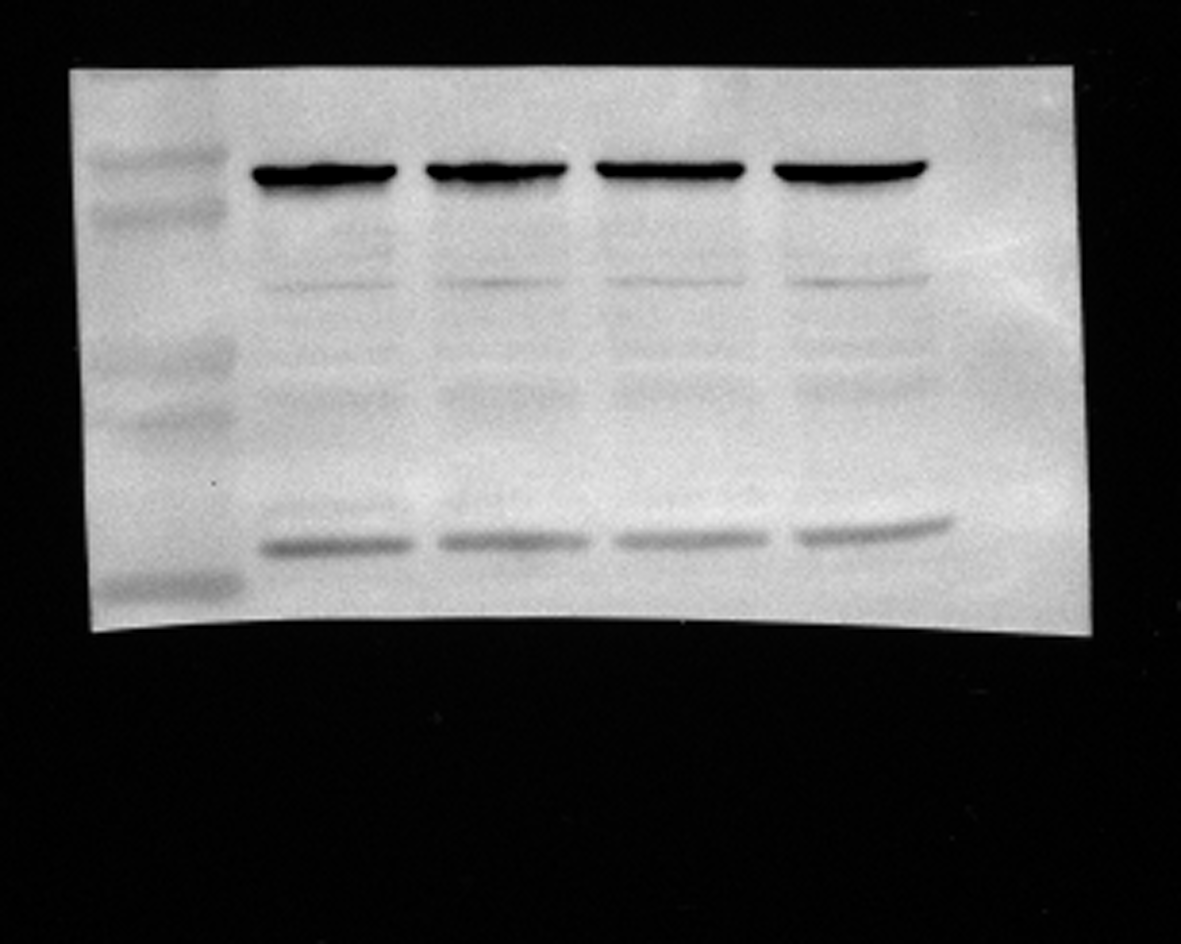

Supplement: Supplementary file 1 [file biomolecules-16-00926-s001.zip › biomolecules-4345458-WB/WB/Antioxidant/KEAP-1/membrane1/Gapdh/CHEMI_09302025_190346_(Chemi).tif]

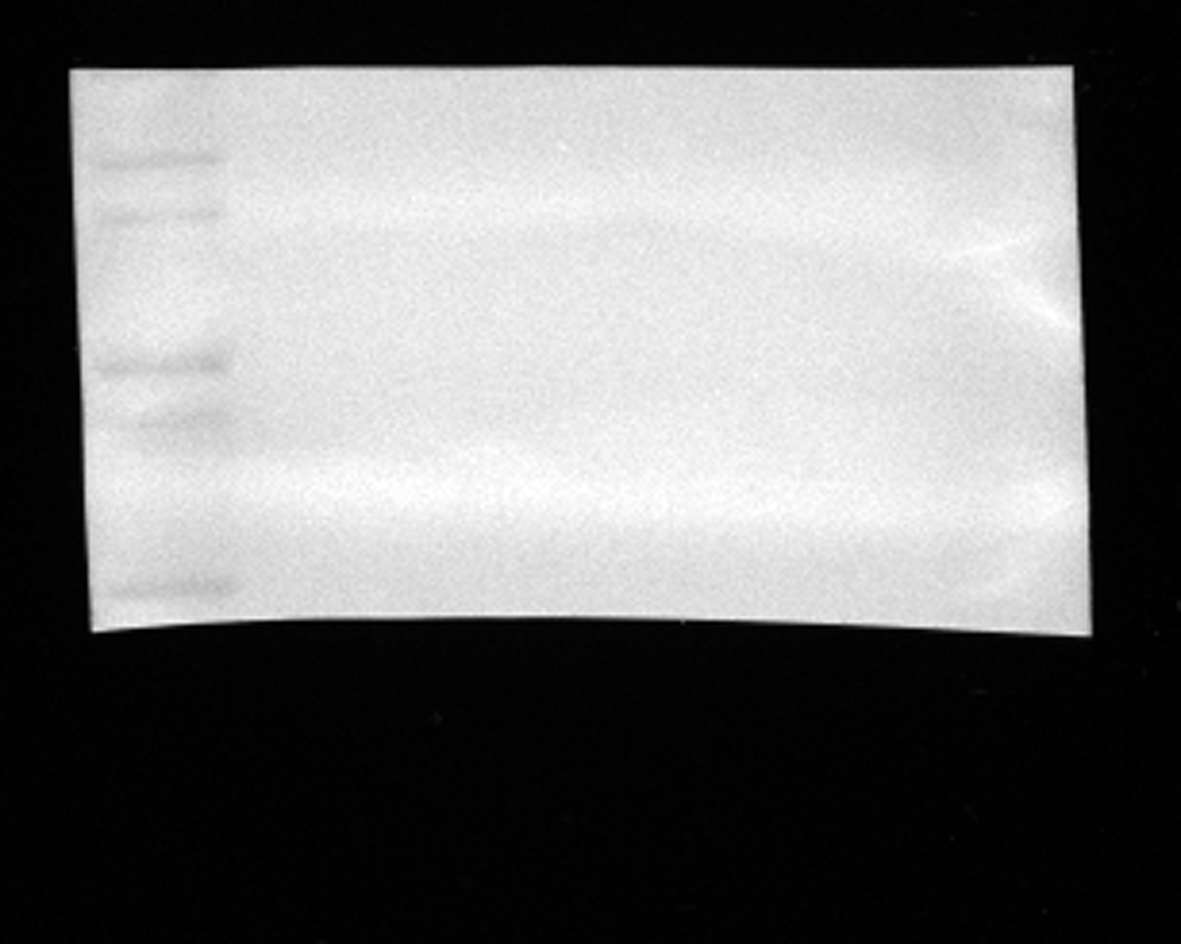

Supplement: Supplementary file 1 [file biomolecules-16-00926-s001.zip › biomolecules-4345458-WB/WB/Antioxidant/KEAP-1/membrane1/Gapdh/CHEMI_09302025_190346_(Membrane).tif]

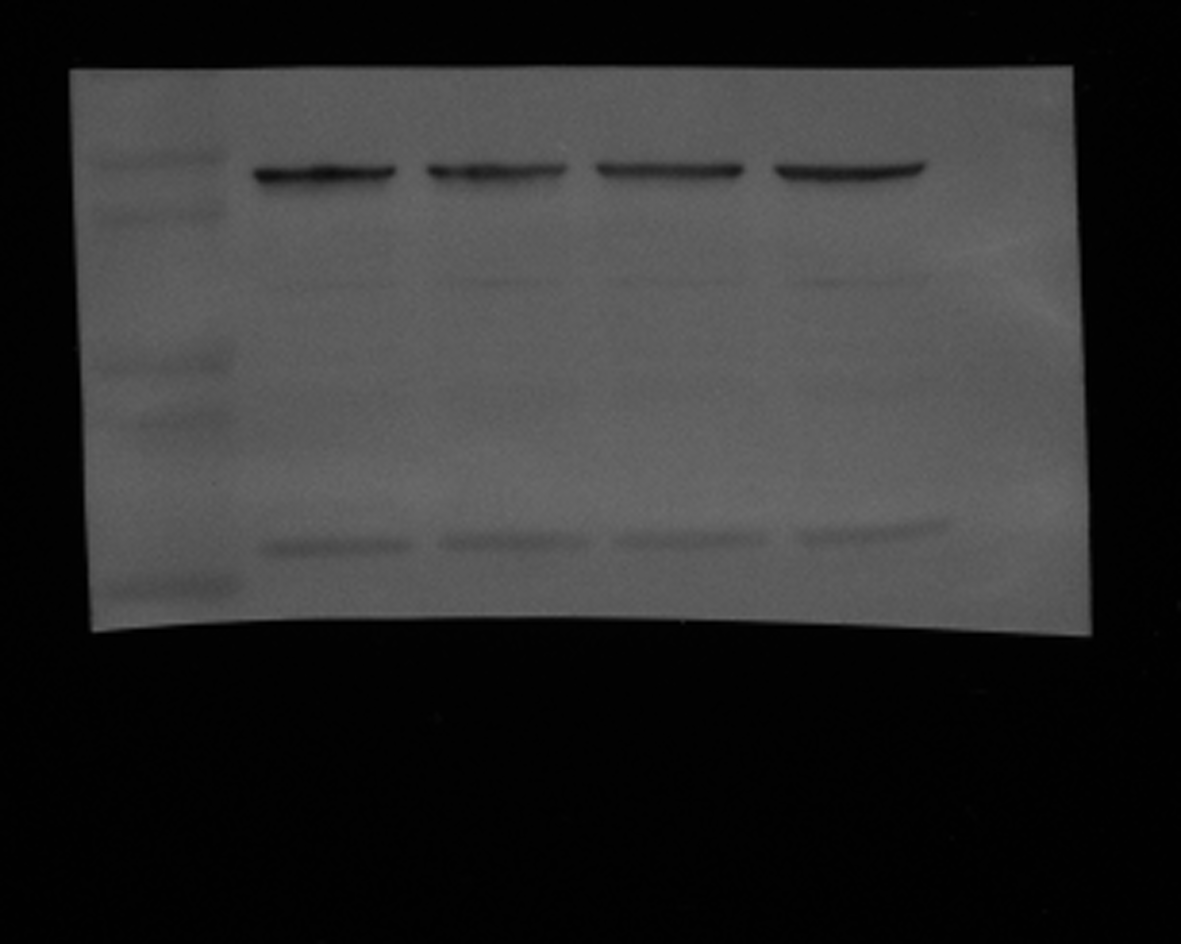

Supplement: Supplementary file 1 [file biomolecules-16-00926-s001.zip › biomolecules-4345458-WB/WB/Antioxidant/KEAP-1/membrane1/Gapdh/CHEMI_09302025_190346_(Overlay).tif]

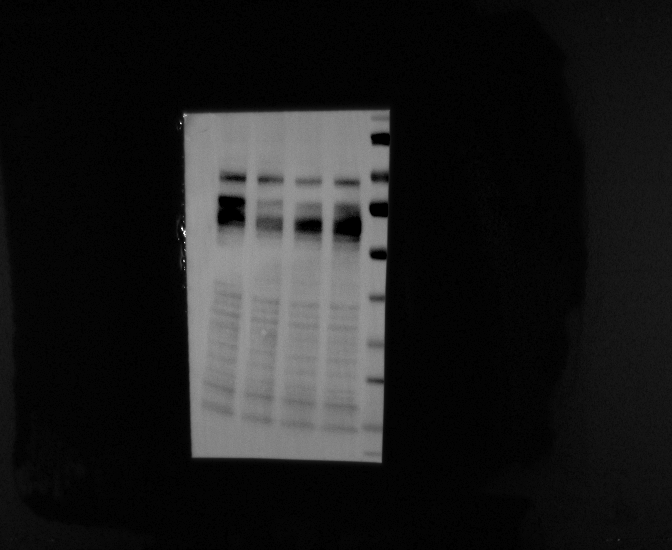

Supplement: Supplementary file 1 [file biomolecules-16-00926-s001.zip › biomolecules-4345458-WB/WB/Antioxidant/KEAP-1/membrane2/WB_20251031_185923_00.00_8bit.tif]

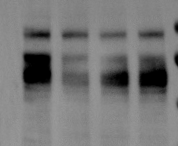

Supplement: Supplementary file 1 [file biomolecules-16-00926-s001.zip › biomolecules-4345458-WB/WB/Antioxidant/KEAP-1/membrane2/WB_20251031_190330_00.00_8bit-.tif]

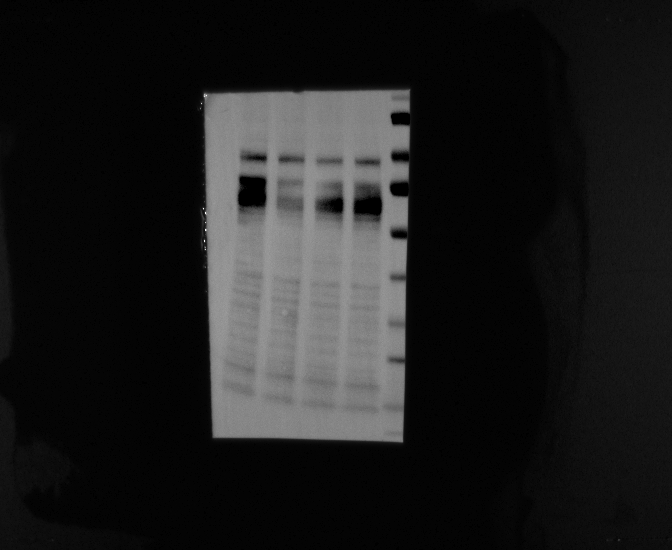

Supplement: Supplementary file 1 [file biomolecules-16-00926-s001.zip › biomolecules-4345458-WB/WB/Antioxidant/KEAP-1/membrane2/WB_20251031_190330_00.00_8bit.tif]

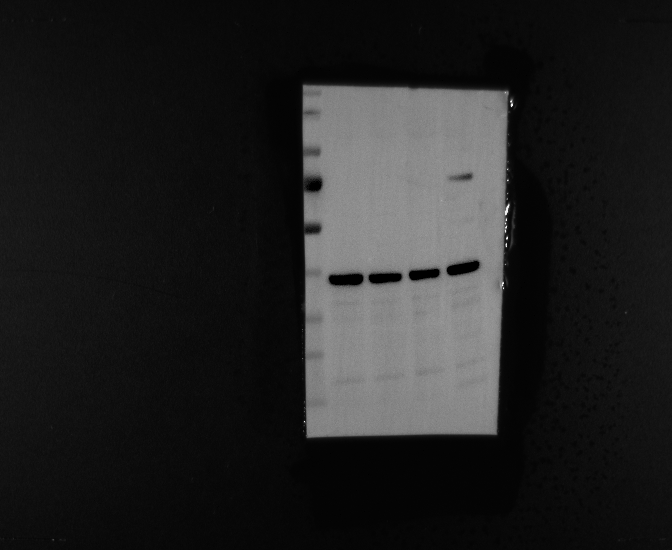

Supplement: Supplementary file 1 [file biomolecules-16-00926-s001.zip › biomolecules-4345458-WB/WB/Antioxidant/KEAP-1/membrane2/WB_20251105_185855_00.00_8bit.tif]

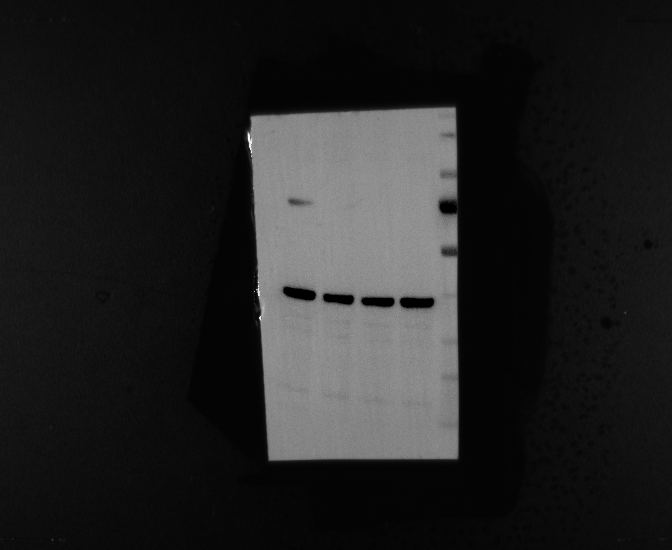

Supplement: Supplementary file 1 [file biomolecules-16-00926-s001.zip › biomolecules-4345458-WB/WB/Antioxidant/KEAP-1/membrane2/WB_20251105_190029_00.00_8bit.tif]

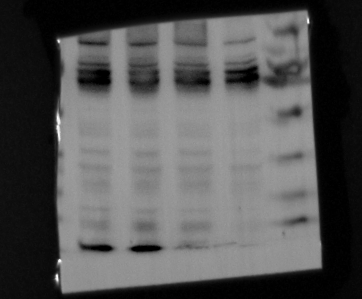

Supplement: Supplementary file 1 [file biomolecules-16-00926-s001.zip › biomolecules-4345458-WB/WB/Antioxidant/KEAP-1/membrane3/WB_20251020_123634_00.00_8bit.tif]

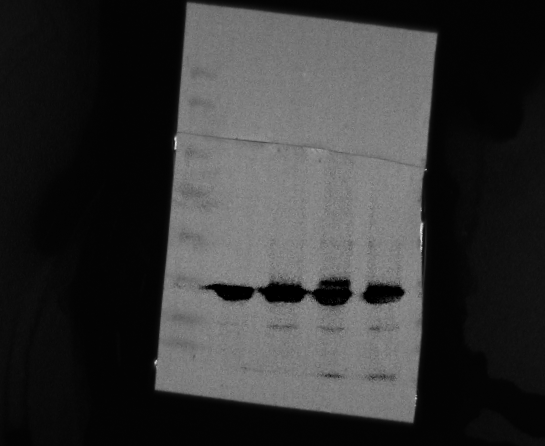

Supplement: Supplementary file 1 [file biomolecules-16-00926-s001.zip › biomolecules-4345458-WB/WB/Antioxidant/KEAP-1/membrane3/WB_20251028_124828_00.00_8bit.tif]

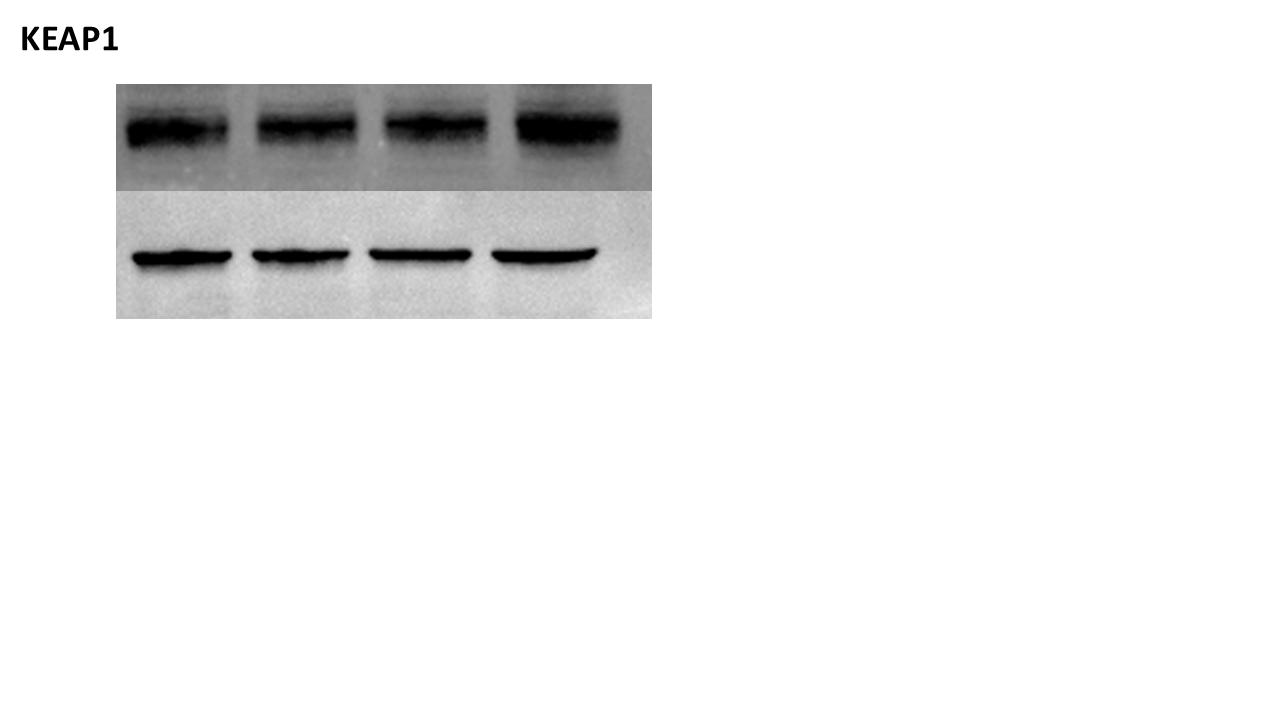

Supplement: Supplementary file 1 [file biomolecules-16-00926-s001.zip › biomolecules-4345458-WB/WB/Antioxidant/KEAP-1/WB.tif]
